# Secondary 3‐Chloropiperidines: Powerful Alkylating Agents

**DOI:** 10.1002/open.202300181

**Published:** 2023-12-13

**Authors:** Mats Georg, Lina Alexandra Laping, Veronica Billo, Barbara Gatto, Peter Friedhoff, Richard Göttlich

**Affiliations:** ^1^ Institute of Organic Chemistry Justus Liebig University Giessen Heinrich-Buff-Ring 17 35392 Giessen Germany; ^2^ Department of Pharmaceutical and Pharmacological Sciences University of Padova Padova Italy; ^3^ Institute of Biochemistry Justus Liebig University Giessen Heinrich-Buff-Ring 17 35392 Giessen Germany

**Keywords:** alkylation, antitumor agents, aziridine, primary chloroamine, secondary 3-chloropiperidine

## Abstract

In previous works, we demonstrated that tertiary 3‐chloropiperidines are potent chemotherapeutics, alkylating the DNA through the formation of bicyclic aziridinium ions. Herein, we report the synthesis of novel secondary 3‐chloropiperidine analogues. The synthesis incorporates a new procedure to monochlorinate unsaturated primary amines utilizing *N*‐chlorosuccinimide, while carefully monitoring the temperature to prevent dichlorination. Furthermore, we successfully isolated highly strained bicyclic aziridines by treating the secondary 3‐chloropiperidines with a sufficient amount of base. We conclude this work with a DNA cleavage assay as a proof of principle, comparing our previously known substrates to the novel compounds. In this, the secondary 3‐chloropiperidine as well as the isolated bicyclic aziridine, proved to be more effective than their tertiary counterpart.

## Graphical abstract

The synthesis of secondary 3‐chloropiperidines and highly strained bicyclic aziridines is reported, including a new method for the selective mono‐chlorination of unsaturated primary amines. The novel compounds, which closely resemble natural alkylating agents, proved to be more active than previously reported 3‐chloropiperidines in a DNA cleavage assay, highlighting their potential as powerful alkylating agents.

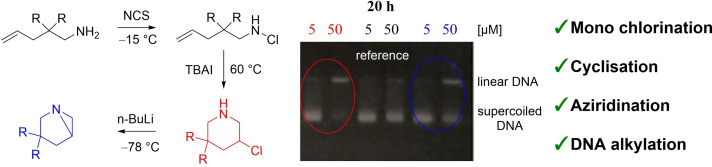

## Introduction

**Figure 1:**
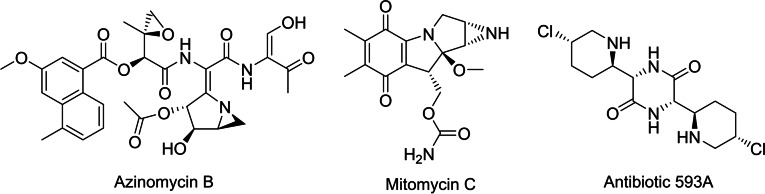
Natural compounds with known alkylating properties as lead structures.

**Figure 2:**
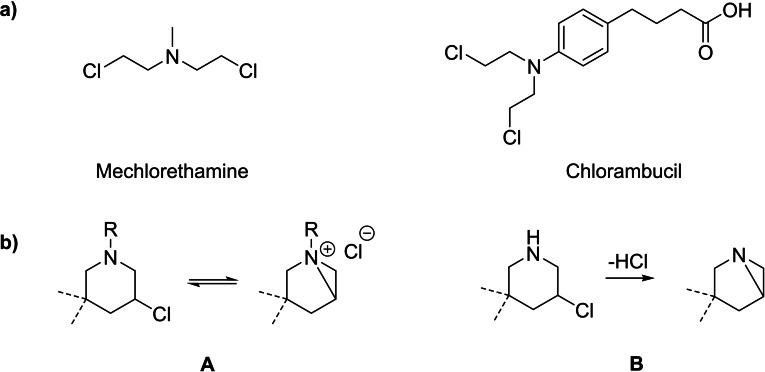
a) Structures of mechlorethamine and chlorambucil. b) General structure of tertiary 3‐chloropiperidines (A) and of secondary 3‐chloropiperidines (B).

Being the first class of chemotherapeutics, alkylating agents have played a major role in the treatment of cancer.^[1]^ One of the simplest representatives is mechlorethamine, which has been developed in the mid‐20^th^ century and is still in use today.[^2^, ^3^] Its mode of action involves the intramolecular formation of a highly reactive aziridinium ion, by substitution of the chloride. This electrophilic intermediate is readily attacked by nucleophiles like the guanine bases of the DNA.^[4]^ This results in the formation of a covalent adduct which potentially initiates further steps like depurination, strand cleavage and eventually apoptosis of the affected cell.^[5]^ Despite serious side effects, a wide variety of alkylating agents have been developed in the past and to this day they are indispensable therapeutics. Besides synthetic compounds like mechlorethamine or chlorambucil^[2]^ (Figure 2a), numerous natural analogues with similar modes of action have been discovered and isolated. In the context of this paper the most relevant compounds would be azinomycins, mitomycins as well as the antibiotic 593A (Figure 1).[^6^, ^7^, ^8^]

Especially the latter one shows a remarkable similarity to the synthetic nitrogen mustards with its active moiety being a 3‐chloropiperidine. Its total synthesis, however, has demonstrated to be very challenging.^[9]^ Therefore, in the past years, our research group has developed a multitude of simplified analogues. Previous publications have proven that these novel 3‐chloropiperidines are capable of alkylating the DNA in vitro and confirmed that they are preferentially attacked by the *N* ^7^‐Position of the guanine nucleobase.[^10^, ^11^] Our most recent advancements included the isolation of the bicyclic aziridinium ion as the key intermediate to gain further inside in the mechanism of action by NMR kinetics.^[12]^

Looking back at the antibiotic 593A (Figure 1) as the lead structure, one might quickly notice that it incorporates a 3‐chloropiperidine containing a secondary amine. In our previous studies we reported the synthesis and evaluation of tertiary 3‐chloropiperidines (Figure 2b, A), using the nitrogen as a simple and convenient position to modify the structure. To resemble the lead structure more closely, in the present work we focus on the synthesis and characterisation of secondary 3‐chloropiperidines (Figure 2b, B). In contrast to previous compounds, we expect that these novel alkylating agents could form the stable aziridines instead of the highly reactive aziridinium ions.^[12]^ Their stability should make them less prone to side reactions such as hydrolysis and ultimately lead to a higher concentration of the active intermediate. Aziridines are also common in a variety of natural compounds and serve as the active moiety in clinical used compounds such as mitomycin C and azinomycin B (Figure 1).[^6^, ^7^] Taking this into account,t we hypothesized that, despite being chemically less reactive, secondary 3‐chloropiperidines possibly represent more potent alkylating agents then their tertiary counterparts.

## Results and Discussion

**Scheme 1:**
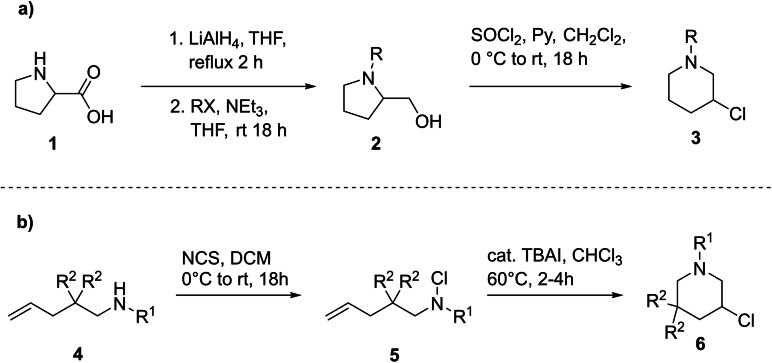
a) Pathway 1, starting from d‐ or l‐proline resulting in the respective enantiopure 3‐chloropiperidine; b) pathway 2, starting from a linear amine precursor, which is chlorinated to the corresponding *N*‐chloroamine followed by TBAI‐mediated cyclisation.

In our previous publications, we have established two main synthetic pathways towards 3‐chloropiperidines.^[11]^ The first approach (Scheme 1) is utilizing the already cyclic amino acid proline **1**, which in the first step is reduced to proline with lithium aluminium hydride and functionalized at the nitrogen through a nucleophilic substitution. In the final step, the pyrrolidine precursor 2 is chlorinated with thionyl chloride to the thermodynamically more stable 3‐chloropiperidine **3**, which is formed via a ring expansion reaction utilizing the aforementioned aziridinium ion. Since the ring expansion proceeds in a stereoselective way this approach can lead to enantiopure compounds, whose stereochemistry depends on the choice of the starting material, i. e. either d‐ or l‐proline.[^13^, ^14^] While this pathway bears obvious advantages it lacks the opportunity of any further functionalization of the 3‐chloropiperidine.

The second synthetic approach starts from a linear amine precursor **4**, which is chlorinated utilizing NCS and then transformed into the desired product **6** via an Iodine catalysed cyclisation (Scheme 1b). While leading to stereoisomers, this route allows the introduction of various different groups in the R^2^‐position, such as methyl, phenyl or even cyclic groups like cyclopropanyl and cyclobutanyl.^[15]^

**Scheme 2:**
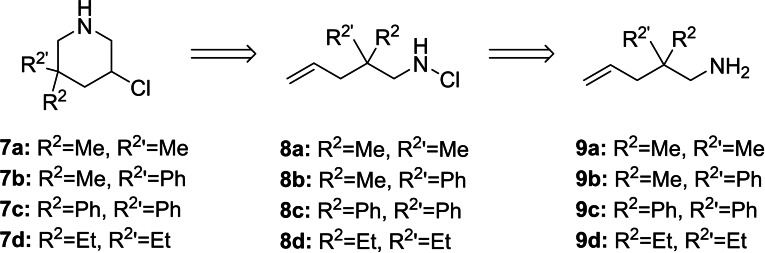
Planned synthetic pathway, adapted from pathway 2.

**Scheme 3:**
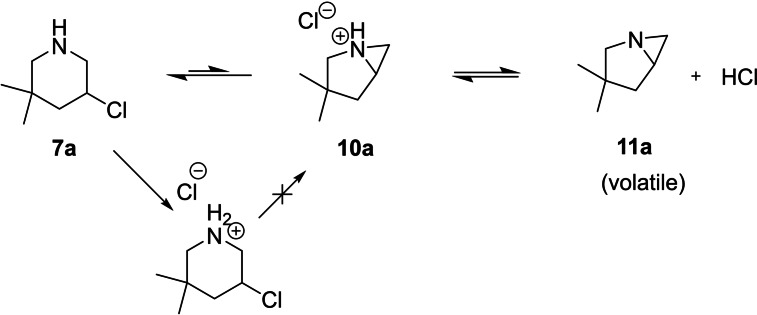
Formation of the azirinium ion, followed by the release of HCl to the aziridine.

The primary amine **9 a** used as a starting material in this approach (Scheme 2) is readily available through a literature known procedure from 2,2‐dimethylpent‐4‐enal.^[16]^ Transformation to the oxime and subsequent reduction with LAH gives the desired amine.^[17]^ The mono chlorination to the cyclisation precursor **8 a** posed a bigger challenge. Although guillemin described a gas/solid phase based procedure to obtain mono‐chlorinated primary amines, he also reported that unsaturated amines are too unstable to be chlorinated under his conditions.^[18]^ Utilizing the same conditions as described above in Scheme 1b inadvertently led to the unwanted *N,N*‐dichloroamine. While the desired product could be observed in a TLC reaction control, it was not possible to isolate it through column chromatography. Finally, a reduction of the reaction temperature to −15 °C, plus adjustment of the used equivalents, combined with a careful monitoring via TLC to prevent disproportionation, led to a full conversion into the sought‐after product **8 a**. The succinic imide, formed as a side product, could be filtered off after precipitation with pentane, resulting in very high yields without the need for any further purification. With the precursor **8 a** at hand we turned to the final cyclisation. While multiple catalysts (TBAI, CuCl_2_ and BF_3_ ⋅ Et_2_O) led to a conversion, which could be observed with GC/MS, it was not possible to isolate the desired product **7 a**. We reasoned this might be due to the formation of the possibly volatile aziridine **11 a** (Scheme 3).

To circumvent this problem, we increased the molecular weight of the product by introducing phenyl groups in the R^2^‐Position. The necessary starting material **9 b** can be obtained as described above.[^16^, ^17^] The diphenyl substituted amine **9 c** can be produced starting from commercially available diphenylacetonitrile by allylation and reduction.^[19]^ This attempt proved to be successful and compounds **7 b** and **7 c** could be isolated over two steps in 44 % and 51 % yield respectively.

Transforming 3‐chloropiperidines into their HCl salts effectively brings their reactivity to a halt, by lowering the electron density at the nitrogen centre and therefore stopping the formation of the aziridinium ion. This led to an optimized approach, trapping the cyclized product in situ with a suitable reagent, which lowers the electron density of the nitrogen. First experiments were done with acetic anhydride, giving the acetylated piperidine in 92 % yield. In order to release the trapping agent more easily, we switched to Boc‐anhydride, which after purification via column chromatography can easily be removed with HCl ⋅ dioxane leading directly to the stable HCl salt in nearly quantitative yields.

**Scheme 4:**
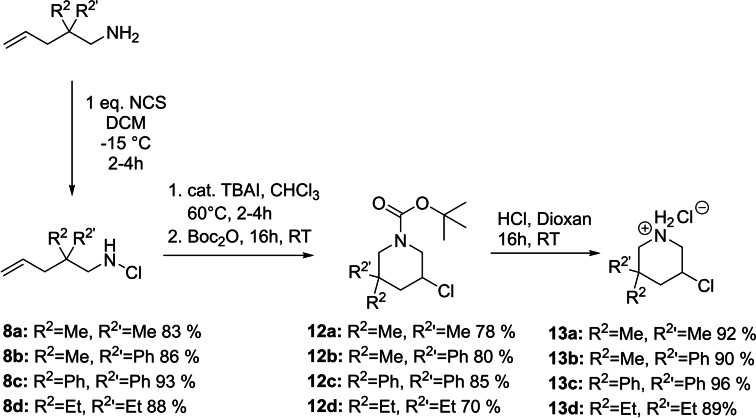
Optimized synthetic pathway leading directly to the storable HCl salts.

In summary this optimized synthetic pathway gives easy access to a variety of novel 3‐chloropiperidines incorporating a secondary amine in good yields up to >90 %. Due to the unspecific cyclisation all products are obtained racemic, with the exception of **13 b** giving two diastereomers that can be separated through column chromatography prior to deprotection. The synthesis of diphenyl functionalized compound **12 c** also led to the isolation of the corresponding 2‐(chloromethyl)pyrrolidine in 19 % yield, bringing the cyclisation to a combined yield of 85 % (Scheme 4).

**Scheme 5:**
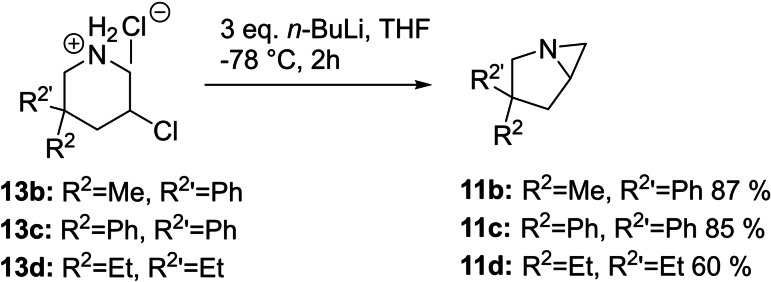
Synthesis of bicyclic aziridines from secondary 3‐chloropiperidine.

The aforementioned azinomycin B (Figure 1) also has been shown to alkylate DNA with its two active centres, one of them being a bicyclic aziridine,^[20]^ similar to compound **11 a** shown in Scheme 3. This observation, together with the high synthetic value of aziridines,^[21]^ sparked our interest in accessing bicyclic aziridines from the secondary 3‐chloropiperidines discussed above. Multiple sources have shown that treating β‐chlorinated amines with sufficient base leads to the formation of aziridines.^[22]^ With compounds **13 a**–**d** at hand we turned our attention to finding suitable reaction conditions. Treating compounds **13 b**–**d** with a slight excess of *n*‐BuLi at −78 °C in THF, followed by an aqueous workup resulted in good yields up to 87 % without the need for any further purification (Scheme 5). Compound **11 a** could not be isolated, which is attributed to its low molecular weight and therefore low boiling point.

With the availability of the novel compounds better resembling the lead natural compound, we examined their reactivity in comparison to our previously published tertiary amine compound **6**. A classical DNA cleavage assay was chosen, adopting the workflow employed in our previous publications. Briefly, single‐ or double‐strand breakage of DNA, as a follow up reaction to previous alkylation, leads to a change in the topology of a supercoiled (SC) DNA plasmid. The resulting open chain (OC) or linearized (L) forms show a different electrophoretic mobility in agarose gel relative to the control unreacted DNA. This evidence can be used to evaluate the ability to alkylate DNA of our novel compounds. DNA plasmid pGEM1 was incubated with two distinct concentrations (5 and 50 μM) of the 3‐chloropiperidine or the respective aziridine in a BPE (biphosphate‐EDTA) buffer solution at 37 °C. While compounds containing two methyl groups in the R^2^ position have previously been in the focus of our research, compounds **7 c**, **11 c** and **6 c** have been chosen for this proof of principle, allowing us to also evaluate the reactivity of the bicyclic aziridine.3

**Figure 3:**
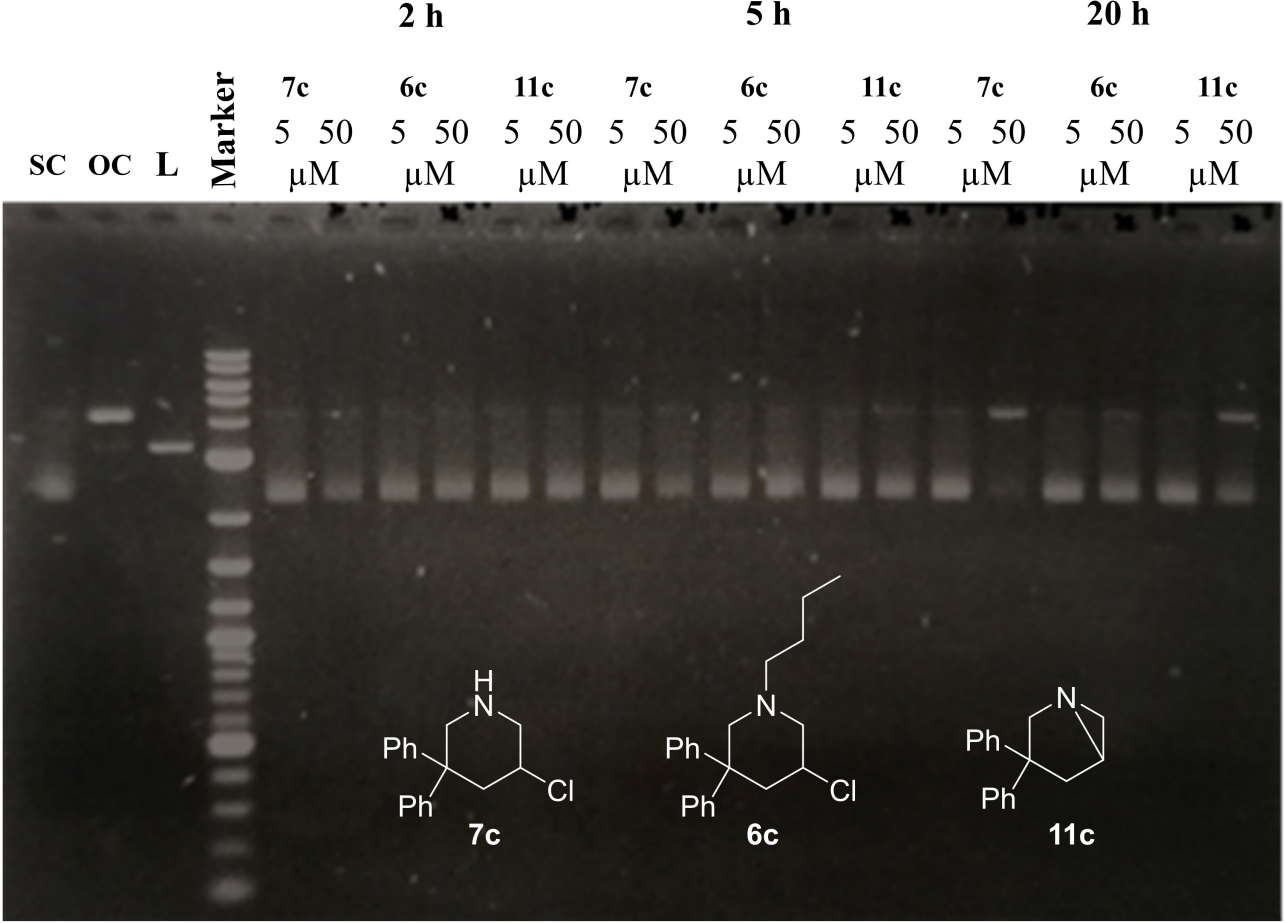
DNA cleavage activity comparison between **7 c**, **14 c** and **11 c** in two different concentrations (5 μM and 50 μM). The supercoiled pGEM1 was incubated for 2 h, 5 h and 20 h at 37 °C in BPE buffer. Cleavage of supercoiled DNA into its open circular (OC) and linearized (L) forms was monitored utilizing gel electrophoresis (1.4 % agarose in TAE buffer). Visualization was achieved upon staining with Intas HDGreen^TM^ under UV‐light.

While in the lower concentration of 5 μM none of the compounds show differences in cleavage activity to allow for a clear distinction of potency, at the higher concentration of 50 μM and prolonged incubation time of 20 h substances **7 c** and **11 c** proved to be much more active than their tertiary counterpart **6 c**. **7 c** seems to be slightly more active than **11 c**, which is somewhat surprising considering that **11 c** is believed to be an intermediate in the reaction of **7 c** with nucleophiles. A possible explanation for this might be a premature hydrolysation of the highly reactive aziridine. These results demonstrate that the novel compounds are capable of alkylating and cleaving DNA, and also proves our initial hypothesis regarding the reactivity of secondary 3‐chloropiperidines in comparison to our previously published compounds.^[11]^

## Conclusions

In conclusion we report the synthesis of novel 3‐chloropiperidines incorporating a secondary amine. In the first step, we developed a new and easy method for the mono‐chlorination of unsaturated primary amines with NCS at lowered temperatures. The cyclisation to the desired 3‐chloropiperidines proceeded through the use of catalytic amounts of TBAI. Our method utilizing Boc‐anhydride to trap the product in situ allows for an easy workup and prevents the formation of highly reactive and volatile side products. The protection group can afterwards be cleaved easily with HCl ⋅ dioxane, to give the unreactive and therefore storable HCl salts in almost quantitative yields, without the need for any further purification. Treatment with 2 equivalents of *n*‐BuLi gave the synthetically interesting bicyclic aziridines in good yields over 80 %. Finally, we concluded this work proving by DNA alkylating assay that the secondary 3‐chloropiperidine as well as the corresponding aziridine showed a higher activity compared to previously reported compounds. In the future we plan on further investigating the alkylating properties of the novel secondary 3‐chloropiperidines and the isolated bicyclic aziridines, as well as exploring the synthetic applications of the mono‐chlorinated primary amines.

## Experimental Section

### 2,2‐Dimethylpen‐4‐enal

Freshly distilled isobutyraldehyde (109.508 g, 1.50 mol, 1.5 equiv.), allyl alcohol (59.121 g, 1.0 mol, 1 equiv.) and *p*‐toluenesulfonic acid (0.263 g, 1.50 mmol) are added in *p*‐cymene (200 g). The mixture is heated at reflux for 24 h under a Dean–Stark apparatus, until no more water is separated and the temperature at the reaction sump reached 140 °C. The product is separated as a colourless liquid in 73 % yield (82.339 g, 0.73 mol) by vacuum distillation through a 50 cm Vigreux column (62–69 °C at 150 mbar). ^1^H NMR (400.1 MHz, CDCl_3_): 9.46 (s, 1H), 5.76–5.62 (m, 1H), 5.10–4.98 (m, 2H), 2.20 (dt, *J*=7.5, 1.2 Hz, 2H), 1.04 (s, 6H). ^13^C NMR (100.6 MHz, CDCl_3_): δ [ppm]=205.88, 133.13, 118.45, 45.72, 41.44, 21.16. HRMS (ESI): *m/z*=135.0783 [M+Na]^+^ (calculated for C_7_H_12_O+Na^+^: 135.0780).

### 2,2‐Dimethylpent‐4‐enal oxime

A solution of hydroxylammonium chloride (15.04 g, 216.0 mmol, 1.2 equiv.) in 20 mL of distilled water and a solution of sodium hydroxide (7.23 g, 181.0 mmol, 1 equiv.) in 80 mL of distilled water/ethanol 1 : 3 are added to a solution of 2,2‐dimethylpent‐4‐enal (20.26 g, 181 mmol, 1 equiv.) in 300 mL of ethanol. The mixture is left standing for 16 h at room temperature. The ethanol is removed under reduced pressure and 300 mL of TMBE are added to the mixture. The phases are separated, and the aqueous phase is extracted by the addition of TMBE (3×100 mL) and the organic phase is then dried with MgSO_4_. The solvent is removed under reduce pressure and the crude product is purified by vacuum distillation (94 °C at 40 mbar). The product is obtained in 81 % yield (18.696 g, 0.147 mol).^1^H NMR (400 MHz, CDCl_3_): δ [ppm]=9.00 (br s, 1H), 7.34 (s, 1H), 5.81–5.70 (m, 1H), 5.10–5.02 (m, 2H), 2.16 (dt, *J*=7.4, 1.2 Hz, 2H), 1.09 (s, 6H). ^13^C NMR (100.6 MHz, CDCl_3_): δ [ppm]=158.68, 133.99, 118.27, 45.36, 36.76, 25.13 (ESI): *m/z*=150.0890 [M+Na]^+^ (calculated for C_7_H_13_NO+Na^+^: 150.0889).

### 2,2‐Dimethylpent‐4‐en‐1‐amine (9 a)

Under nitrogen atmosphere, to a suspension of LiAlH_4_ (8.38 g, 221.0 mmol, 1.5 equiv.) in 200 mL of anhydrous diethyl ether, a solution of 2,2‐dimethylpent‐4‐enal oxime (18.696 g, 147.0 mmol, 1.0 equiv.) in 80 mL of anhydrous diethyl ether is added dropwise at 0 °C. The mixture is stirred at room temperature for 18 h. The latter is then cooled down to 0 °C and carefully quenched with water (8.40 mL), 15 % NaOH solution (8.40 mL), and then water again (25 mL). The mixture is stirred for 30 min at 0 °C and filtered. The precipitate is suspended again in diethyl ether, heated to reflux for 30 min, and filtered while it is still warm. The combined organic filtrate is dried over Na_2_SO_4_ and the solvent is removed under reduced pressure. The product is purified by vacuum distillation (50–60 °C at 80 mbar), obtaining a colourless oily liquid in 56 % yield (9.347 g, 82.6 mmol). ^1^H NMR (400.1 MHz, CDCl_3_) : δ [ppm]=5.75 (ddt, *J*=15.9, 11.3, 7.5 Hz, 1H), 4.99–4.92 (m, 2H), 2.38 (s, 1H), 1.90 (dt, *J*=7.5, 1.2 Hz), 0.98 (s), 0.79 (s). ^13^C NMR (100.6 MHz, CDCl_3_): δ [ppm]=135.33, 116.90, 52.65, 44.01, 34.89, 24.59. HRMS (ESI): *m/z*=114.1277 [M+H]^+^ (calculated for C_7_H_15_N+H^+^: 114.1277).

### N‐Chloro‐2,2‐dimethylpent‐4‐en‐1‐amine (8 a)

2,2‐dimethylpent‐4‐en‐1‐amine (1.136 g, 10.00 mmol, 1.0 equiv.) is added to a solution of dry DCM (70 mL) under nitrogen atmosphere. The solution is cooled down to −15 °C (ethylene glycol/liquid nitrogen bath) and then 1 equiv. of NCS is added (1.333 g, 10.00 mmol, 1 equiv.). The mixture is stirred for 2–4 h at −15 °C. When a TLC control (pentane/TBME 10 : 1) confirms the end of the reaction, the solvent is evaporated. 50 mL of pentane are added to make the succinic imide precipitate. The solution is filtered and the solvent is removed under reduced pressure resulting in a pale yellow oil in 83 % yield (1.227 g, 8.31 mmol). ^1^H NMR (400.1 MHz, CDCl_3_):δ [ppm]=5.81–5.69 (m, 1H), 5.03–4.94 (m, 2H), 4.27–3.93 (m, 1H), 2.83 (t, *J*=2.8 Hz, 2H), 1.98–1.94 (m, 2H), 0.88 (s, 6H).^13^C NMR (100.6 MHz, CDCl_3_):δ [ppm]=134.57, 117.72, 67.15, 44.66, 35.55, 25.35. HRMS (ESI): *m/z*=148.0890 [M+H]^+^ (calculated for C_7_H_14_ClN+H^+^: 148.0888).

### N‐Boc‐3‐chloro‐5,5‐dimethylpiperidine (12 a)

Under nitrogen atmosphere, 230 mg (1.56 mmol, 1 equiv.) of *N*‐chloro‐2,2‐dimethylpent‐4‐en‐amine is dissolved in 15 mL of anhydrous CHCl_3_. 0.408 g of TBAI (0.31 mmol, 0.2 equiv.) is added to the solution at room temperature. The mixture is heated at 60 °C for 3 h. Boc‐anhydride (1.87 g, 1.87 mmol, 1.2 equiv.) is added to the solution at room temperature. The mixture is stirred for 16 h at room temperature. After purification by column chromatography (pentane/TBME, 10 : 1) the product is isolated as a mixture of rotamers as colourless crystals in 78 % yield (300 mg, 1.21 mmol) ^1^H NMR (400.1 MHz, CDCl_3_): δ [ppm]=4.59–4.24 (m, 1H), 3.96 (tt, *J*=11.5, 4.6 Hz, 1H), 3.86–3.54 (m, 1H), 2.75–2.55 (m, 1H), 2.54–2.34 (m, 1H), 2.02–1.95 (m, 1H), 1.49 (d, *J*=12.4 Hz, 1H), 1.45 (s, 9H), 0.94 (s, 6H). ^13^C NMR (100.6 MHz, CDCl_3_): δ [ppm]=154.55, 80.01, 54.96, 51.97, 50.66, 48.69, 34.13, 28.37, 28.25, 23.53. HRMS (ESI): *m/z=*270.1229 [M+Na]^+^ (calculated for C_12_H_22_ClNO+Na^+^: 270.1231).

### 3‐Chloro‐5,5‐dimethylpiperidine hydrochloride (13 a)

150 mg (0.60 mmol, 1 equiv.) *N*‐Boc‐3‐chloro‐5,5‐dimethylpiperidine are dissolved in 5 mL of dioxane and 3 mL 4 n HCl*dioxane are added. The solution is stirred for 16 h at room temperature. 5 mL of diethyl ether are added and the suspension is centrifuged. The resulting white solid is washed two times with diethyl ether and dried in vacuo. The product is obtained in 92 % yield (102 mg, 0.55 mmol). ^1^H NMR (400.1 MHz, CDCl_3_): δ [ppm]=10.33 (s, 1H), 9.48 (s, 1H), 4.37–4.24 (m, 1H), 3.77–3.68 (m, 1H), 3.07 (d, *J*=12.9 Hz, 1H), 2.82–2.69 (m, 1H), 2.71–2.60 (m, 1H), 2.15–2.06 (m, 1H), 1.57 (t, *J*=12.7 Hz, 1H), 1.26 (s, 3H), 1.07 (s, 3H). ^13^C NMR (100.6 MHz, CDCl_3_): δ [ppm]=^13^C NMR (101 MHz, CDCl_3_) δ 49.25, 48.06, 46.33, 32.05, 29.01, 24.38.

### 2‐Methyl‐2‐phenylpent‐4‐enal

12.63 g (217.38 mmol, 1.07 equiv.) allylic alcohol and 27.76 g (203.16 mmol, 1 equiv.) 2‐phenyl‐propionaldehyde are dissolved in 15 mL of toluene in a 100 mL two necked flask with an attached 30 cm Vigreux column and a Dean–Stark trap. 0.103 g (0.6 mmol, cat.) *p*‐toluenesulfonic are added and the mixture is refluxed, until 3.7 mL of water have been released. The crude product is distilled directly from the mixture at 3 mbar and 77 °C. The product is obtained in 83 % yield (29.522 g, 169.4 mmol). ^1^H NMR (400.1 MHz, CDCl_3_): δ [ppm]=9.62 (s), 7.41–7.35 (m), 7.31–7.23 (m), 5.60–5.45 (m), 5.09–5.00 (m), 2.73–2.59 (m), 1.44 (s) ^13^C NMR (100.6 MHz, CDCl_3_): δ [ppm]=δ 202.09, 139.62, 133.31, 128.99, 127.47, 127.31, 118.73, 53.75, 40.74, 18.98 HRMS (ESI): *m/z*=193.0935 [M+Na]^+^ (calculated for C_12_H_14_O+Na^+^: 197.0937).

### 2‐Methyl‐2‐phenylpent‐4‐enal oxime

A solution of 6.25 g (90 mmol, 1.2 equiv.) of hydroxylamine hydrochloride in 6.6 mL of water is added to a solution of sodium hydroxide (3.0 g, 75 mmol, 1 equiv.) in water/ethanol (7 mL/25 ml). 2‐methyl‐2‐phenylpent‐4‐enal (13.07 g, 75 mmol, 1 equiv.) is dissolved in 170 mL of ethanol and added to the mixture. The latter is left stirring overnight. 350 mL of TBME are added and the phases are separable. The organic phase is washed with brine and then dried over MgSO_4_. After evaporation of the solvent, the product is obtained in 91 % yield (12.97 g, 68.55 mmol). ^1^H NMR (400.1 MHz, CDCl3): δ [ppm]=8.85 (br s), 7.48 (s), 7.32–7.22 (m), 7.21–7.15 (m), 5.54 (ddt, J=17.3, 10.2, 7.2 Hz), 5.02–4.95 (m), 2.59 (d, J=1.1 Hz), 1.41 (s). ^13^C NMR (100.6 MHz, CDCl_3_): δ [ppm]=157.24, 143.88, 133.74, 128.53, 126.75, 126.62, 118.41, 43.98, 40.62, 23.45. HRMS (ESI): *m/z*=190.1042 [M+H]^+^ (calculated for C_12_H_15_NO+H^+^: 190.1227).

### 2‐Methyl‐2‐phenylpent‐4‐en‐1‐amine (9 b)

Under nitrogen atmosphere, 3.90 g (103 mmol, 1.5 equiv.) of lithium aluminium hydride are suspended in 125 mL of dry diethyl ether at 0 °C. 12.94 g (68.37 mmol, 1 equiv.) of 2‐methyl‐2‐phenylpent‐4‐enal oxime are dissolved in 50 mL of anhydrous diethyl ether and added carefully to the LAH suspension at 0 °C. The mixture is stirred at room temperature for 16 h. The mixture is quenched with 3.76 mL of water, 3.76 mL of 20 % NaOH_aq_ and 11.28 mL of water at 0 °C. After filtration the solid is resuspended and heated to reflux in 100 mL TBME. After filtration the organic phases are combined and evaporated. The crude product is purified by vacuum distillation (100–105 °C at 3 mbar) obtaining a colourless liquid in 75 % yield (8.99 g, 51.3 mmol). ^1^H NMR (400.1 MHz, CDCl_3_): δ [ppm]=7.38–7.29 (m), 7.24–7.17 (m), 5.63–5.50 (m), 5.06–4.95 (m), 2.96 (d, *J*=13.1 Hz), 2.73 (d, *J*=13.2 Hz), 2.52 (ddt, *J*=13.8, 6.4, 1.5 Hz), 2.28 (ddt, *J*=13.8, 8.1, 1.1 Hz), 1.30 (s). ^13^C NMR (100.6 MHz, CDCl_3_): δ [ppm]=134.75, 128.36, 126.74, 125.96, 117.31, 53.37, 44.60, 22.21. HRMS (ESI): *m/z*=176.1438 [M+H]^+^ (calculated for C_12_H_17_N+H^+^: 176.1434).

### N‐Chloro‐2‐methyl‐2‐phenylpent‐4‐en‐1‐amine (8 b)

2‐Methyl‐2‐phenylpent‐4‐en‐1‐amine (1.753 g, 10 mmol, 1.0 equiv.) is added to a solution of dry DCM (70 mL) under nitrogen atmosphere. The solution is cooled down to −15 °C and 1 equiv. of NCS is added (1.335 g, 10 mmol, 1 equiv.). The mixture is stirred for 2 h at −15 °C. When a TLC control (pentane/TBME 10 : 1, Rf=0.54) confirms the end of the reaction, the solvent is evaporated. 50 mL of pentane are added to make succinimide precipitate. The solution is filtered and evaporated to obtain the product as a pale yellow oil in 86 % yield. ^1^H NMR (400.1 MHz, CDCl_3_): δ [ppm]=7.34–7.26 (m, 4H), 7.21–7.16 (m, 1H), 5.50 (dddd, *J*=16.8, 10.1, 7.9, 6.6 Hz, 1H), 5.03–4.92 (m, 2H), 3.77–3.66 (m, 1H), 3.36–3.28 (m, 1H), 3.16–3.07 (m, 1H), 2.51–2.44 (m, 1H), 2.36–2.29 (m,1H), 1.34 (s, 3H). ^13^C NMR (100.6 MHz, CDCl3): δ [ppm]=144.03, 133.81, 128.73 (d, *J*=2.5 Hz, C‐9 or C‐10), 126.59, 126.30, 118.20, 67.23, 45.18, 42.57, 23.06. HRMS (ESI): *m/z*=210.1048 [M+H]^+^ (calculated for C_12_H_16_ClN+H^+^: 210.1044).

### 3‐Chloro‐5‐methyl‐5‐phenylpiperidine (7 b)

Under nitrogen atmosphere, 1.750 g (8.34 mmol, 1 equiv.) of *N*‐chloro‐2‐methyl‐2‐phenylpent‐4‐en‐amine is dissolved in 30 mL of anhydrous chloroform. 0.308 g of TBAI (0.83 mmol, 0.1 equiv.) are added to the solution at room temperature. The mixture is heated to 60 °C for 3 h. The product is purified by flash chromatography (DCM/acetone 20 : 1, Rf=0.13) and obtained as a mixture of diastereomers (762 mg. 44 %). *cis*‐Diastereomer: ^1^H NMR (400.1 MHz, CDCl_3_): δ [ppm]=7.35–7.23 (m), 7.20–7.12 (m), 3.75–3.64 (m), 3.49 (ddd, *J*=13.9, 2.8, 1.0 Hz), 3.22–3.14 (m), 2.87–2.79(m), 2.67–2.60 (m), 2.58–2.53 (m), 1.75 (dd, *J*=13.4, 12.1 Hz), 1.06 (s).^13^C NMR (100.6 MHz, CDCl_3_): δ [ppm]=144.78, 129.11, 126.28, 126.13, 55.21, 54.51, 54.17, 46.96, 42.15, 31.33. *trans*‐Diastereomer: ^1^H NMR (400.1 MHz, CDCl_3_): δ [ppm]=7.35–7.23 (m), 7.20–7.12 (m), 4.15–4.05 (m), 3.39–3.33 (m), 2.97–2.92 (m), 2.79–2.74 (m) 2.59–2.55 (m), 2.40–2.33 (m), 1.97–1.91 (m), 1.33 (s). ^13^C NMR (100.6 MHz, CDCl3): δ [ppm]=147.82, 128.52, 126.44, 124.96, 55.65, 54.51, 54.24, 46.57, 40.25, 24.08. HRMS (ESI): *m/z*=210.1043 [M+H]^+^ (calculated for C_12_H_16_ClN+H^+^: 210.1044).

### 5‐Methyl‐5‐phenyl‐1‐azabicycle[3.1.0]hexane (11 b)

Under nitrogen atmosphere, 0.212 g (0.86 mmol, 1 equiv.) of 3‐chloro‐5‐methyl‐5‐phenylpiperidine hydrochloride are dissolved in 10 mL of dry THF. The solution is cooled down at −78 °C with an acetone/liquid nitrogen bath. 2 equiv. of *n*‐BuLi (0.70 mL, 1.72 mmol) are added carefully. The reaction mixture is stirred at −78 °C for 2 h and then quenched with 5–10 mL of NH_4_Cl. Diethyl ether is added and the phases are separated. The latter is extracted with diethyl ether (3×25 mL) and combined organic phases are dried over MgSO_4_ and evaporated. The product is obtained in 87 % yield (0.103 g, 0.75 mmol). ^1^H NMR (400.1 MHz, CDCl_3_): δ [ppm]=7.40–7.29 (m), 7.22–7.16 (m), 3.68 (d, *J*=12.2 Hz), 2.71 (d, *J*=12.2 Hz), 2.54–2.47 (m), 2.51–2.47 (m), 2.10–2.08 (m), 2.07–2.05 (m), 1.60–1.57 (m), 1.41 (s). ^13^C NMR (100.6 MHz, CDCl_3_): δ [ppm]=150.65, 128.37, 125.76, 125.63, 69.07, 52.93, 42.96, 42.23, 40.53, 30.82. HRMS (ESI): *m/z*=174.1274 [M+H]^+^ (calculated for C_12_H_15_N+H^+^: 174.1277).

### 2,2‐Diphenylpent‐4‐en‐nitrile

A solution of diphenylacetonitrile (10.013 g, 51.83 mmol, 1.0 equiv.) in 30 mL of anhydrous THF is added dropwise at 0 °C to a suspension of sodium hydride (2.714 g, 67.37 mmol, 1.3 equiv.) in anhydrous THF (20 mL) under nitrogen atmosphere. The mixture is stirred for 1 h at room temperature. 4.9 mL of allyl bromide (57.01 mmol, 1.1 equiv.) are added dropwise at 0 °C and the mixture is stirred for 2 h at room temperature. After the careful addition of NH_4_Cl (40 mL), the organic phase is separated by extraction of the aqueous phase with diethyl ether (3×25 mL). The organic phase is dried over MgSO_4_ and distilled in the Kugelrohr. The product is obtained in 99 % yield (11.951 g, 51.22 mmol) as a colourless oil. ^1^H NMR (400.1 MHz, CDCl_3_): δ [ppm]=7.44–7.26 (m, 10H), 5.80–5.65 (m, 1H), 5.33–5.14 (m, 2H), 3.19–3.11 (m, 2H). ^13^C NMR (100.6 MHz, CDCl_3_): δ [ppm]=139.77, 131.82, 128.89, 127.98, 127.09, 121.99, 120.44, 51.75, 43.97. HRMS (ESI): *m/z*=256.1098 [M+Na]^+^ (calculated for C_17_H_15_N+Na^+^: 256.1096).

### 2,2‐Diphenylpent‐4‐en‐1‐amine (9 c)

Under nitrogen atmosphere, 11.868 g (50.87 mmol, 1.0 equiv.) of 2,2‐diphenylpent‐4‐en‐nitrile **2** is dissolved in 60 mL of anhydrous diethyl ether and added dropwise to a suspension of LAH (3.513 g, 92.57 mmol, 1.8 equiv.) in 100 mL of anhydrous diethyl ether at 0 °C. The mixture is stirred for 18 h at room temperature. The blend is cooled down to 0 °C and water (3.5 mL), 20 % NaOH aqueous solution (3.5 mL) and water again (10.5 mL) are added carefully. Na_2_SO_4_ is added to the mixture and after filtration the solvent is removed und reduced pressure. The product is obtained as a colourless oil in 96 % yield (11.643 g, 49.05 mmol). ^1^H NMR (400.1 MHz, CDCl_3_): δ [ppm]=7.32–7.23 (m, 4H), 7.21–7.13 (m, 6H), 5.45–5.31 (m, 1H), 5.10–4.92 (m, 2H), 3.32 (s, 2H), 2.92 (dt, *J*=7.0, 1.3 Hz, 2H), 1.22 (s, 2H). ^13^C NMR (100.6 MHz, CDCl_3_): δ [ppm]=146.27, 134.67, 128.31, 128.22, 126.23, 117.90, 51.39, 48.59, 41.23. HRMS (ESI): *m/z*=238.1591 [M+H]^+^ (calculated for C_17_H_19_N+H^+^: 238.1590).

### N‐Chloro‐2,2‐diphenylpent‐4‐en‐1‐amine (8 c)

2,2‐Diphenylpent‐4‐en‐1‐amine **3** (2.718 g, 10 mmol, 1.0 equiv.) is added to a solution of dry DCM (70 mL) under nitrogen atmosphere. The solution is cooled down to −15 °C and 1 equiv. of NCS is added (1.336 g, 10 mmol, 1 equiv.). The mixture is stirred for 4 h at −15 °C. When a TLC control (pentane/TBME 10 : 1, Rf=0.61) confirms the end of the reaction, the solvent is evaporated. 50 mL of pentane are added to make succinic imide precipitate. The solution is filtered and evaporated to obtain the product in 93 % yield (2.530 g, 9.31 mmol). ^1^H NMR (400.1 MHz, CDCl_3_): δ [ppm]=7.34–7.21 (m, 7H), 7.18–7.15 (m, 4H), 5.41–5.30 (m, 1H), 5.14–5.01 (m, 2H), 3.73–3.67 (m, 2H), 2.99 (d, *J*=7.1 Hz, 2H). ^13^C NMR (100.6 MHz, CDCl_3_): δ [ppm]=145.20, 134.06, 128.48, 127.95, 126.73, 118.78, 62.90, 50.87, 41.94.

### 3‐Chloro‐5,5‐diphenylpiperidine (7 c)

Under nitrogen atmosphere, 9.150 g (30.3 mmol, 1 equiv.) of *N*‐chloro‐2,2‐diphenylpent‐4‐en‐amine **4** is dissolved in 40 mL of anhydrous CHCl_3_. 1.120 g of TBAI (3.03 mmol, 0.1 equiv.) are added to the solution at room temperature. The mixture is heated at 60 °C for 3 h. The product is purified by flash chromatography (DCM/acetone 50 : 1, Rf=0.13). 50 % Yield (4.220 g, 15.50 mmol) ^1^H NMR (400.1 MHz, CDCl_3_): δ [ppm]=7.34–7.01 (m, 10H), 3.85–3.81 (m, 1H), 3.81–3.76 (m, 2H), 3.29–3.21 (m, 1H), 3.16–3.09 (m, 1H), 2.95 (d, *J*= 14.1 Hz, 1H), 2.72 (dd, *J*=12.9, 11.1 Hz, 1H), 2.34 (dd, *J*=13.2, 12.0 Hz, 1H). ^13^C NMR (100.6 MHz, CDCl_3_): δ [ppm]=146.80, 143.00, 129.22, 128.50, 127.71, 126.59, 126.44, 126.11, 77.24, 54.37, 54.21, 53.99, 49.35, 45.71, 30.94. HRMS (ESI): *m/z=*272.1197 [M+H]^+^ (calculated for C_17_H_18_ClN+H^+^: 272.1201).

### N‐Boc‐3‐chloro‐5,5‐diphenylpiperidine (12 c)

Under nitrogen atmosphere, 1.00 g (3.68 mmol, 1 equiv.) of *N*‐chloro‐2,2‐diphenylpent‐4‐en‐amine is dissolved in 20 mL of anhydrous chloroform. 1.120 g of TBAI (0.37 mmol, 0.1 equiv.) are added to the solution at room temperature. The mixture is heated at 60 °C for 3 h. 2 equiv. of di‐*tert*‐butyldicarbonate (1.61 g, 7.36 mmol) are added at room temperature and the mixture is stirred for 16 h. 20 mL of water are added and the aqueous phase is extracted with DCM (3×25 mL) The organic phase is dried with MgSO_4_ and then filtered. The product is purified by column chromatography (pentane/TBME 30 : 1) and obtained as a mixture of rotamers in 66 % yield (998 mg, 2.68 mmol) together with the pyrrolidine isomer in 19 % yield (279 mg, 0.75 mmol). Piperidine‐Isomer: ^1^H NMR (400.1 MHz, CDCl_3_): δ [ppm]=7.42–7.35 (m, 2H,), 7.34–7.23 (m, 4H), 7.23–7.10 (m, 4H), 5.22–4.82 (m, 1H), 4.62–4.27 (m, 1H), 3.97–3.60 (m, 1H), 3.19–3.12 (m, 1H),3.11–3.00 (m, 1H), 2.92–2.70 (m, 2H), 2.49–2.28 (m, 1H),1.49 (s, 9H). ^13^C NMR (100.6 MHz, CDCl_3_): δ [ppm]=153.98, 146.01, 128.61, 127.61, 126.29, 80.64, 52.06, 51.41, 50.94, 48.79, 45.93, 28.38. HRMS (ESI): *m/z*=394.1548 [M+Na]^+^ (calculated for C_22_H_26_ClNO_2_+Na^+^: 394.1544). Pyrrolidine‐Isomer: ^1^H NMR (400.1 MHz, CDCl_3_): δ [ppm]=7.26–7.06 (m, 20H), 4.65 (dd, *J*=11.6, 2.2 Hz, 1H), 4.45 (dd, *J*=11.5, 2.1 Hz, 1H), 3.93–3.81 (m, 1H), 3.79–3.64 (m, 4H), 3.59–3.44 (m, 3H), 2.82–2.70 (m, 2H), 2.67–2.57 (m, 2H), 1.42 (s, 9H), 1.38 (s, 9H). ^13^C NMR (100.6 MHz, CDCl_3_): δ [ppm]=δ 154.65, 145.26, 128.71, 126.76, 126.59, 80.50, 57.11, 56.07, 52.99, 46.85, 42.81, 28.54.

### 3‐Chloro‐5,5‐diphenylpiperidine hydrochloride (13 c)

372 mg (1 mmol, 1 equiv.) *N*‐Boc‐3‐chloro‐5,5‐diphenylpiperidine are dissolved in 5 mL dioxane. 2.5 mL 4 n HCl⋅dioxane are added and the solution is stirred for 16 h at room temperature. 5 mL of diethyl ether are added and the suspension is centrifuged. The resulting white solid is washed two times with diethyl ether and dried in vacuo. The product is obtained in 96 % yield (296 mg, 0.96 mmol). ^1^H NMR (400.1 MHz, DMSO‐d_6_): δ [ppm]=7.62–7.56 (m, 2H), 7.46–7.39 (m, 2H), 7.33–7.26 (m, 3H), 7.24–7.16 (m, 3H), 4.45 (d, *J*=13.6 Hz, 1H), 4.08–3.98 (m, 1H), 3.49 (d, *J*=13.4 Hz, 1H), 3.46–3.39 (m, 1H), 3.39–3.36 (m, 1H), 3.23 (t, *J*=11.6 Hz, 1H), 2.53 (d, *J*=12.8 Hz, 1H).^13^C NMR (100.6 MHz, DMSO‐d_6_): δ [ppm]=145.21, 140.14, 129.35, 128.54, 127.15, 126.82, 125.78, 49.84, 48.28, 46.57, 41.62.HRMS (ESI): *m/z=*272.1199 [M–Cl^−^]^+^ (calculated for C_17_H_18_ClN+H^+^: 272.1201).

### 5,5‐Diphenyl‐1‐azabicycle[3.1.0]hexane (11 c)

Under nitrogen atmosphere, 2.00 g (6.49 mmol, 1 equiv.) of 3‐chloro‐5,5‐diphenylpiperidine hydrochloride are dissolved in 20 mL of THF. The solution is cooled down to −78 °C. 3 equiv. of *n*‐BuLi (7.80 mL of a 2.5 m solution, 19.46 mmol) are added carefully. The reaction mixture is stirred at −78 °C for 2 h and then quenched with 15 mL of NH_4_Cl. Diethyl ether is added and the phases are separated. The organic phase is separated, dried over MgSO_4_ and evaporated. The product is obtained in 85 % yield (1.302 g, 5.53 mmol). ^1^H NMR (400.1 MHz, CDCl_3_): δ [ppm]=7.32–7.22 (m, 9H), 7.21–7.10 (m, 3H), 3.96 (d, *J*=12.2 Hz, 1H), 3.48 (d, *J*=12.3 Hz, 1H), 2.94 (dd, *J*=13.4, 2.5 Hz, 1H), 2.77–2.67 (m, 1H), 2.59–2.50 (m, 1H), 1.88 (d, *J*=1.2 Hz, 2H), 1.45 (d, *J*=3.4 Hz, 1H). ^13^C NMR (100.6 MHz, CDCl_3_): δ [ppm]=δ 149.35, 147.50, 128.66, 128.36, 126.83, 126.76, 126.15, 125.81, 66.09, 59.99, 40.30, 39.99, 25.64. HRMS (ESI): *m/z*=236.1432 [M+H]^+^ (calculated for C_17_H_17_N+H^+^: 236.1434).

### 2,2‐Diethylpen‐4‐enal

To a solution of 5.00 g (50 mmol, 1 equiv.) ethylbutyraldehyde in 220 mL DCM 7.31 g (65 mmol, 1.3 equiv.), potassium *tert*‐butoxide are added followed by 8.7 mL (100 mmol, 2 equiv.) of allyl bromide. The mixture is stirred for 2 h at room temperature. 100 mL water is added, the phases are separated and the organic phase is washed with 100 mL of water, dried over MgSO_4_ and the solvent is removed under reduced pressure. After distillation (102–104 °C, 100 mbar), the product is obtained as a colourless oil in 60 % yield (4.20 g, 29.95 mmol). ^1^H NMR (400.1 MHz, CDCl_3_): δ [ppm]=5.73–5.47 (m, 1H), 5.14–4.98 (m, 2H), 2.19 (dt, *J*=7.4, 1.3 Hz, 2H), 1.48 (q, *J*=7.6 Hz, 4H), 0.74 (t, *J*=7.5 Hz, 6H). ^13^C NMR (100.6 MHz, CDCl_3_): δ [ppm]=206.88, 133.12, 118.05, 52.49, 35.06, 24.44, 7.87.

### 2,2‐Diethylpent‐4‐enal oxime

2.495 g (35.91 mmol, 1.2 equiv.) hydroxylammonium chloride in 2.5 mL H_2_O are added to a solution of 1.177 g (29.92 mmol, 1 equiv.) NaOH in 2.5 mL H_2_O. After 5 min, 4.196 g 2,2‐diethylpen‐4‐enal in 70 mL ethanol are added. The solution is left standing for 16 h and the ethanol is removed under reduced pressure. 50 mL of H_2_O and 150 mL of TBME are added and the phases are separated. The aqueous phase is extracted with 3×50 mL of TBME. The combined organic phases are dried over Na_2_SO_4_ and the solvent is removed under reduced pressure. The product is obtained as a colourless oil in 94 % yield (4.381 g, 28.22 mmol). ^1^H NMR (400.1 MHz, CDCl_3_): δ [ppm]=7.23 (s, 1H), 5.72 (ddt, *J*=16.5, 11.0, 7.4 Hz, 1H), 5.12–5.01 (m, 2H), 2.20 (dt, *J*=7.3, 1.3 Hz, 2H), 1.46 (qd, *J*=7.4, 1.7 Hz, 4H), 0.82 (t, *J*=7.5 Hz, 6H). ^13^C NMR (100.6 MHz, CDCl_3_): δ [ppm]=157.81, 133.87, 117.87, 43.01, 38.44, 27.55, 7.94. HRMS (ESI): *m/z*=178.1203 [M+Na]^+^ (calculated for C_9_H_17_NO+Na^+^: 178.1202).

### 2,2‐Diethylpent‐4‐en‐1‐amine (9 d)

1.246 g (21.84 g, 1.5 equiv.) LAH are suspended in 50 mL dry diethyl ether. At 0 °C, 3.399 g (21.84 mmol, 1 equiv.) 2,2‐diethylpent‐4‐enal oxime in 20 mL dry diethyl ether are added dropwise. The suspension is stirred for 16 h at room temperature. The reaction is quenched by the subsequent addition of 1.25 mL H_2_O, 1.25 mL 20 % NaOH_aq_ and 3.75 mL H_2_O. The mixture is dried over Na_2_SO_4_ and filtered through a Büchner funnel. After distillation (92–96 °C, 50 mbar), the product is obtained as a colourless oil in 63 % yield (1.945 g, 13.77 mmol). ^1^H NMR (600.1 MHz, CDCl_3_): δ [ppm]=5.89–5.61 (m, 1H), 5.08–4.93 (m, 2H), 2.44 (s, 2H), 1.94 (d, *J*=7.4 Hz, 2H), 1.21 (q, *J*=7.5 Hz, 5H), 0.77 (t, *J*=7.5 Hz, 6H). ^13^C NMR (150.9 MHz, CDCl_3_): δ [ppm]=135.13, 116.88, 46.46, 39.69, 38.50, 26.02, 7.47. HRMS (ESI): *m/z*=142.1594 [M+H]^+^ (calculated for C_9_H_19_N+H^+^: 142.1590).

### N‐Chloro‐2,2‐diethylpent‐4‐en‐1‐amine (8 d)

In 20 mL dry DCM, 500 mg (3.54 mmol, 1 equiv.) 2,2‐diethylpent‐4‐en‐1‐amine are dissolved. At −15 °C 473 mg NCS (3.54 mmol, 1 equiv.) are added and the solution is stirred at −15 °C for 4 h. After TLC showed completion of the reaction, the solvent is removed under reduced pressure. 20 mL pentane are added and the precipitating succinic imide is filtered of. The solvent is removed under reduced pressure and the product is obtained as a pale blue oil in 88 % yield (549 mg, 3.12 mmol). ^1^H NMR (400.1 MHz, CDCl_3_): δ [ppm]=5.89–5.67 (m, 1H), 5.19–4.95 (m, 2H), 2.90 (s, 2H), 2.01 (d, *J*=7.5 Hz, 2H), 1.30 (q, *J*=7.5 Hz, 4H), 0.82 (t, *J*=7.5 Hz, 6H). ^13^C NMR (150.9 MHz, CDCl_3_): δ [ppm]=134.60, 117.76, 62.28, 40.91, 39.24, 26.79, 7.54. HRMS (ESI): *m/z*=176.1220 [M+H]^+^ (calculated for C_9_H_18_ClN+H^+^: 176.1201).

### N‐Boc‐3‐chloro‐5,5‐diethylpiperidine (12 d)

In 50 mL dry CHCl_3_, 1.116 g (6.35 mmol, 1 equiv.) *N*‐chloro‐2,2‐diethylpent‐4‐en‐1‐amine are dissolved and 235 mg (0.64 mmol, 0.1 equiv.) TBAI are added at room temperature. The solution is heated to 60 °C for 3 h. 1.2 equiv. of di‐*tert*‐butyldicarbonate (1.66 g, 7.62 mmol) are added at room temperature and the mixture is stirred for 16 h. 20 mL of water are added and the aqueous phase is extracted with DCM (3×20 mL) The organic phase is dried with MgSO_4_ and then filtered. The product is purified by column chromatography (pentane/TBME 50 : 1) and obtained as a mixture of rotamers in 70 % yield (1.23 g, 4.46 mmol). ^1^H NMR (400.1 MHz, CDCl_3_): δ [ppm]=4.59–4.26 (m, 1H), 4.06–3.74 (m, 2H), 2.78–2.27 (m, 2H), 2.22–2.07 (m, 1H), 1.45 (s, 9H), 1.40–1.17 (m, 5H), 0.85–0.77 (m, 6H). ^13^C NMR (100.6 MHz, CDCl_3_): δ [ppm]=154.65, 52.18, 51.45, 51.07, 45.20, 29.29, 28.49, 23.65, 7.73, 7.15. HRMS (ESI): *m/z*=298.1548 [M+Na]^+^ (calculated for C_14_H_26_ClNO_2_+Na^+^: 298.1544).

### 3‐Chloro‐5,5‐diethylpiperidine hydrochloride (13 d)

500 mg (1 mmol, 1 equiv.) *N*‐Boc‐3‐chloro‐5,5‐diethylpiperidine are dissolved in 4.5 mL 4 n HCl⋅dioxane and the solution is stirred for 16 h at room temperature. 5 mL of diethyl ether are added and the suspension is centrifuged. The resulting white solid is washed two times with diethyl ether and dried in vacuo. The product is obtained in 89 % yield (335 mg, 1.59 mmol). ^1^H NMR (400.1 MHz, CDCl_3_): δ [ppm]=10.28 (s, 1H), 9.43 (s, 1H), 4.32–4.20 (m, 1H), 3.77–3.65 (m, 1H), 3.23–3.08 (m, 1H), 2.84–2.69 (m, 1H), 2.65–2.52 (m, 1H), 2.26–2.16 (m, 1H), 1.71–1.61 (m, 2H), 1.45 (t, *J*=12.8 Hz, 1H), 1.39–1.30 (m, 2H), 0.89–0.81 (m, 6H). ^13^C NMR (100.6 MHz, CDCl_3_): δ [ppm]=50.11, 49.60, 48.11, 42.27, 37.23, 29.85, 24.14, 7.34, 6.97. HRMS (ESI): *m/z*=176.1201 [M−Cl^−^]^+^ (calculated for C_9_H_19_ClN‐Cl^−^: 176.1201).

## Conflict of interests

The authors declare no conflict of interest.

1

## Supporting information

As a service to our authors and readers, this journal provides supporting information supplied by the authors. Such materials are peer reviewed and may be re‐organized for online delivery, but are not copy‐edited or typeset. Technical support issues arising from supporting information (other than missing files) should be addressed to the authors.

Supporting Information

## Data Availability

The data that support the findings of this study are available in the supplementary material of this article.
