# Supplementary material for: Secondary 3‐Chloropiperidines: Powerful Alkylating Agents
Source: ChemistryOpen. 2023 Dec 13;13(6):e202300181. doi: 10.1002/open.202300181 (PMC11164021; doi:10.1002/open.202300181)

ChemistryOpen

Supporting Information

Secondary 3-Chloropiperidines: Powerful Alkylating Agents

Mats Georg, Lina Alexandra Laping, Veronica Billo, Barbara Gatto, Peter Friedhoff, and Richard Göttlich*

2,2-dimethylpen-4-enal

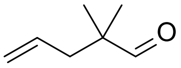

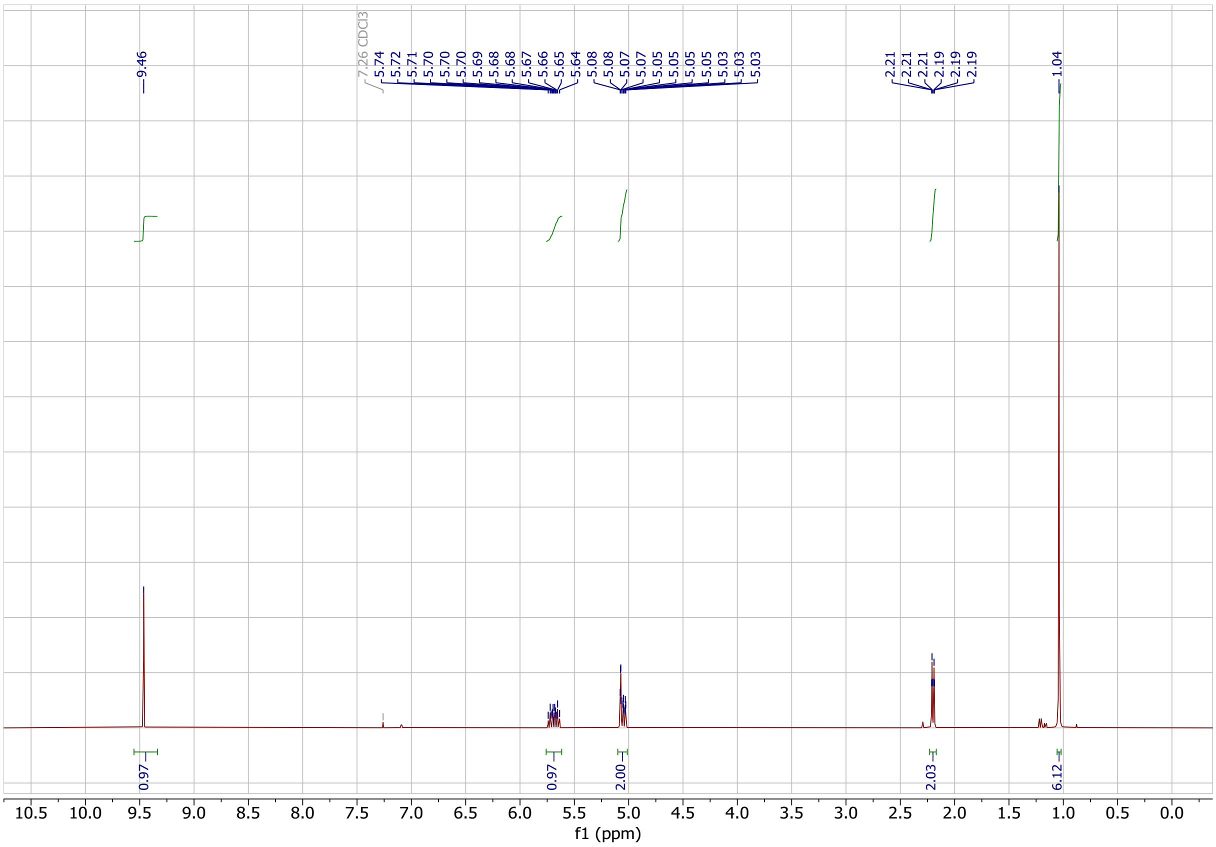

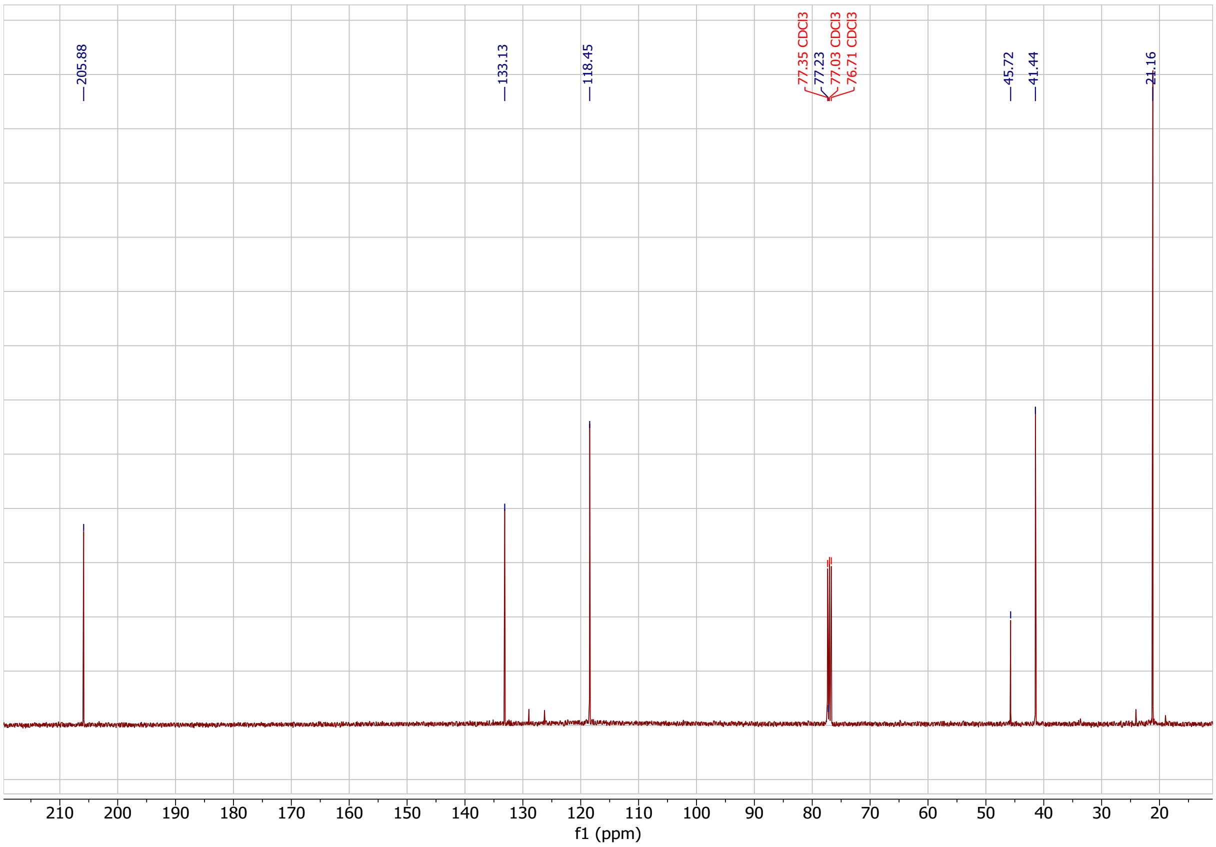

2,2-dimethylpent-4-enal oxime

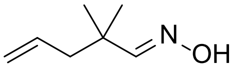

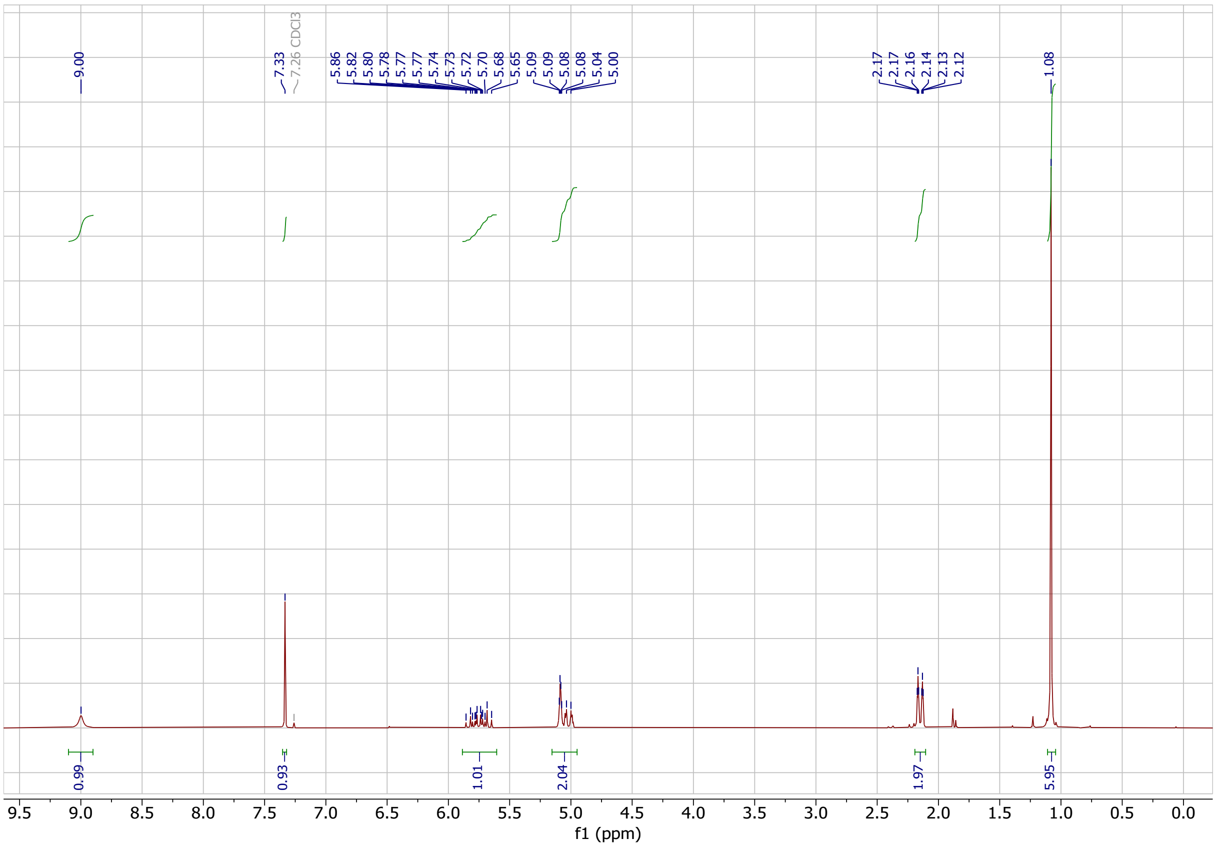

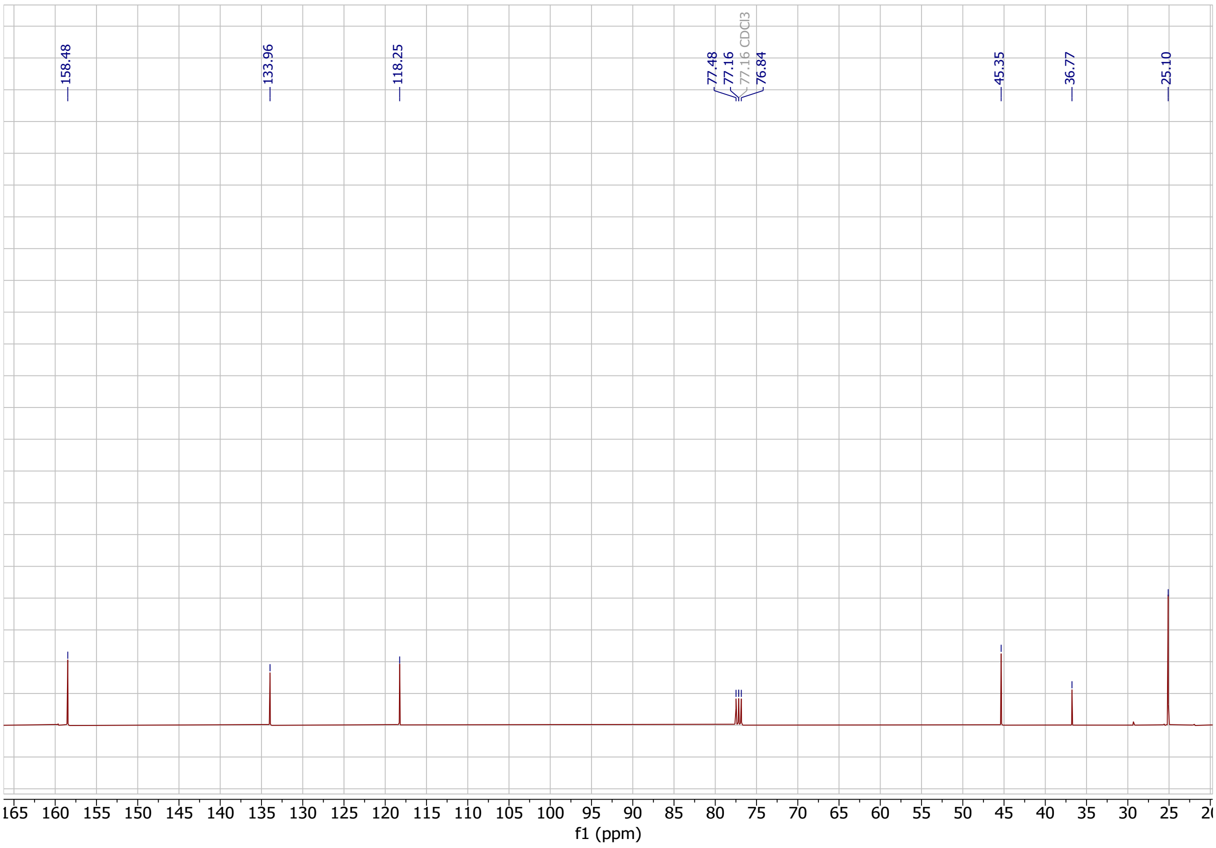

2,2-dimethylpent-4-en-1-amine (9a)

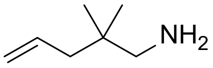

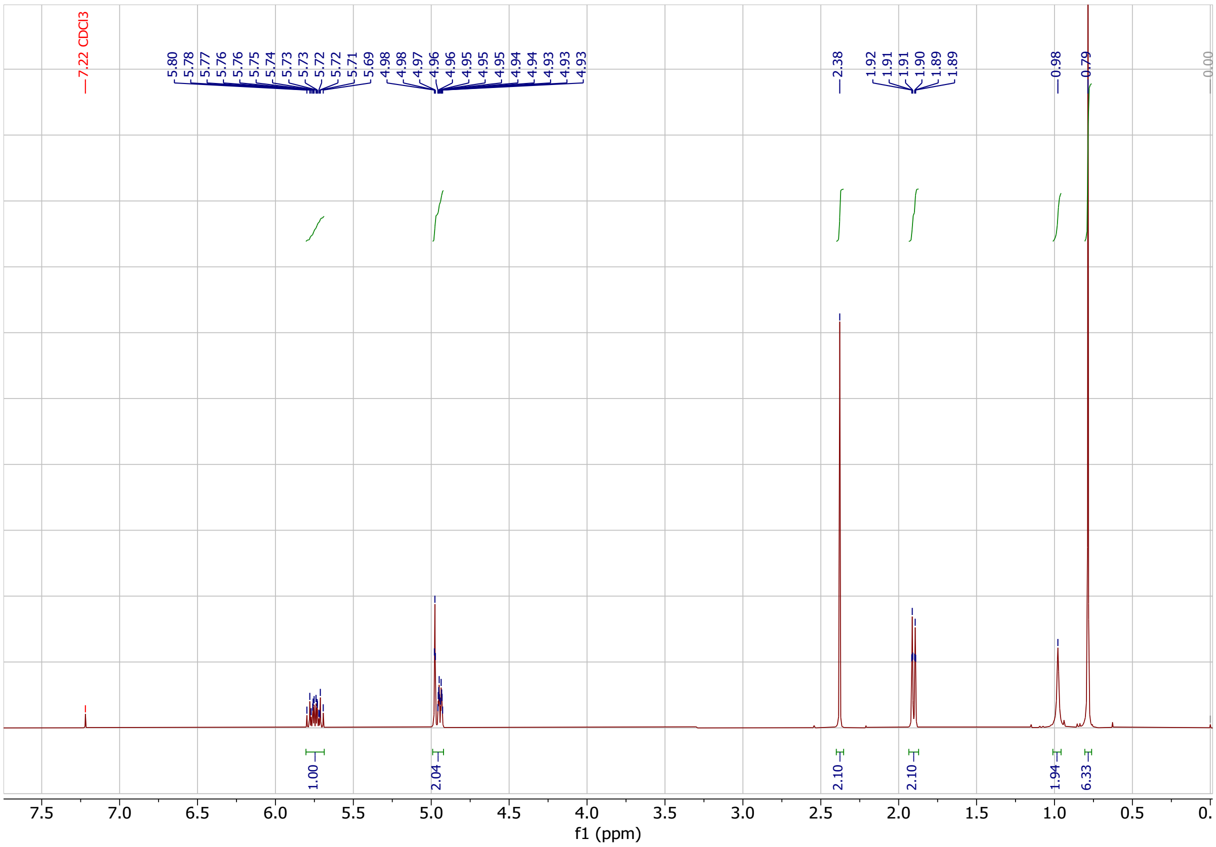

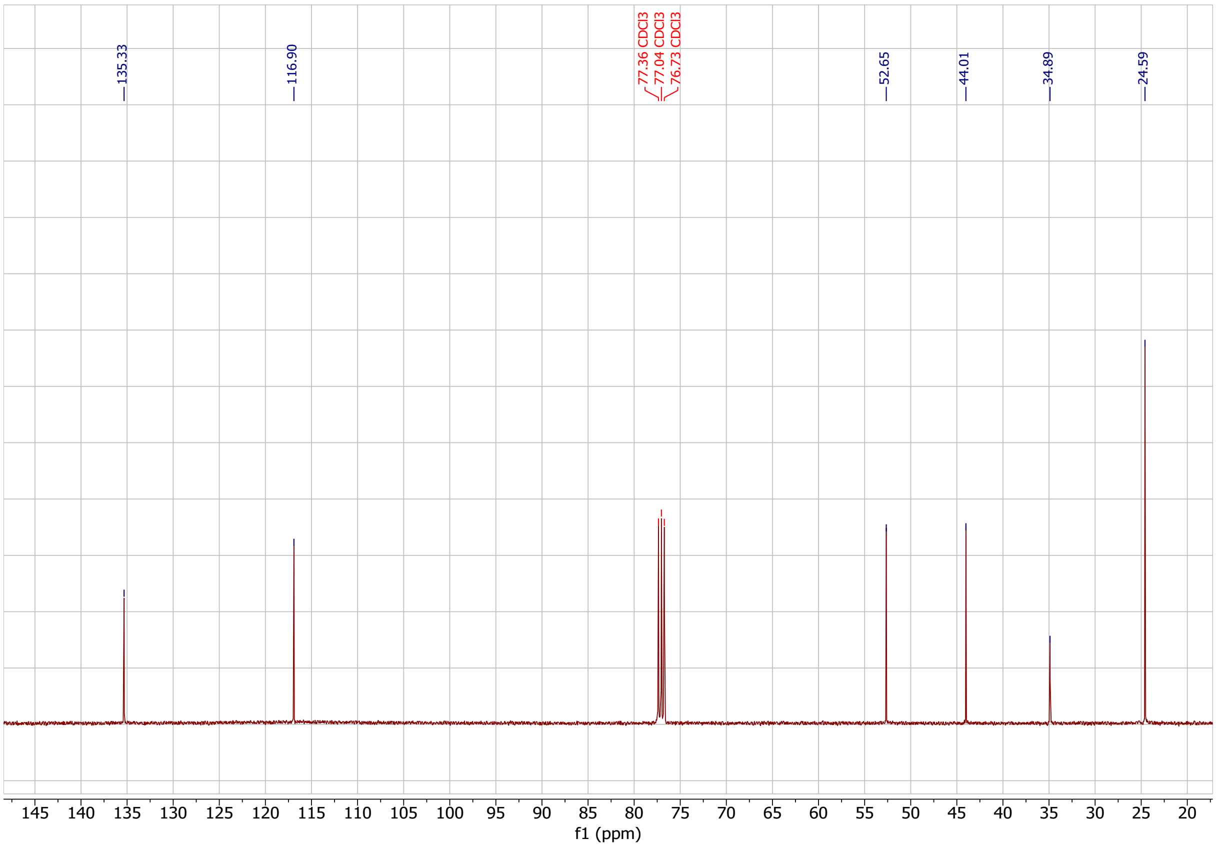

***N*-chloro-2,2-dimethylpent-4-en-1-amine (8a)**

***N*-chloro-2,2-dimethylpent-4-en-1-amine (8a)**

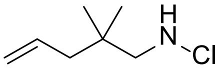

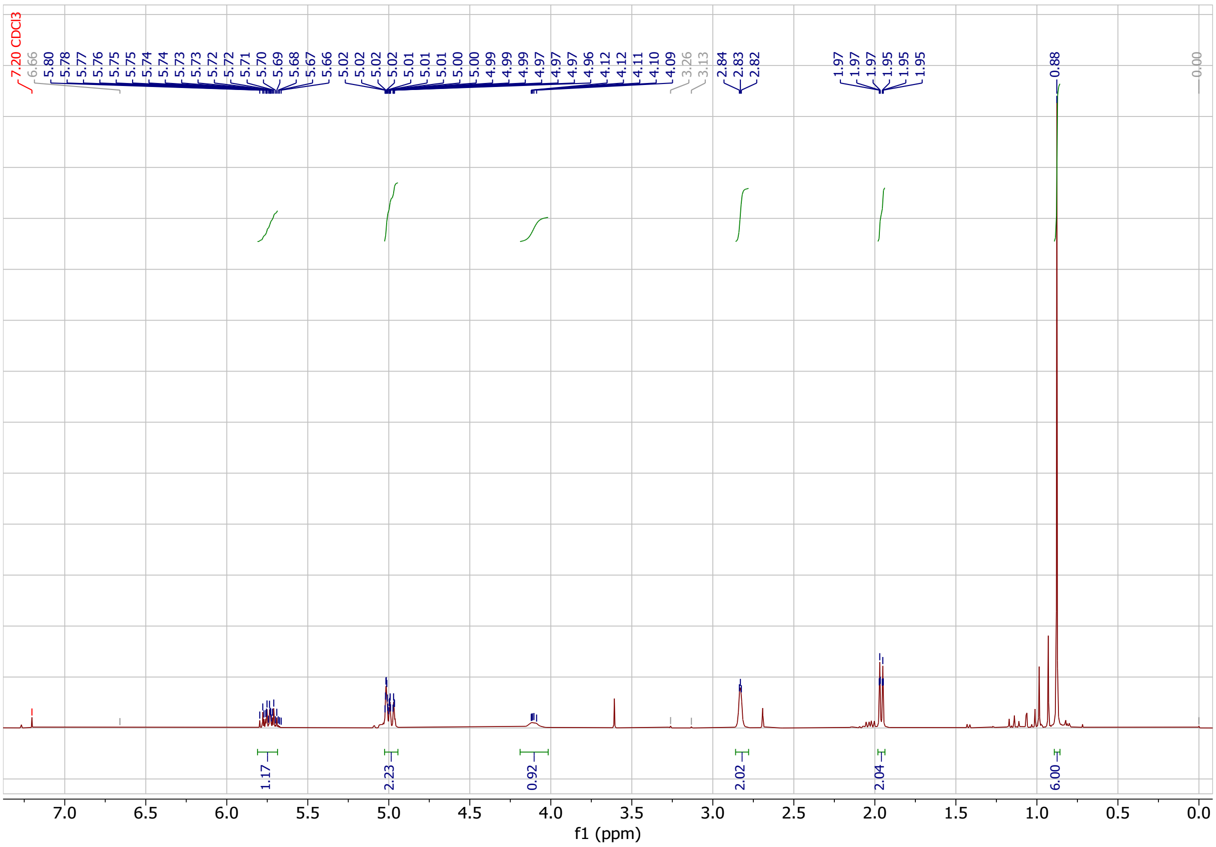

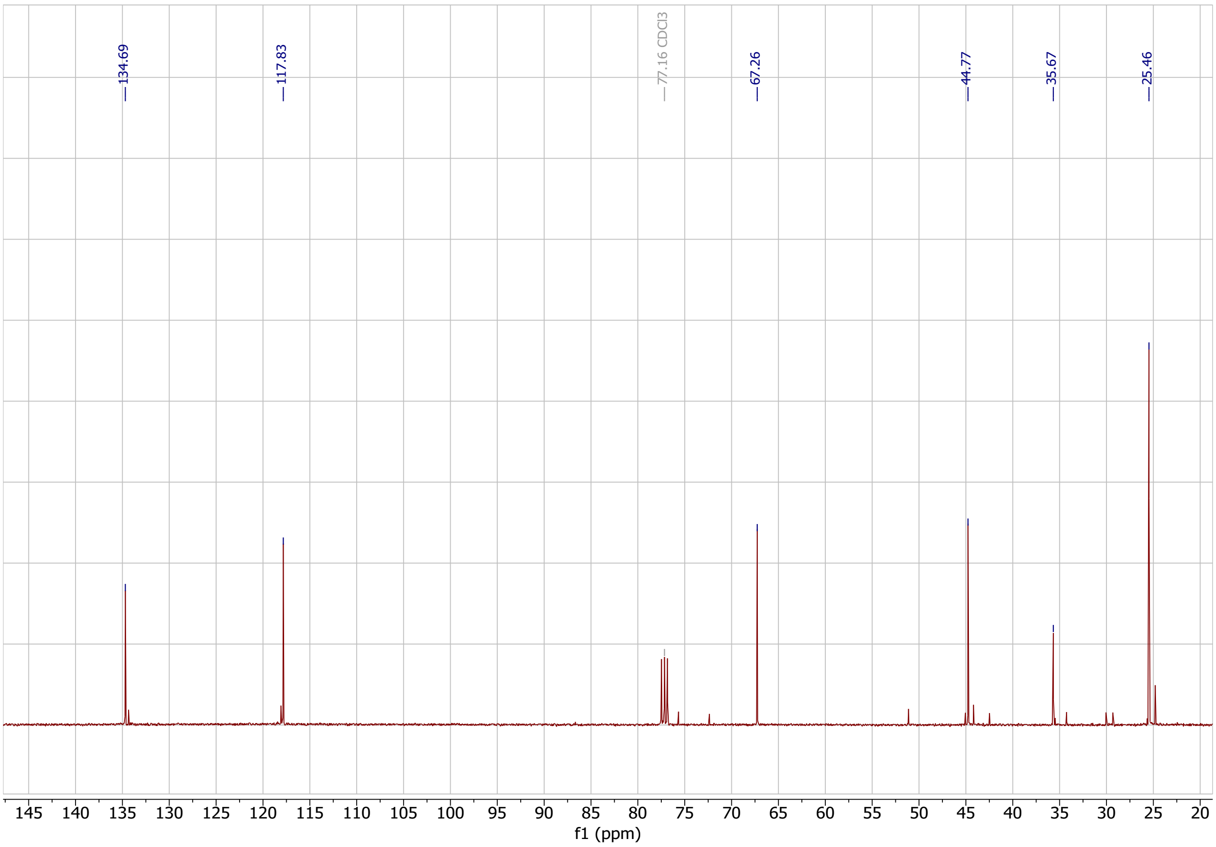

***N*-Boc-3-chloro-5,5-dimethylpiperidine (12a)**

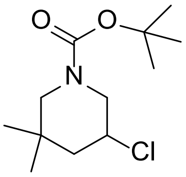

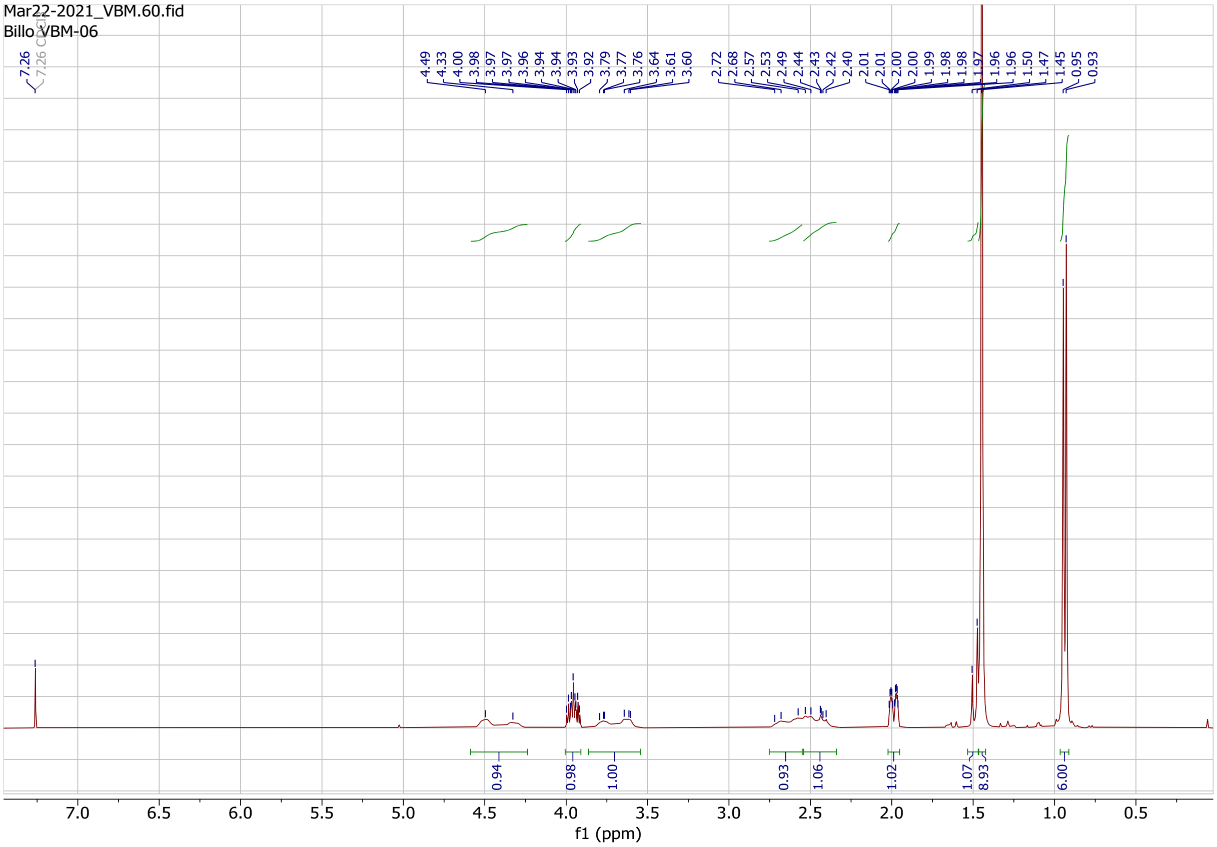

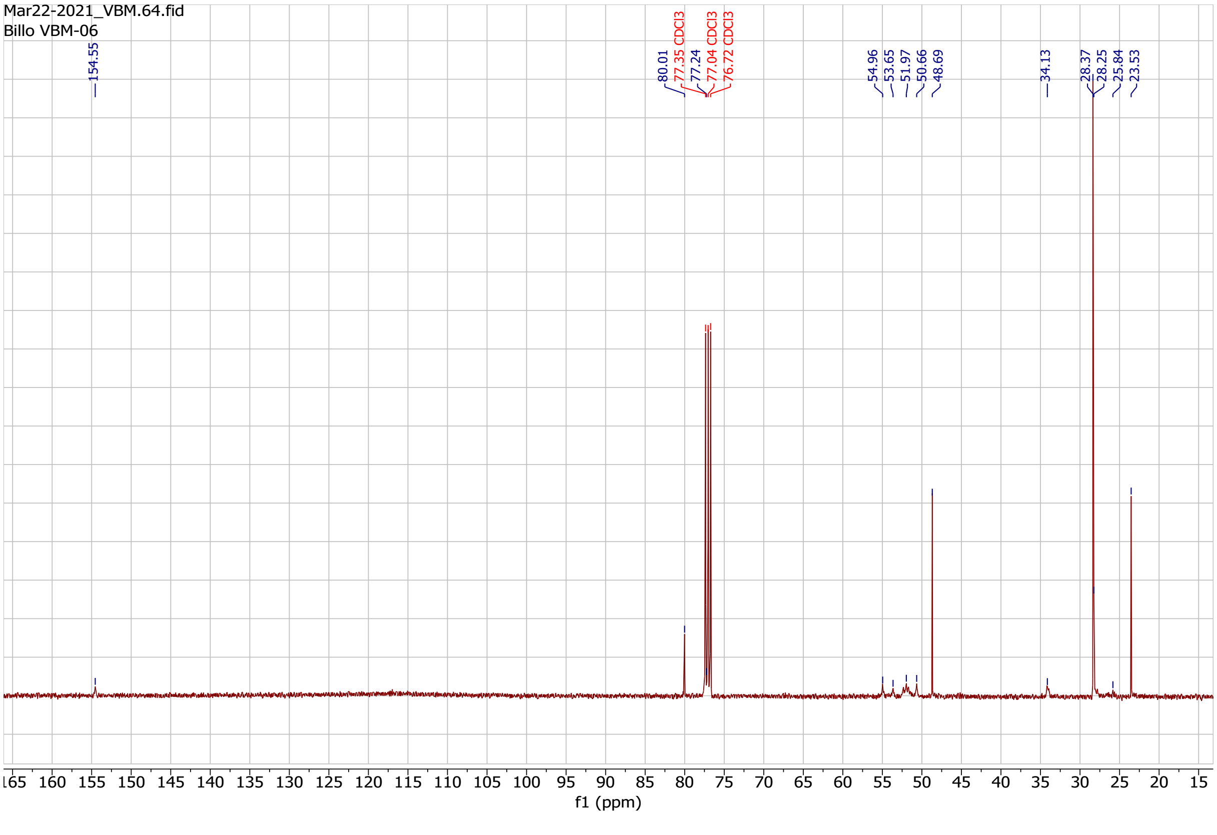

3-chloro-5,5-dimethylpiperidine hydrochloride (13a)

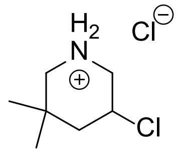

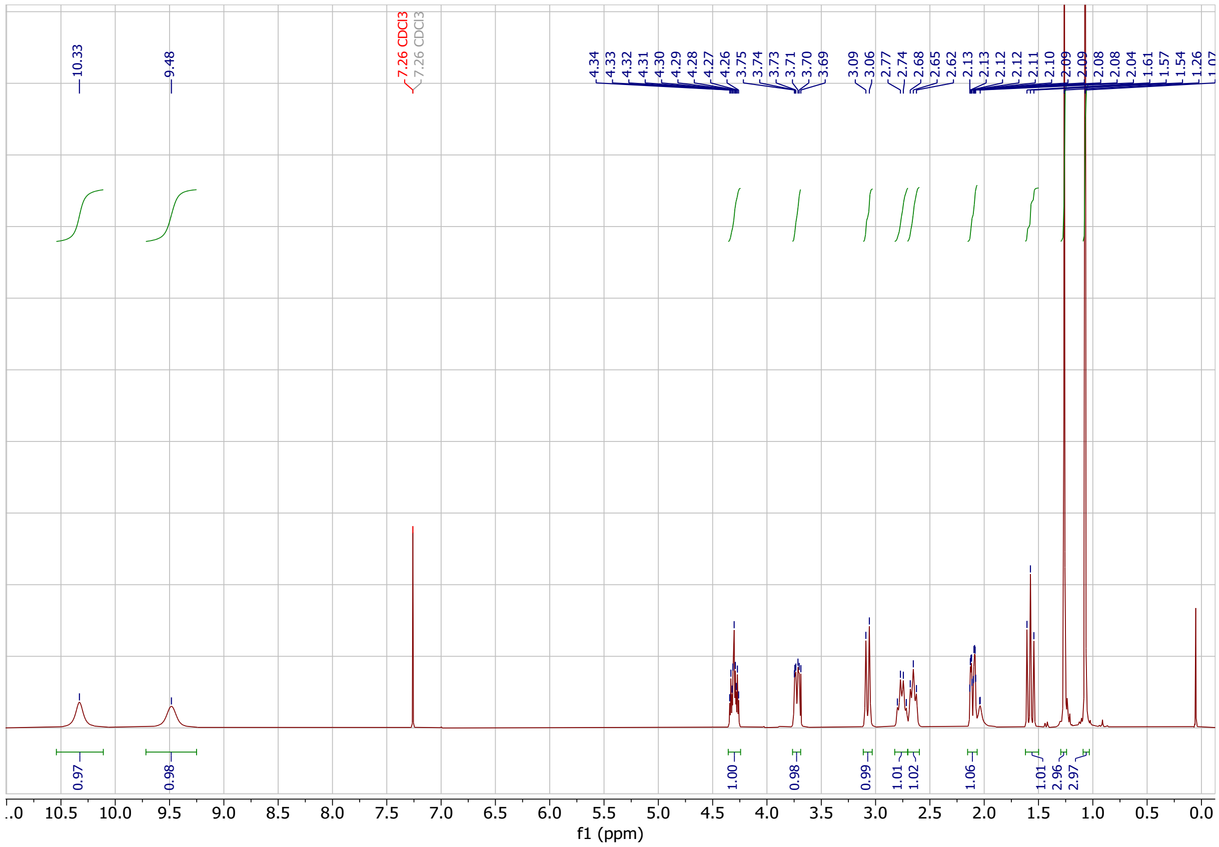

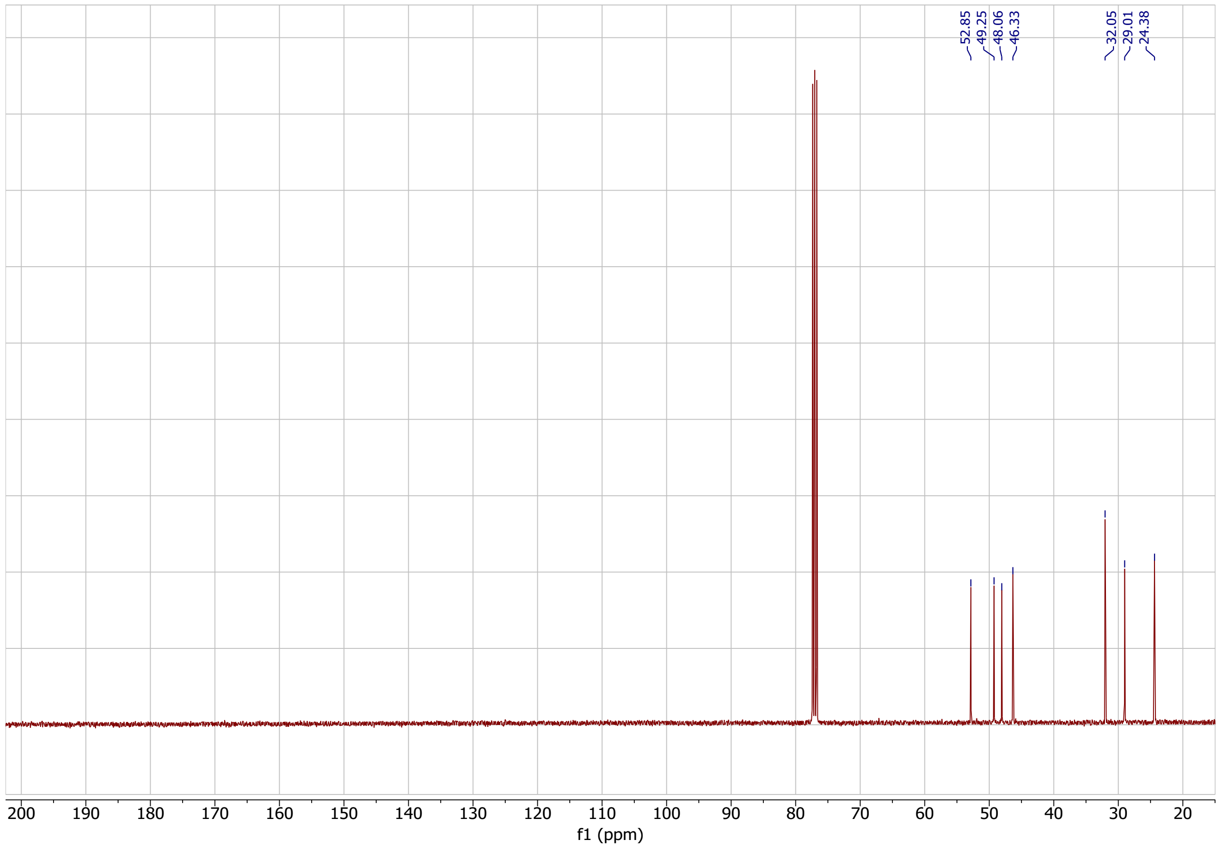

2-methyl-2-phenylpent-4-enal

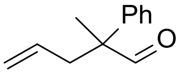

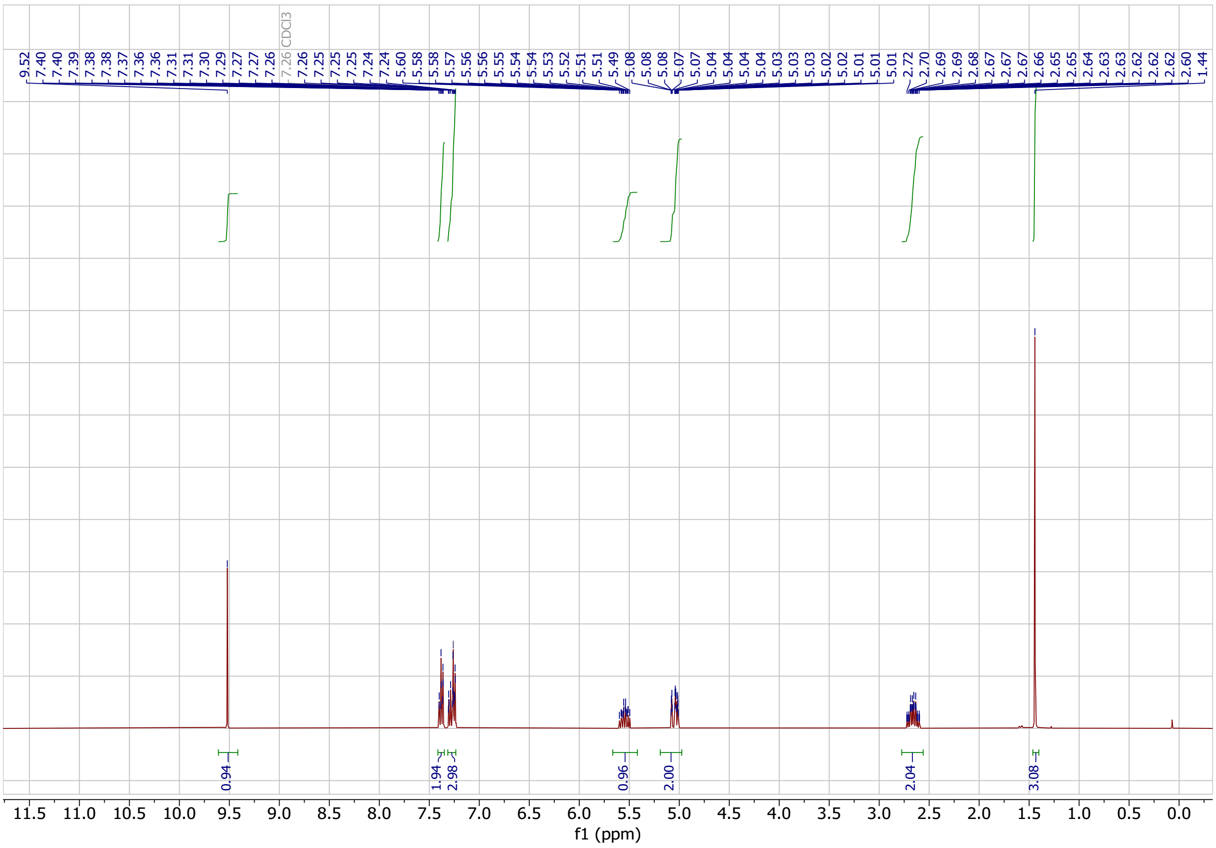

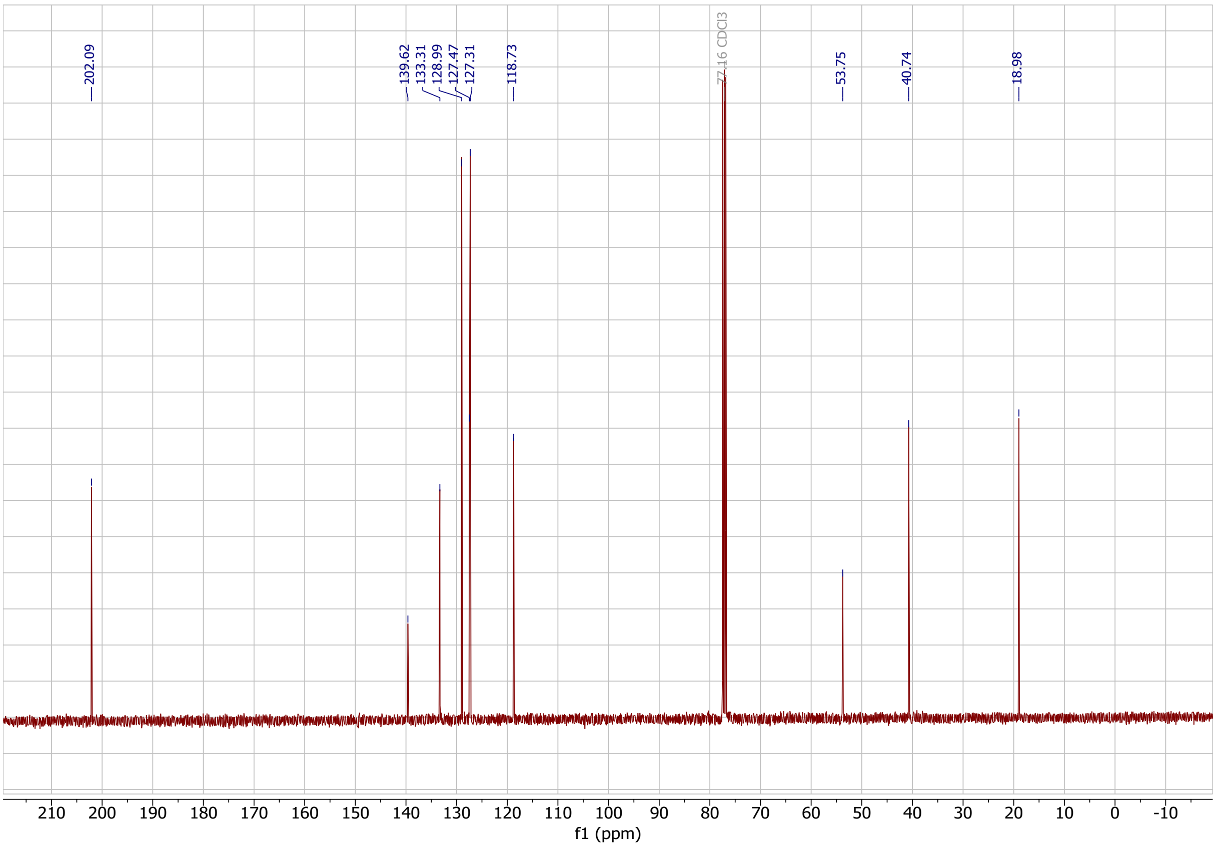

2-methyl-2-phenylpent-4-enal oxime

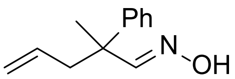

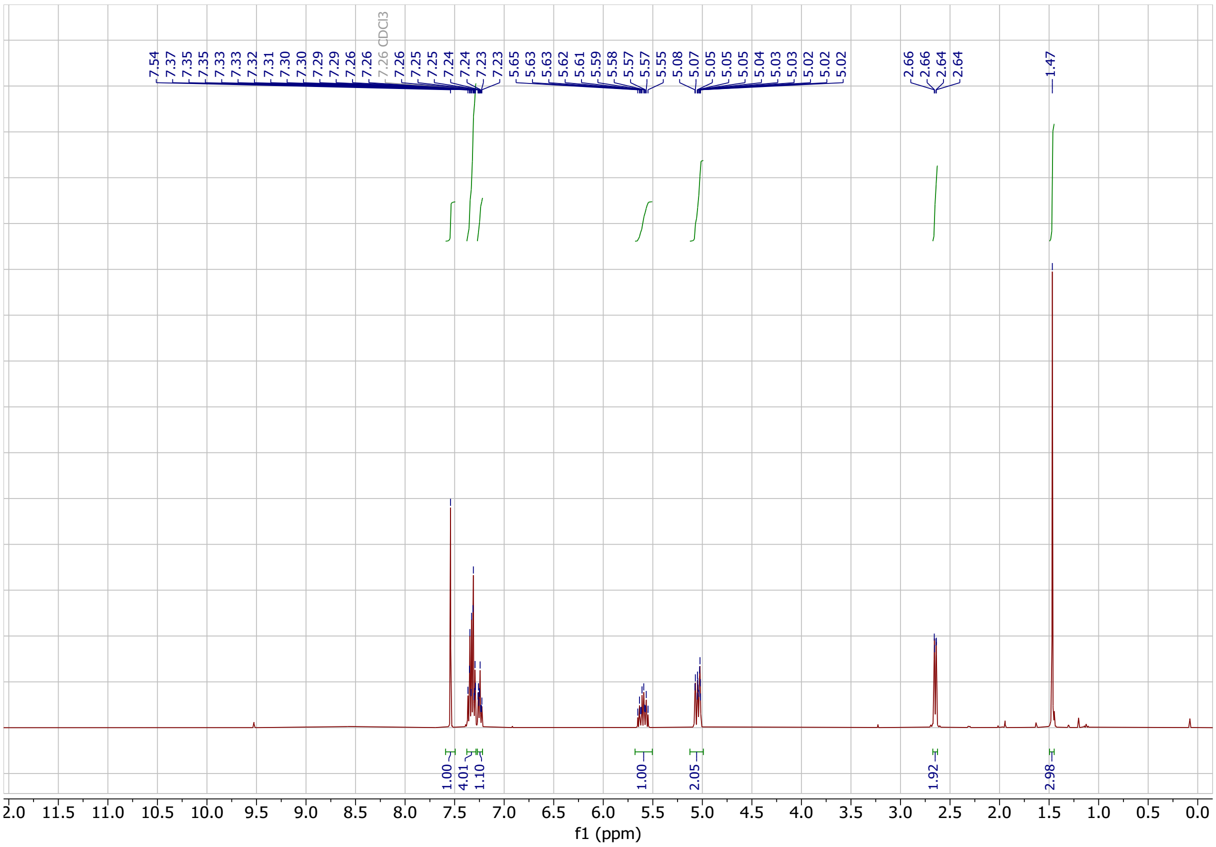

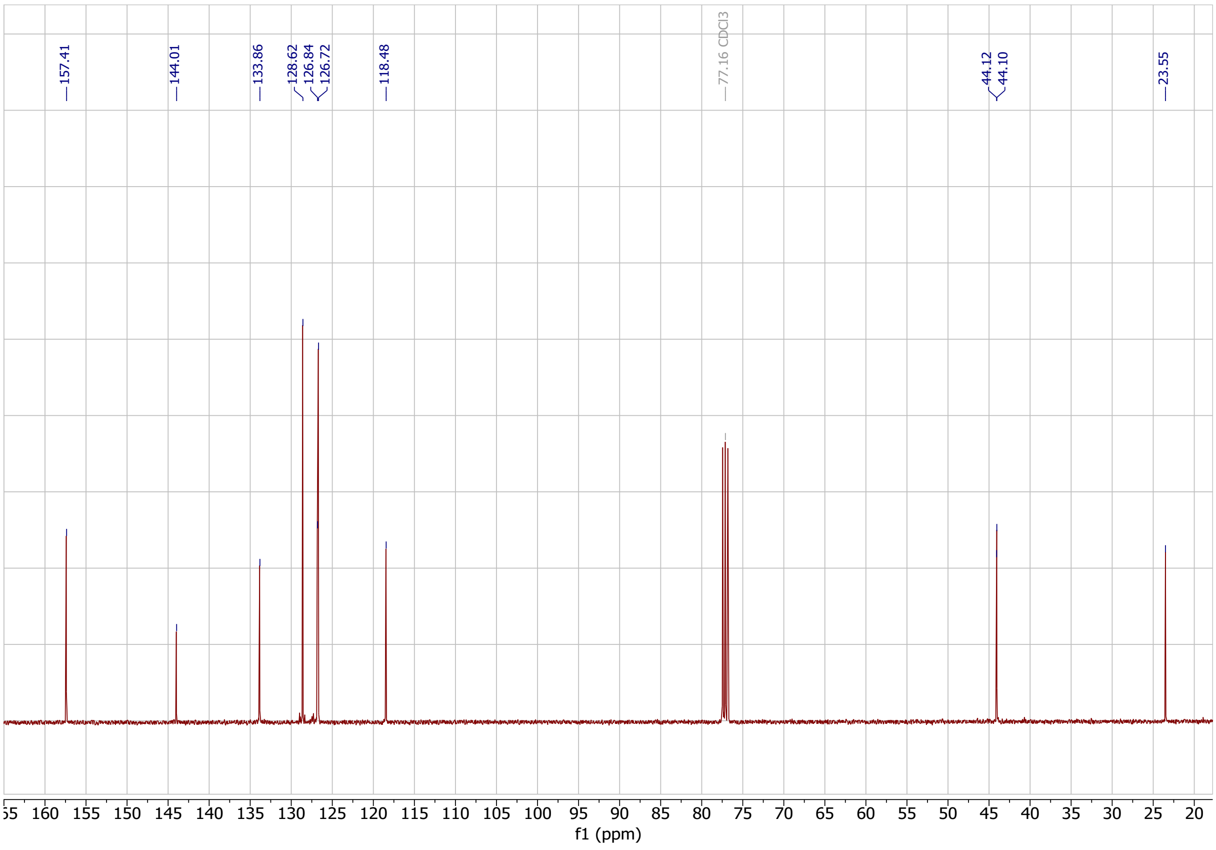

2-methyl-2-phenylpent-4-en-1-amine (9b)

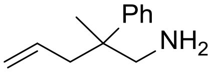

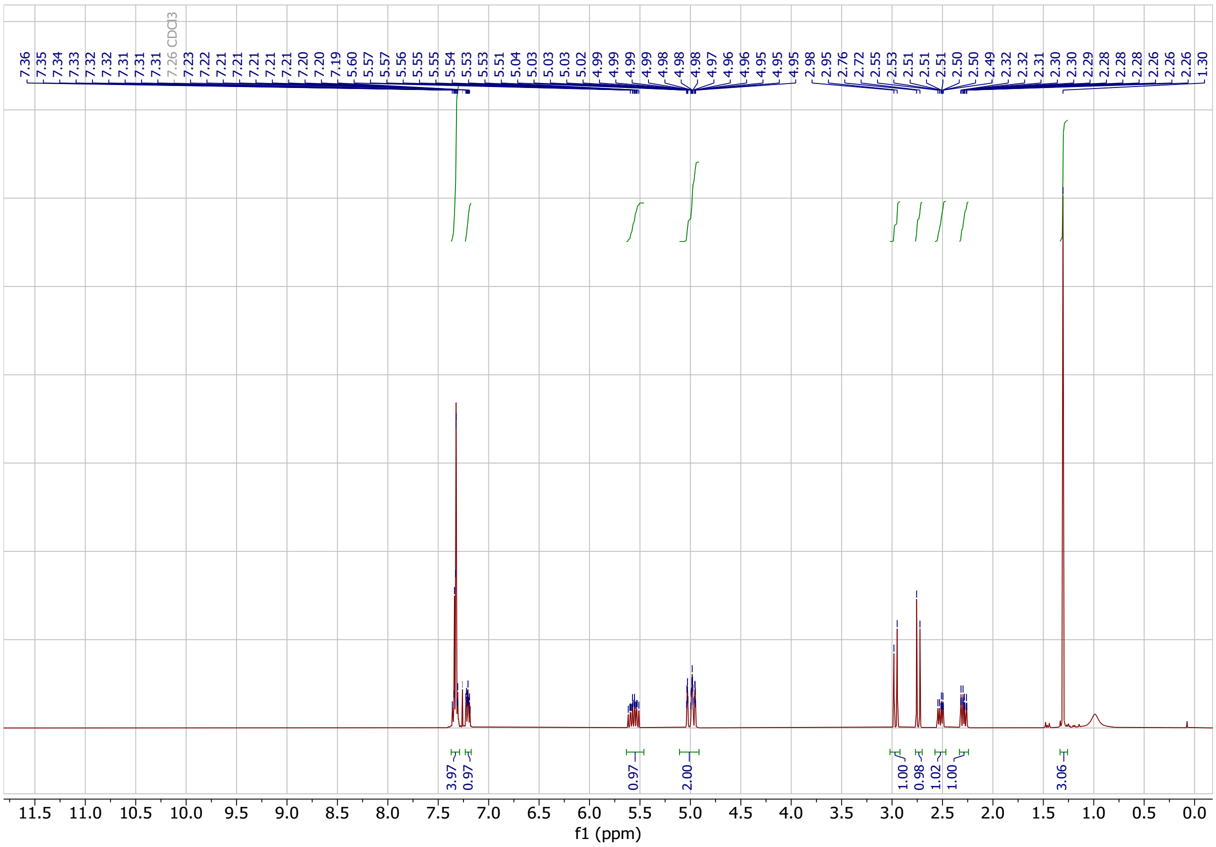

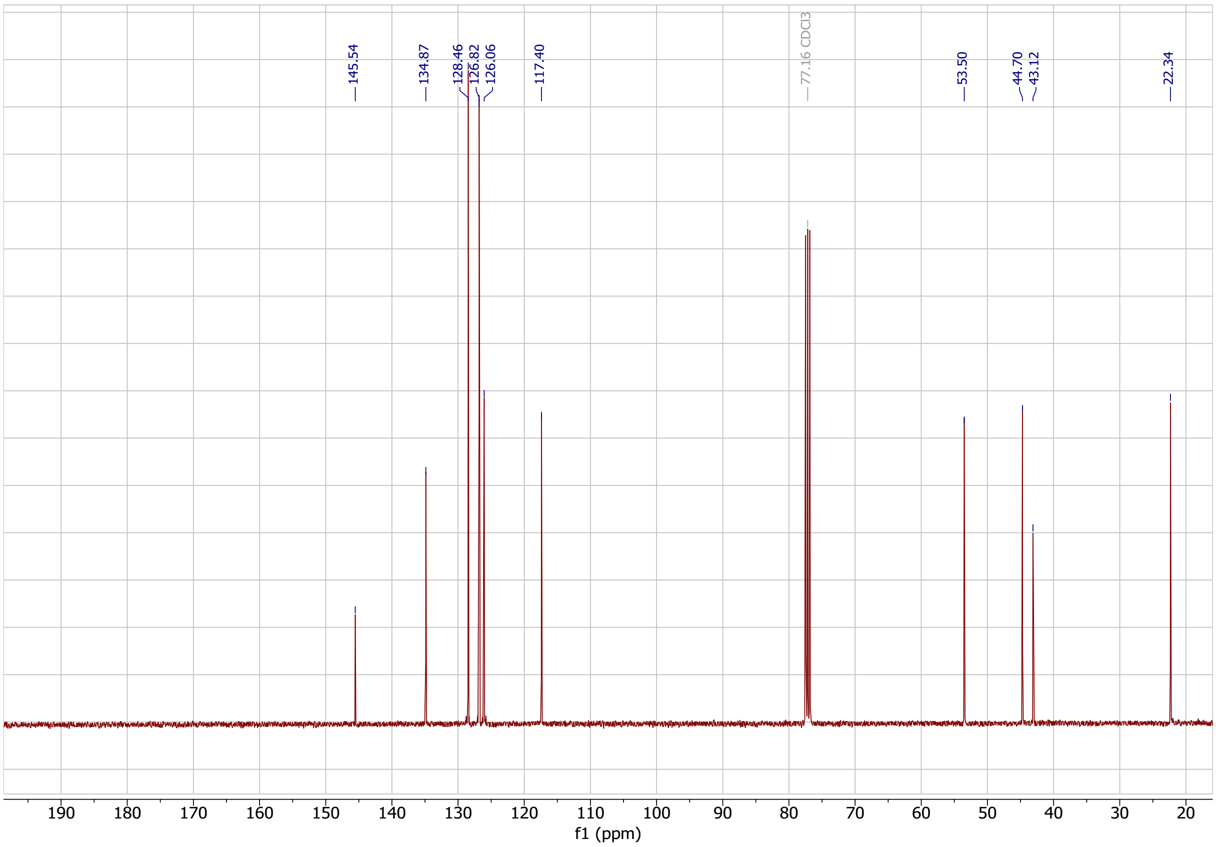

***N*-chloro-2-methyl-2-phenylpent-4-en-1-amine (8b)**

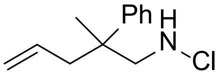

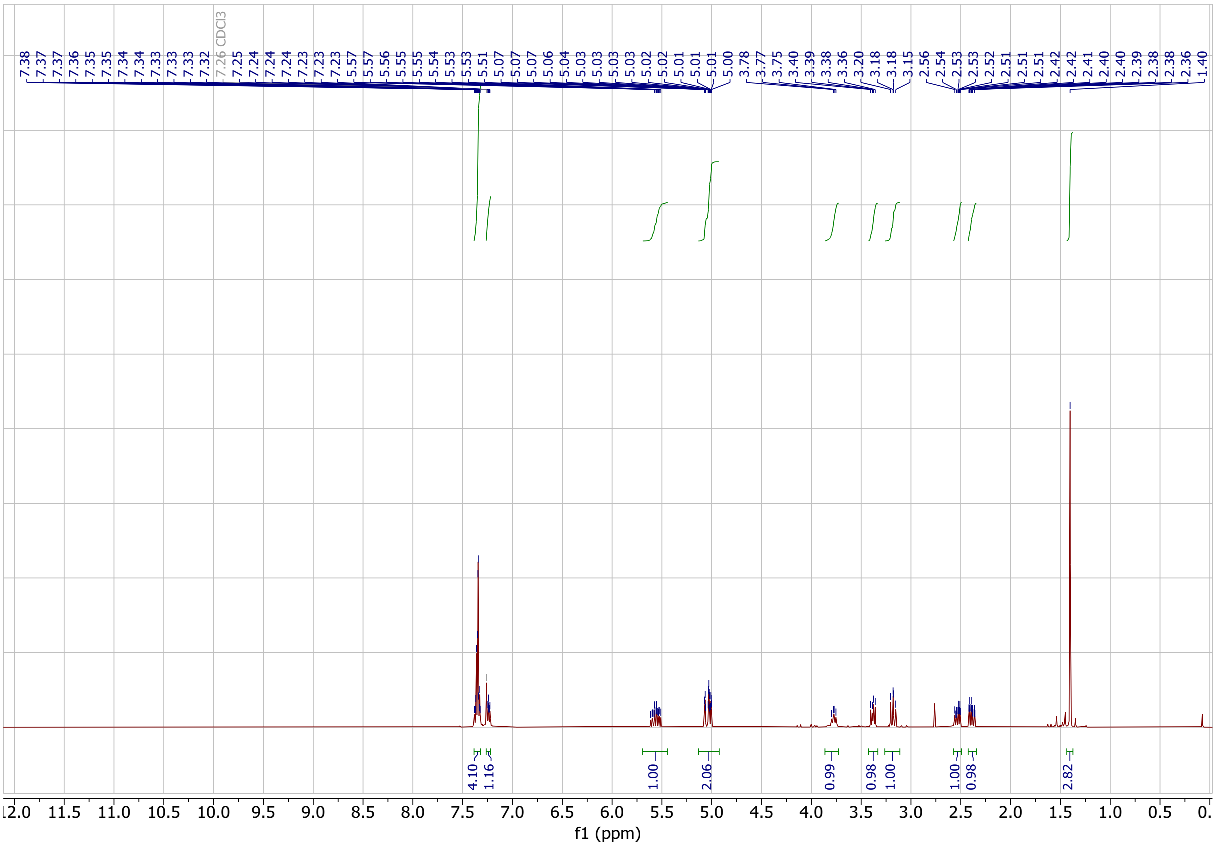

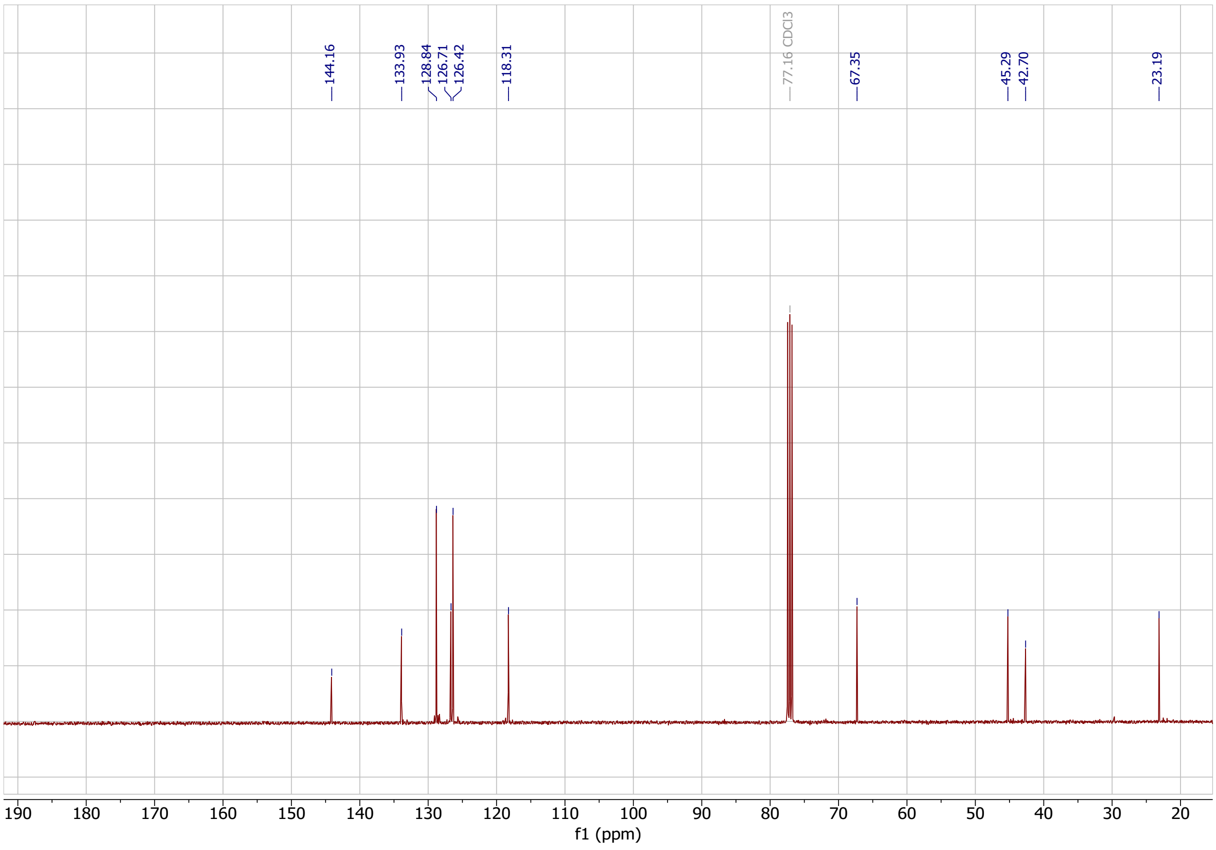

3-chloro-5-methyl-5-phenylpiperidine (7b)

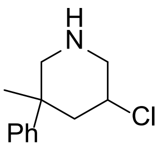

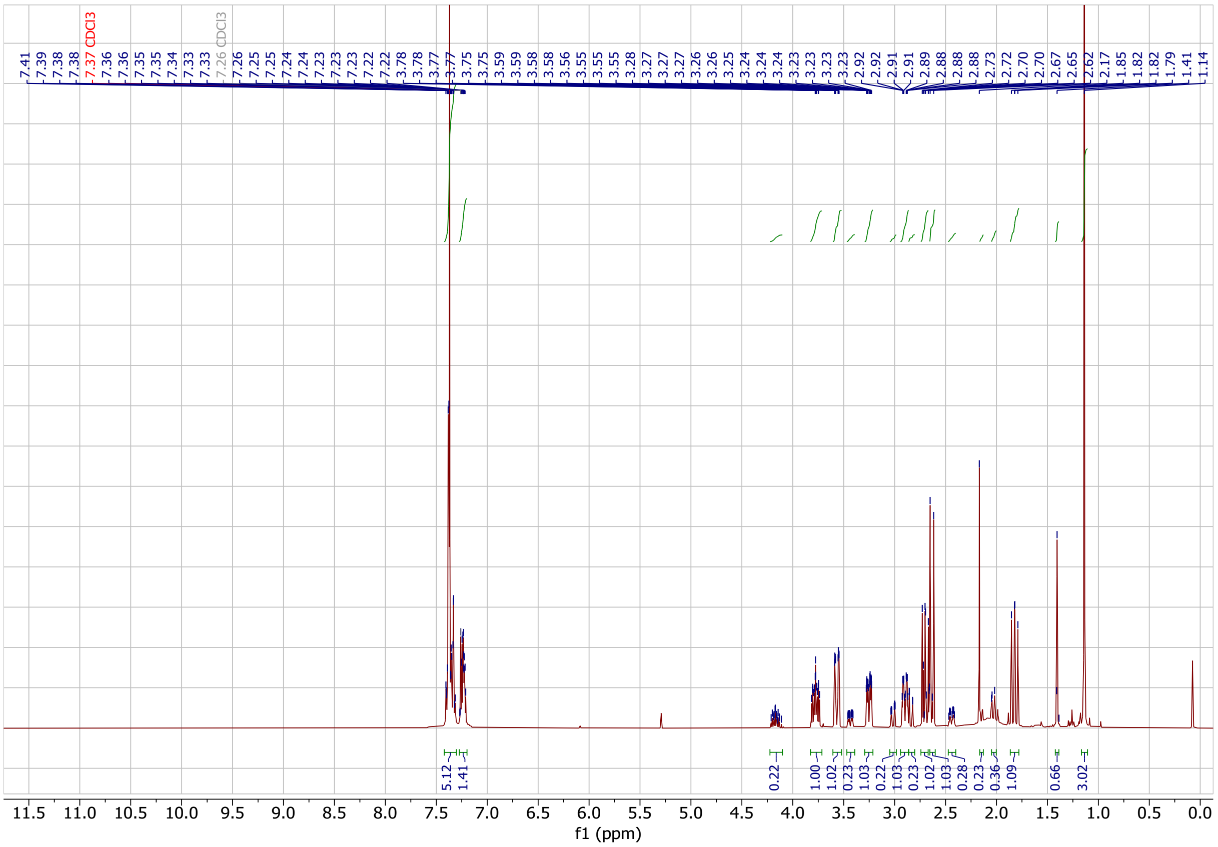

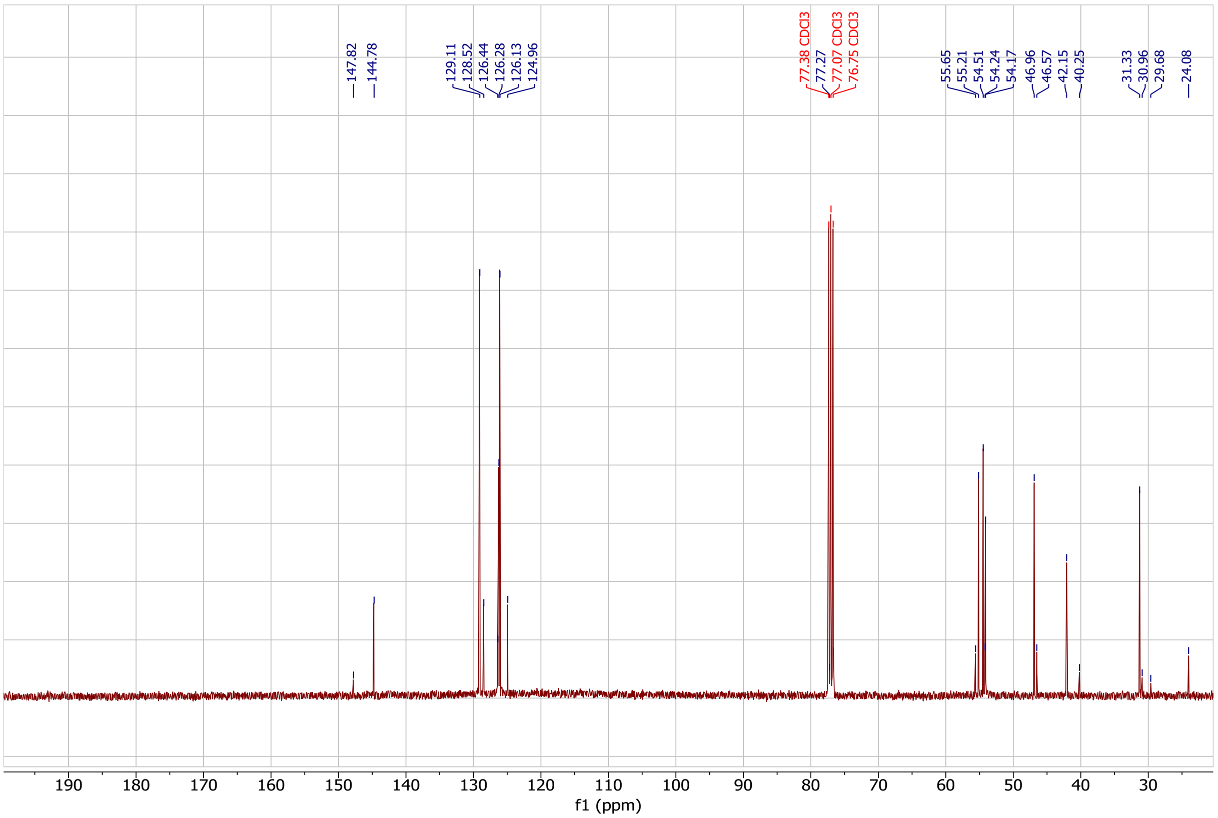

3-chloro-5-methyl-5-phenylpiperidine hydrochlorid (13b)

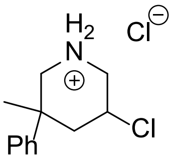

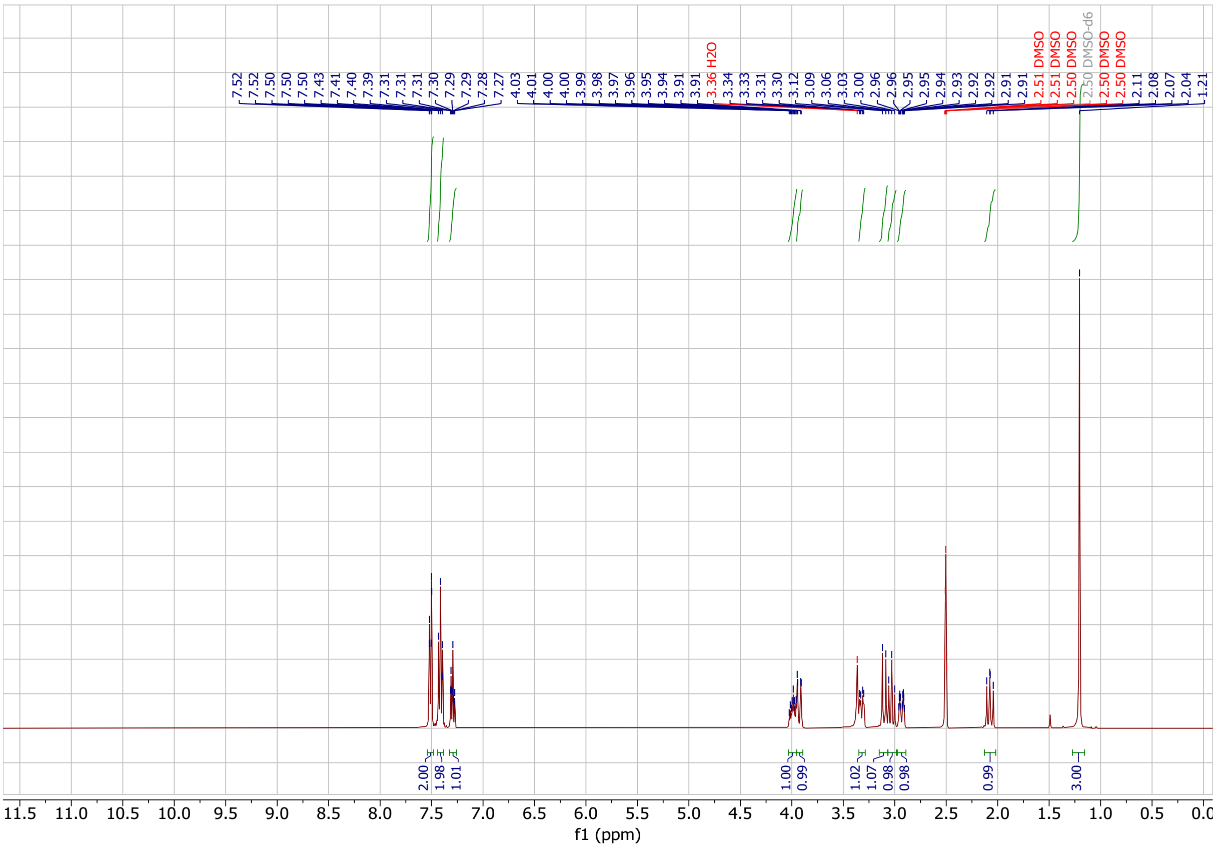

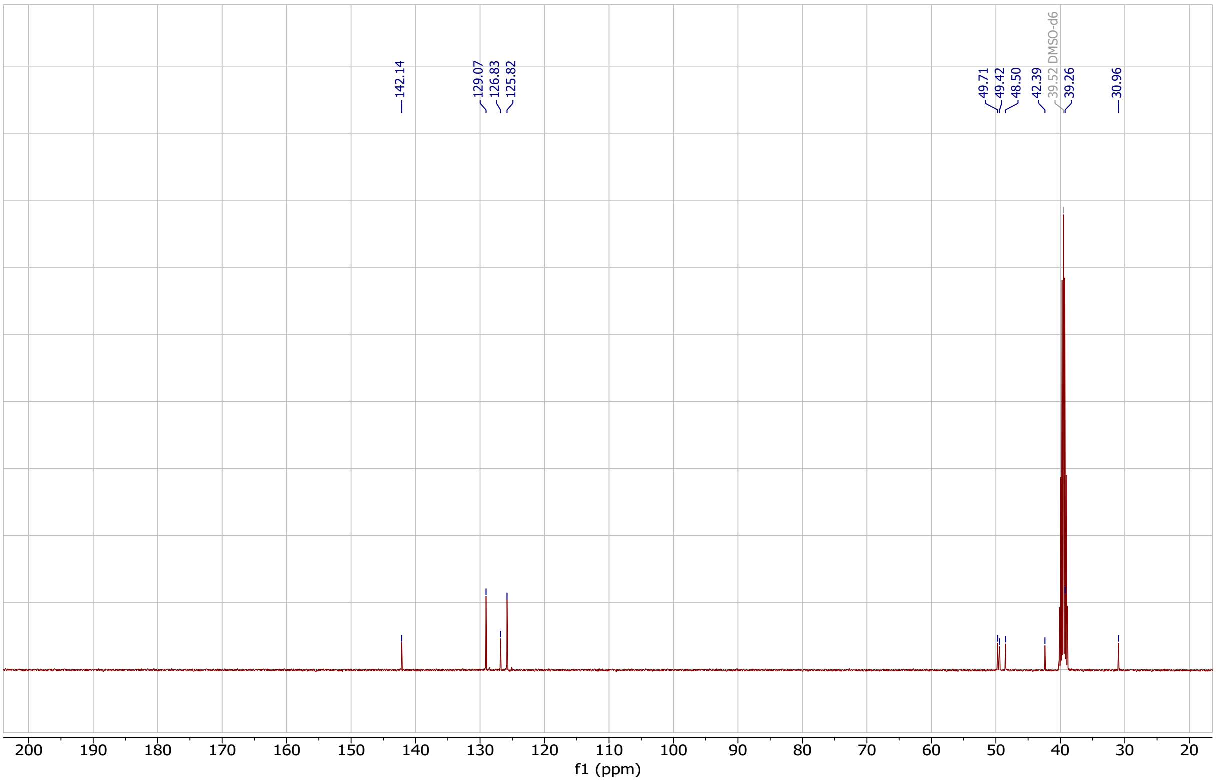

5-methyl-5-phenyl-1-azabicycle[3.1.0]hexane (11b)

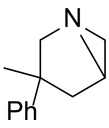

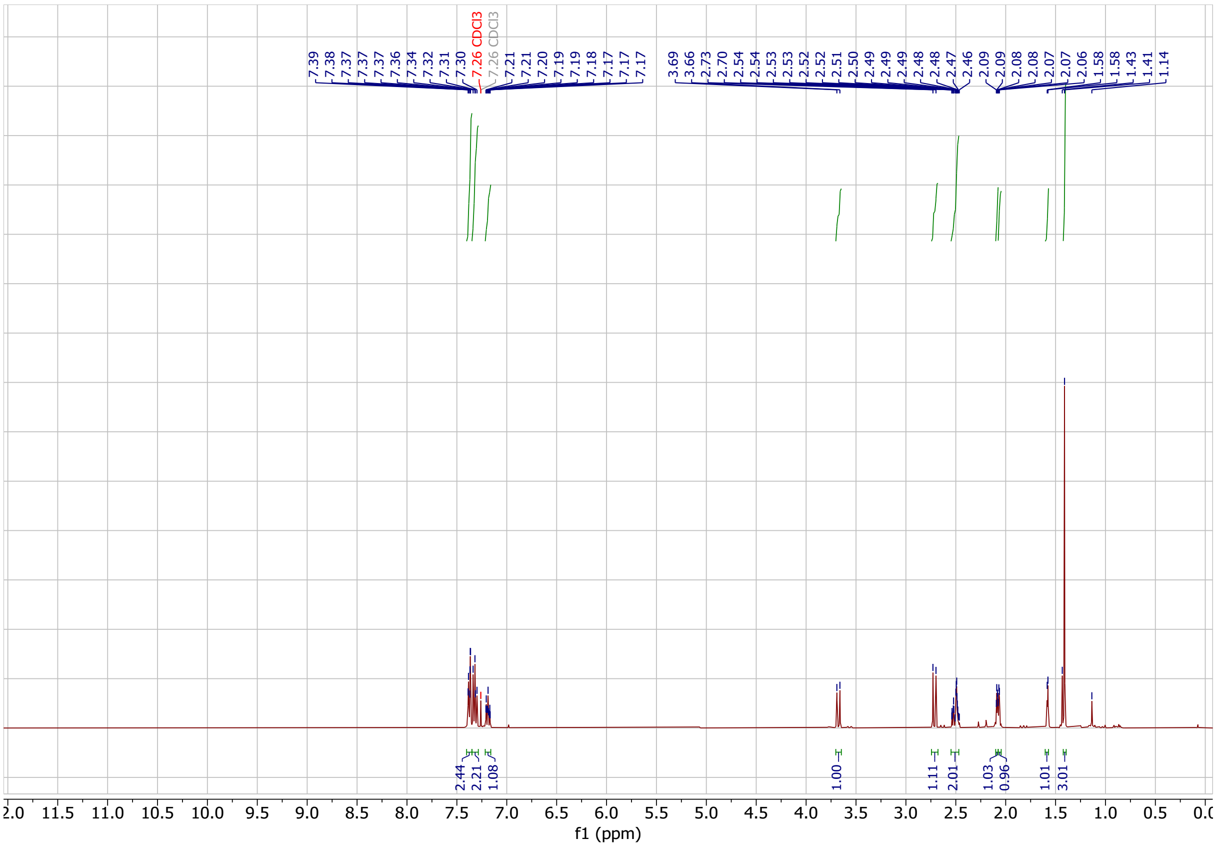

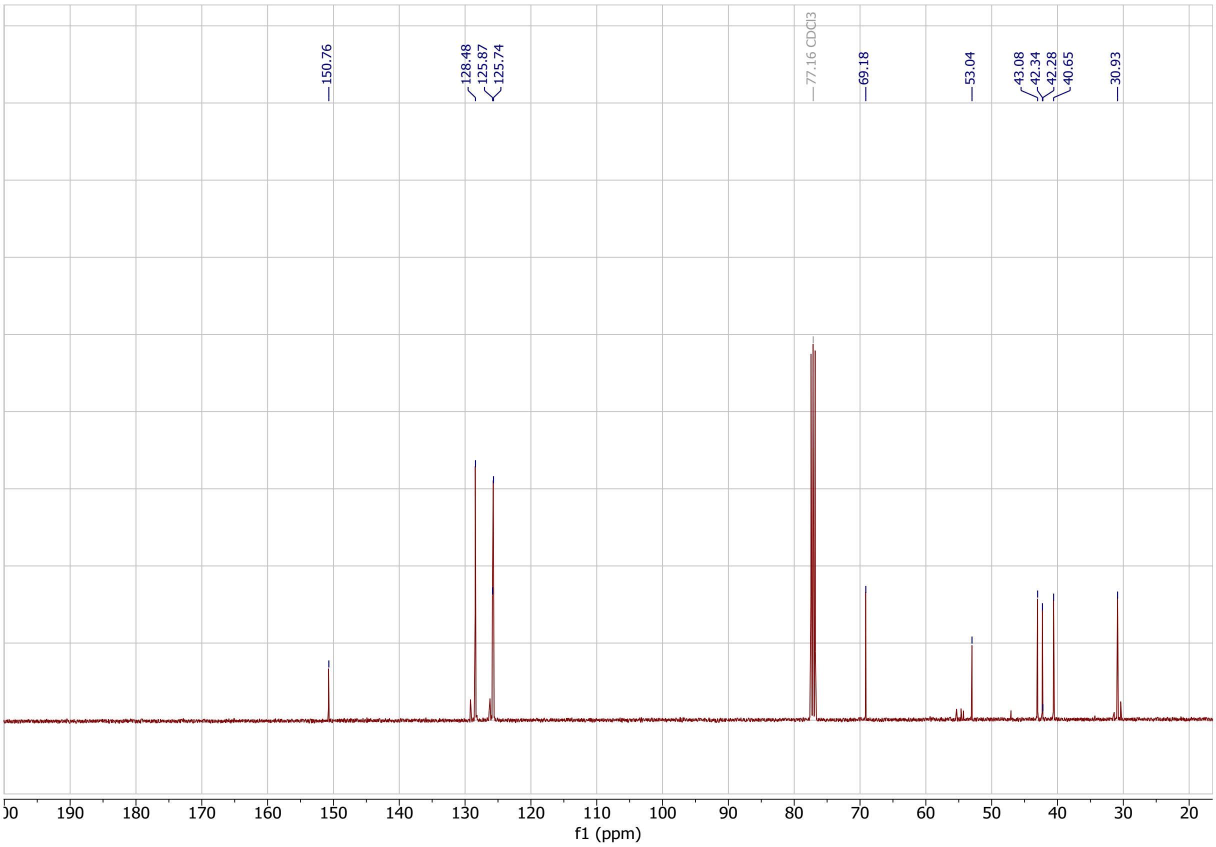

2,2-diphenylpent-4-en-nitrile

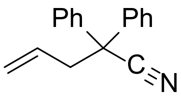

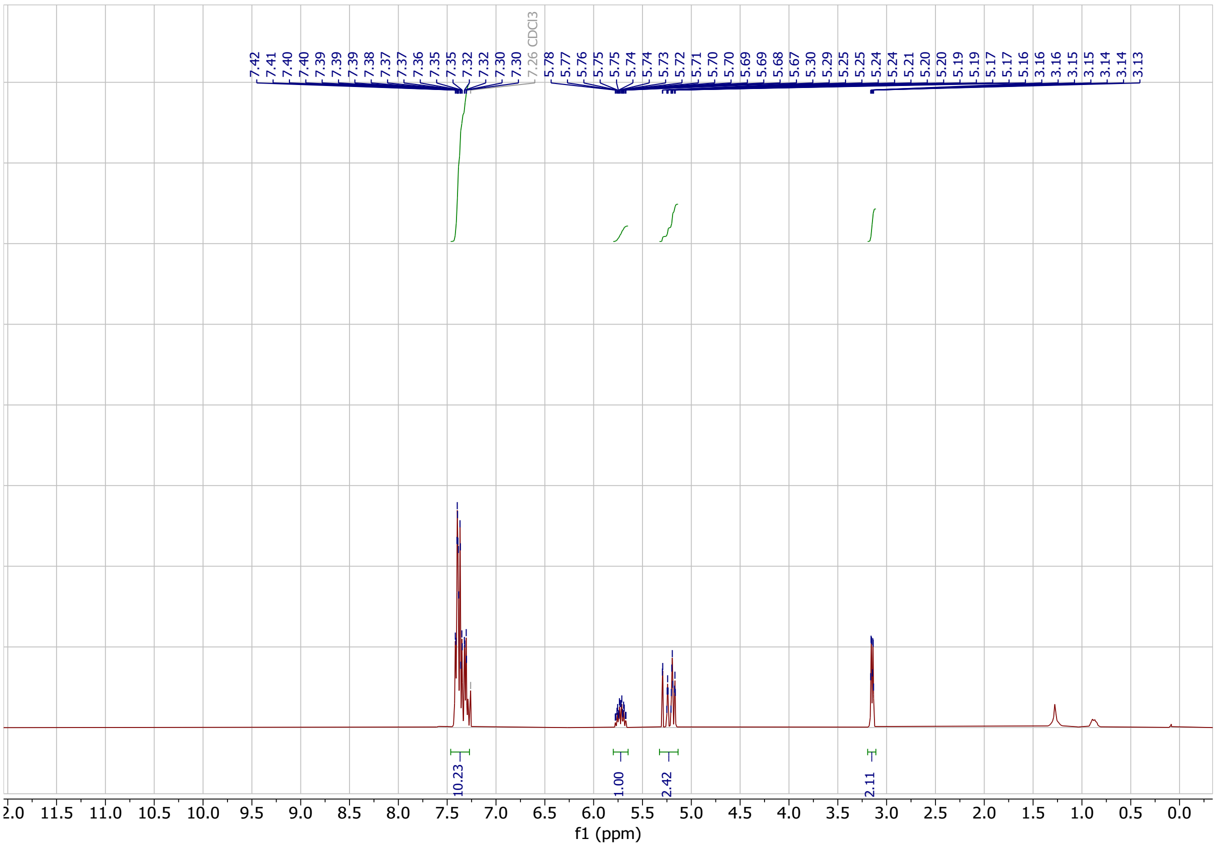

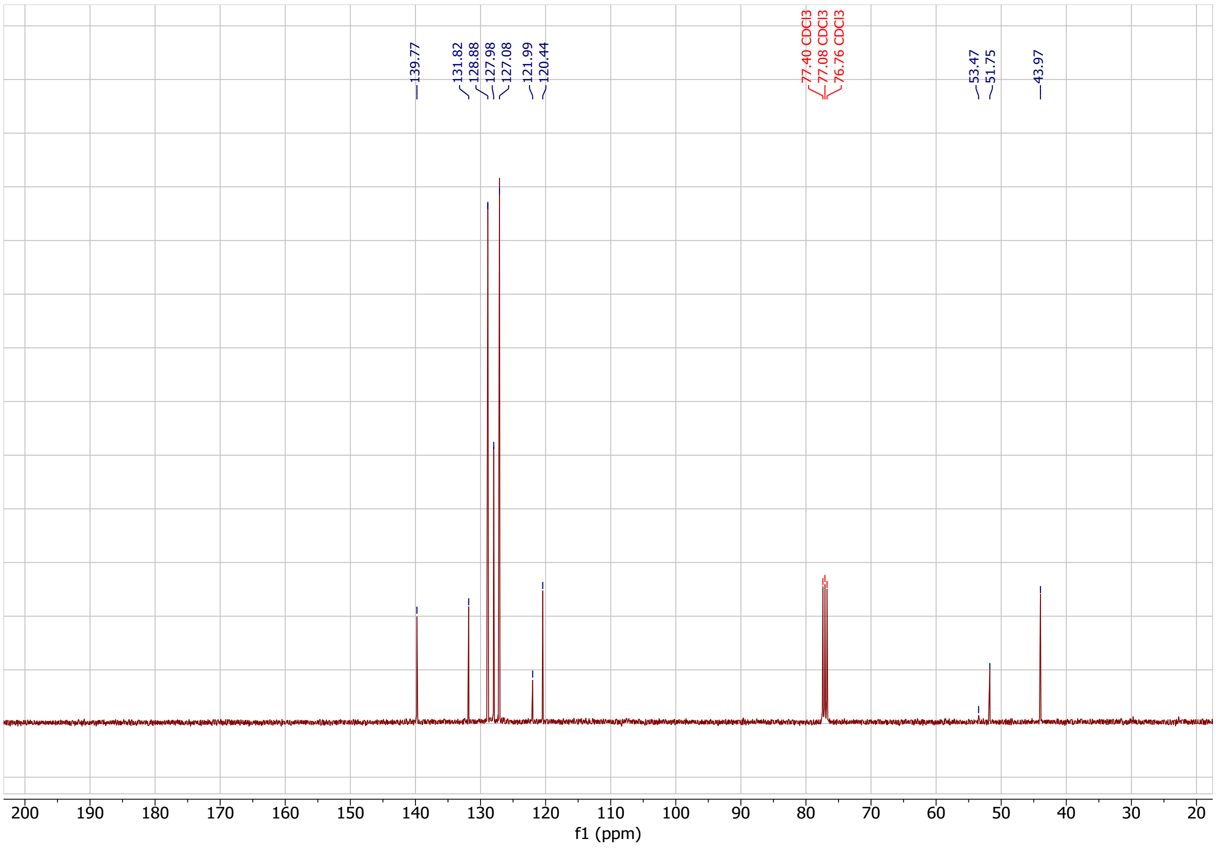

2,2-diphenylpent-4-en-1-amine (9c)

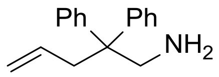

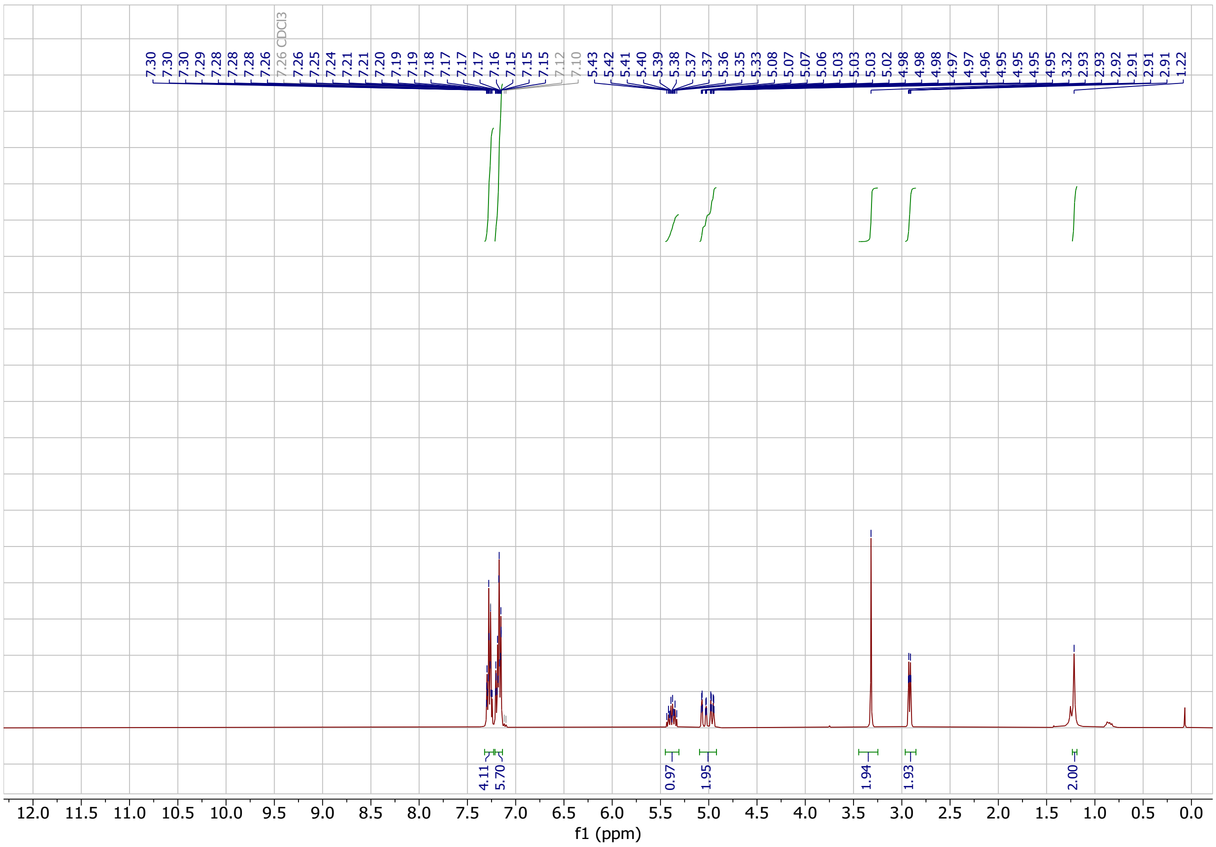

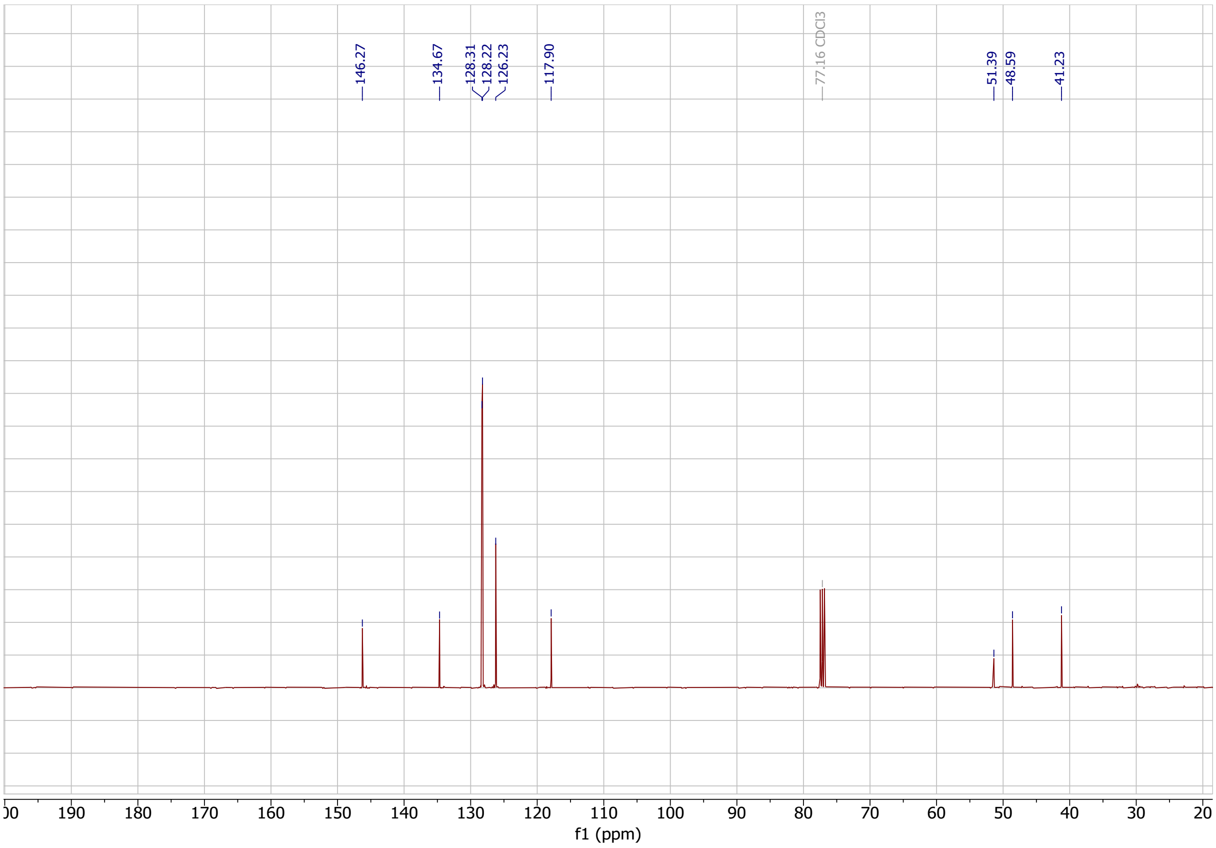

***N*-chloro-2,2-diphenylpent-4-en-1-amine (8c)**

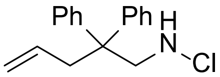

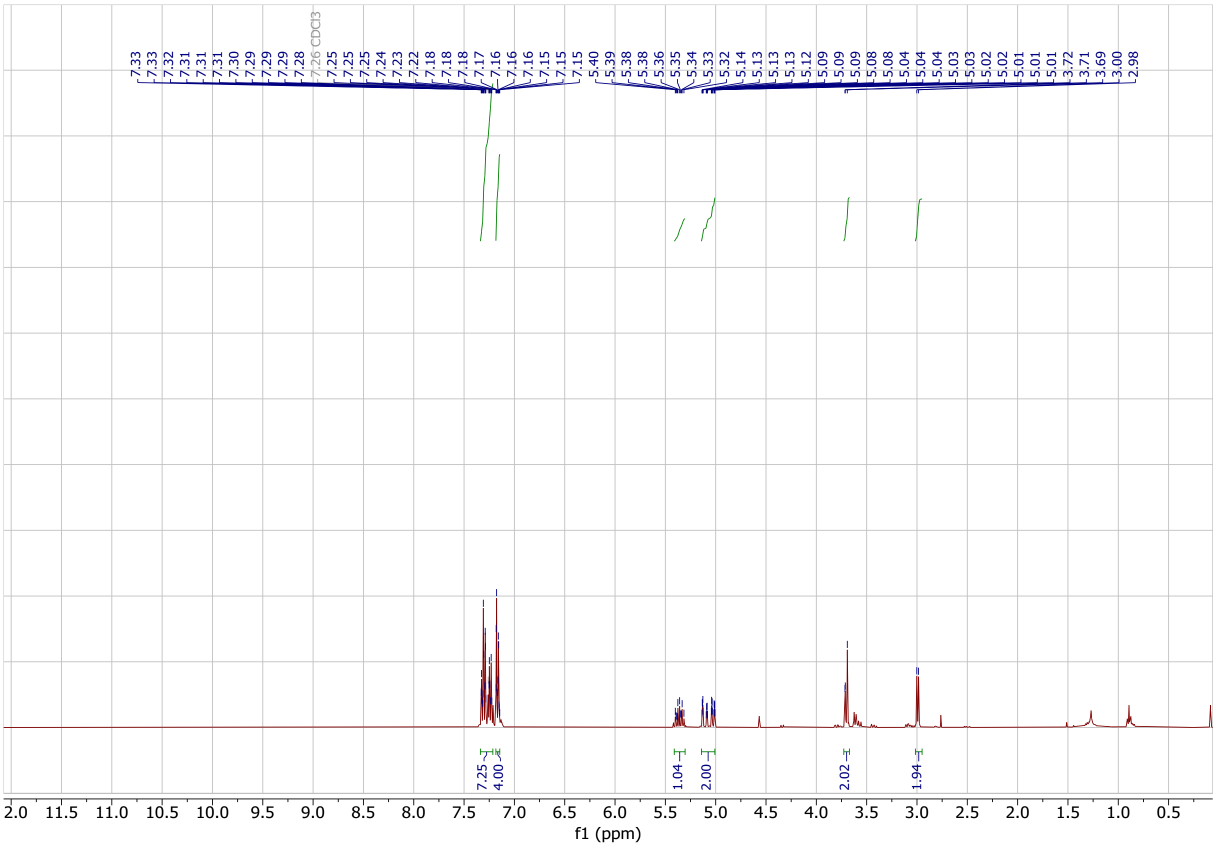

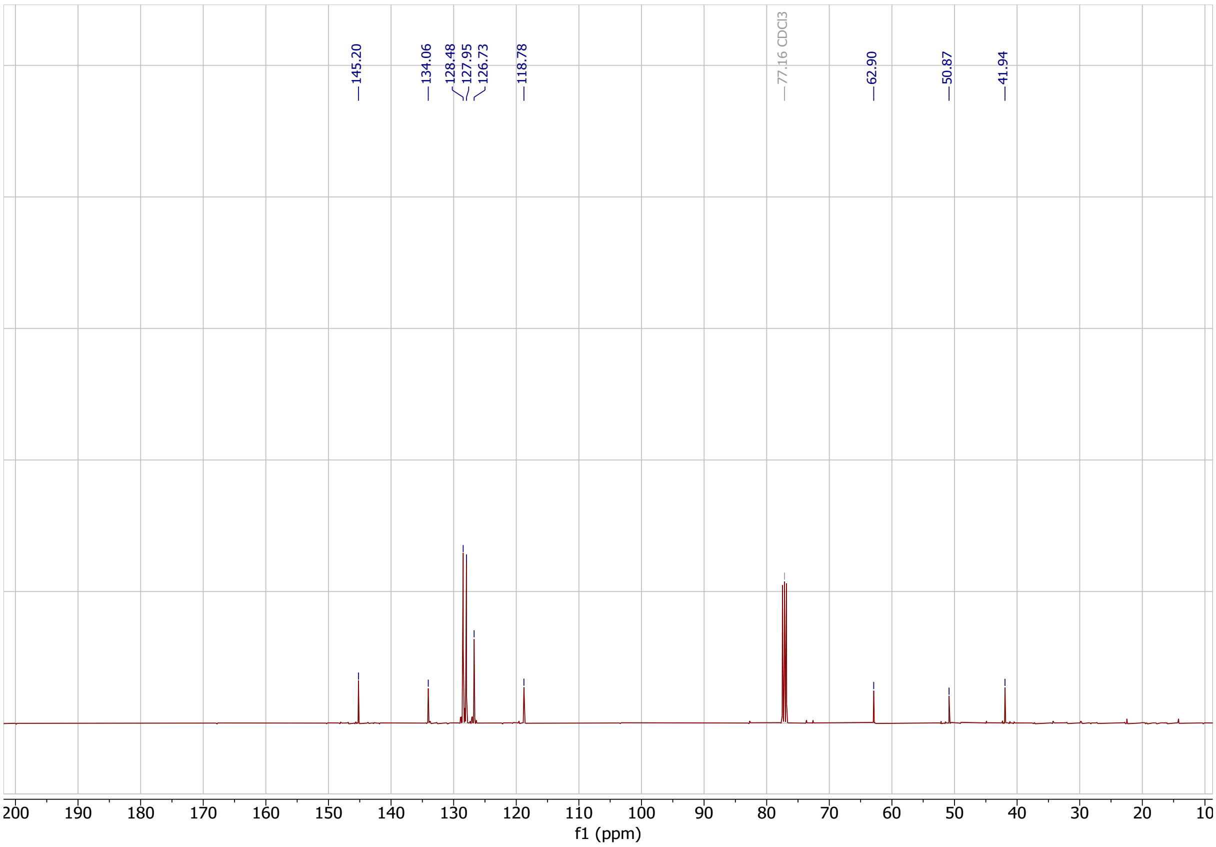

3-chloro-5,5-diphenylpiperidine (7c)

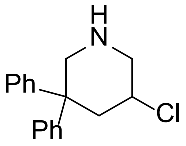

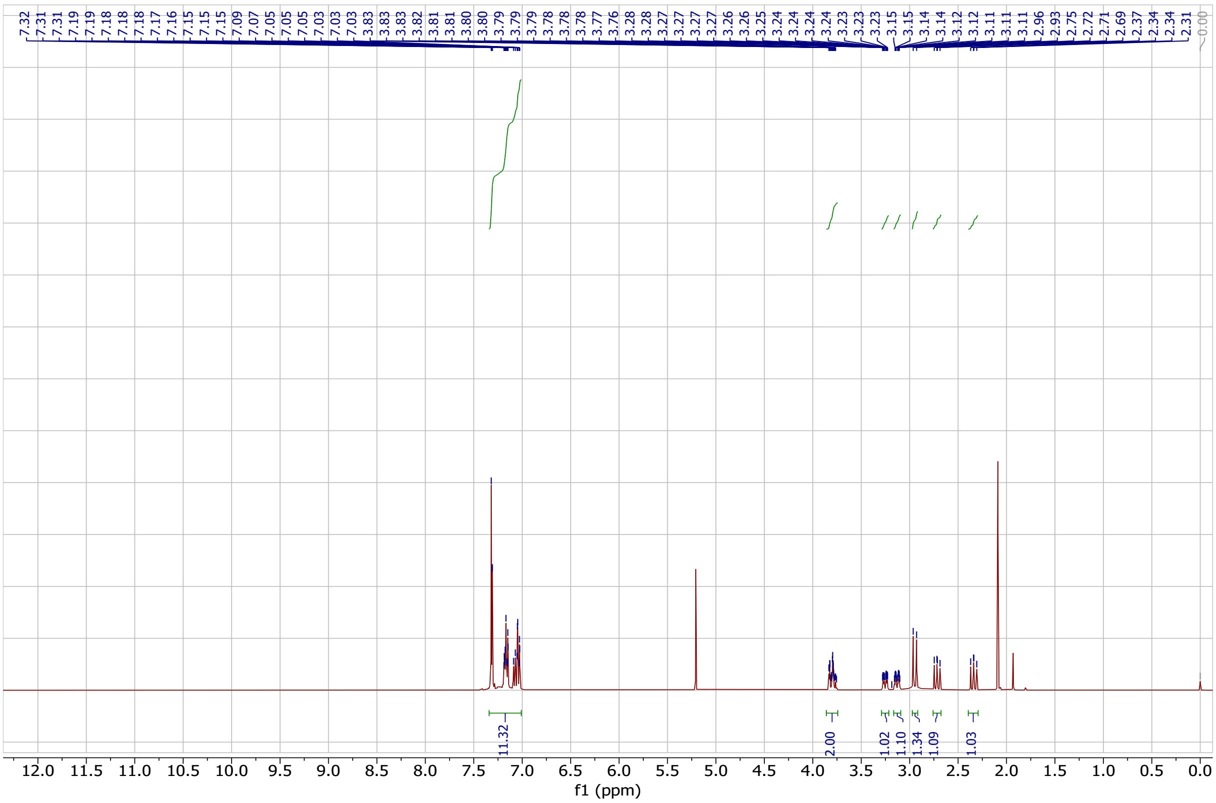

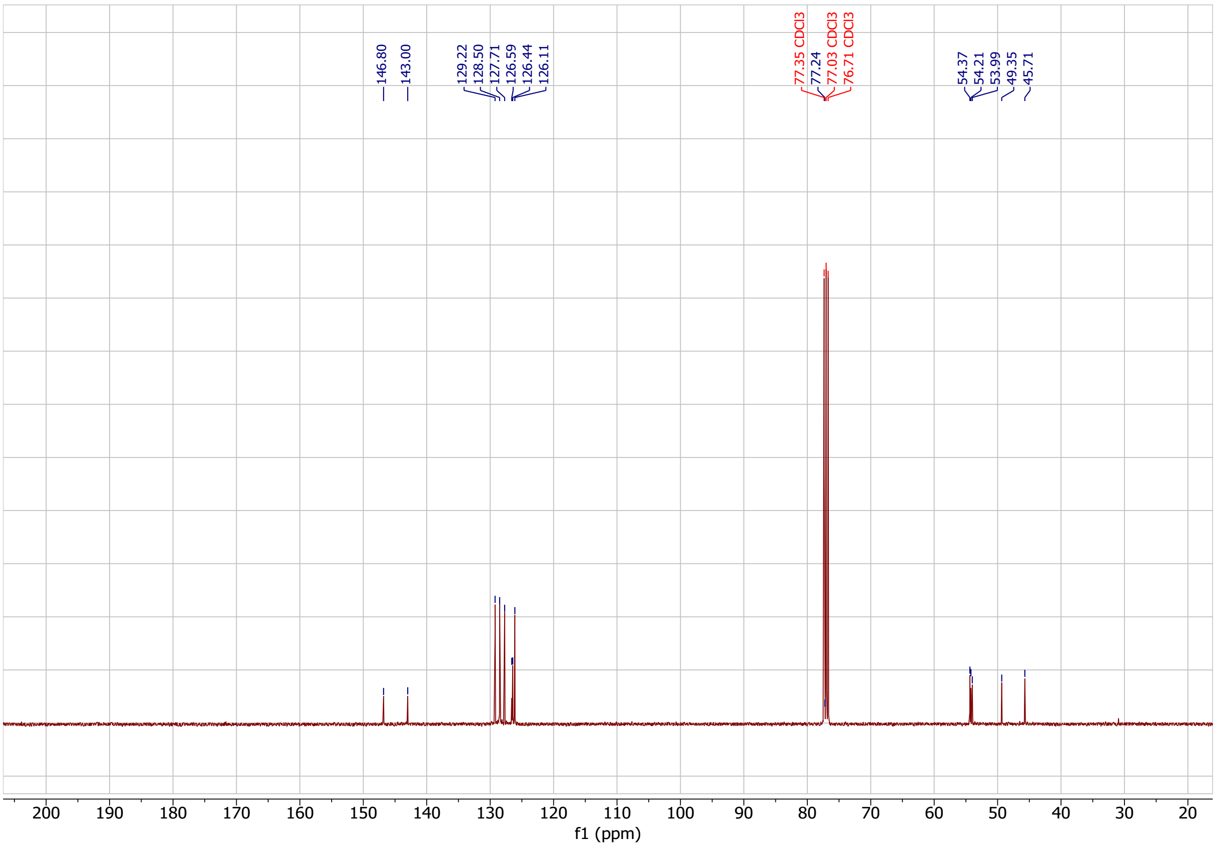

N-Boc-3-chloro-5,5-diphenylpiperidine (12c)

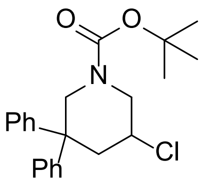

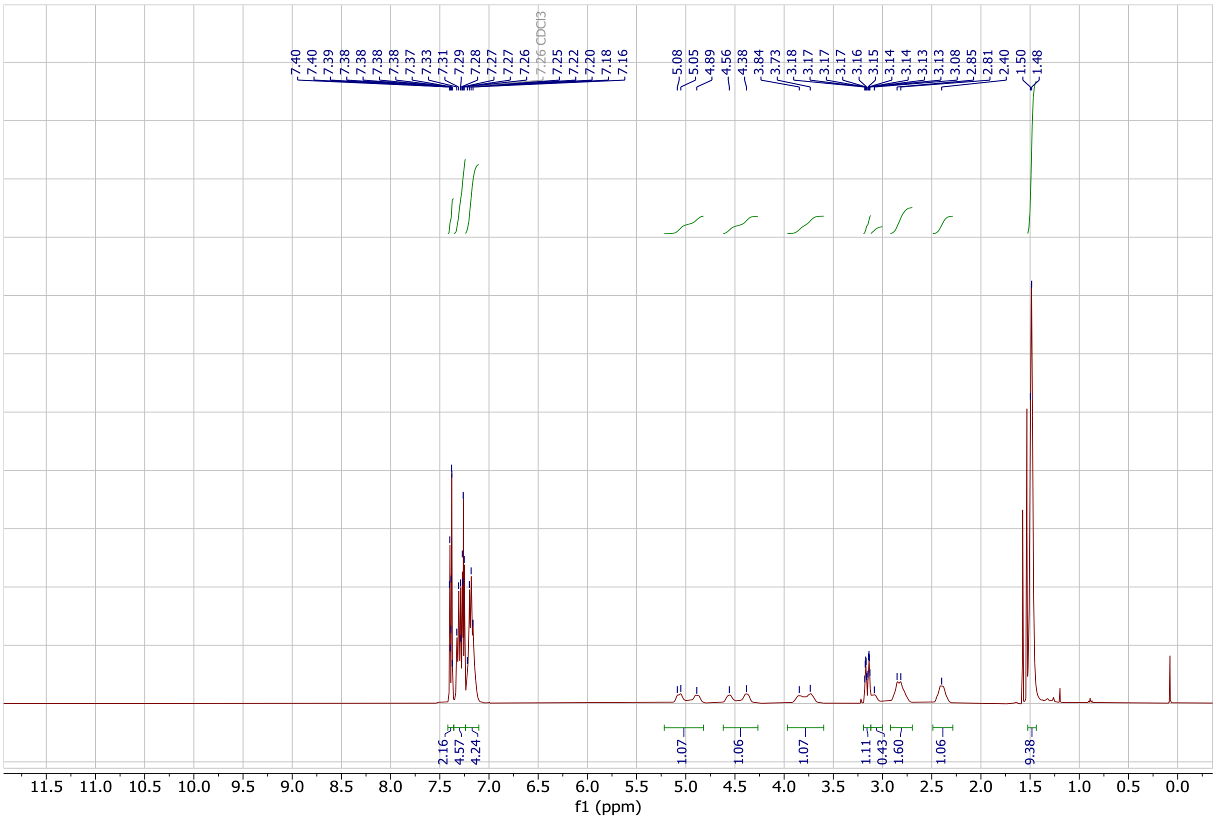

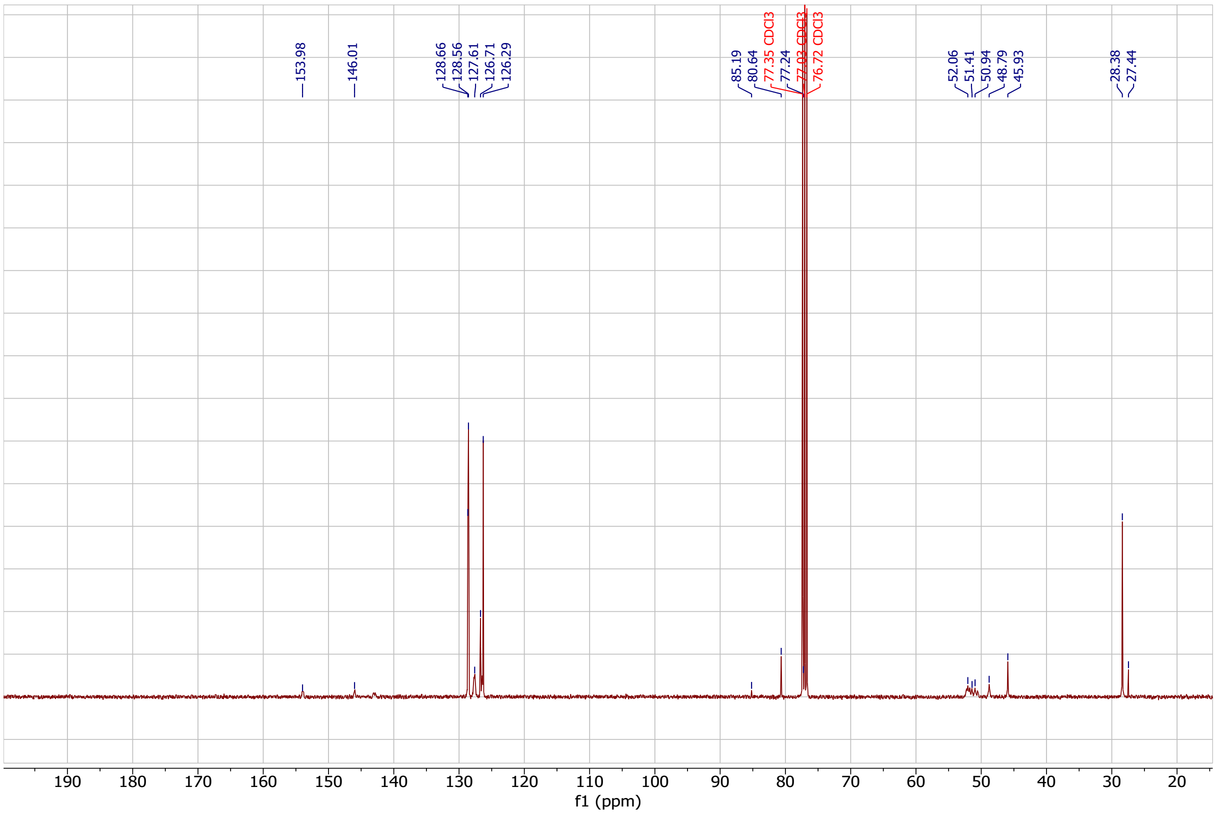

_N_-Boc-3-chloro-5,5-diphenylpiperidine (12c) – Pyrrolidine-Isomer

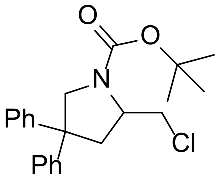

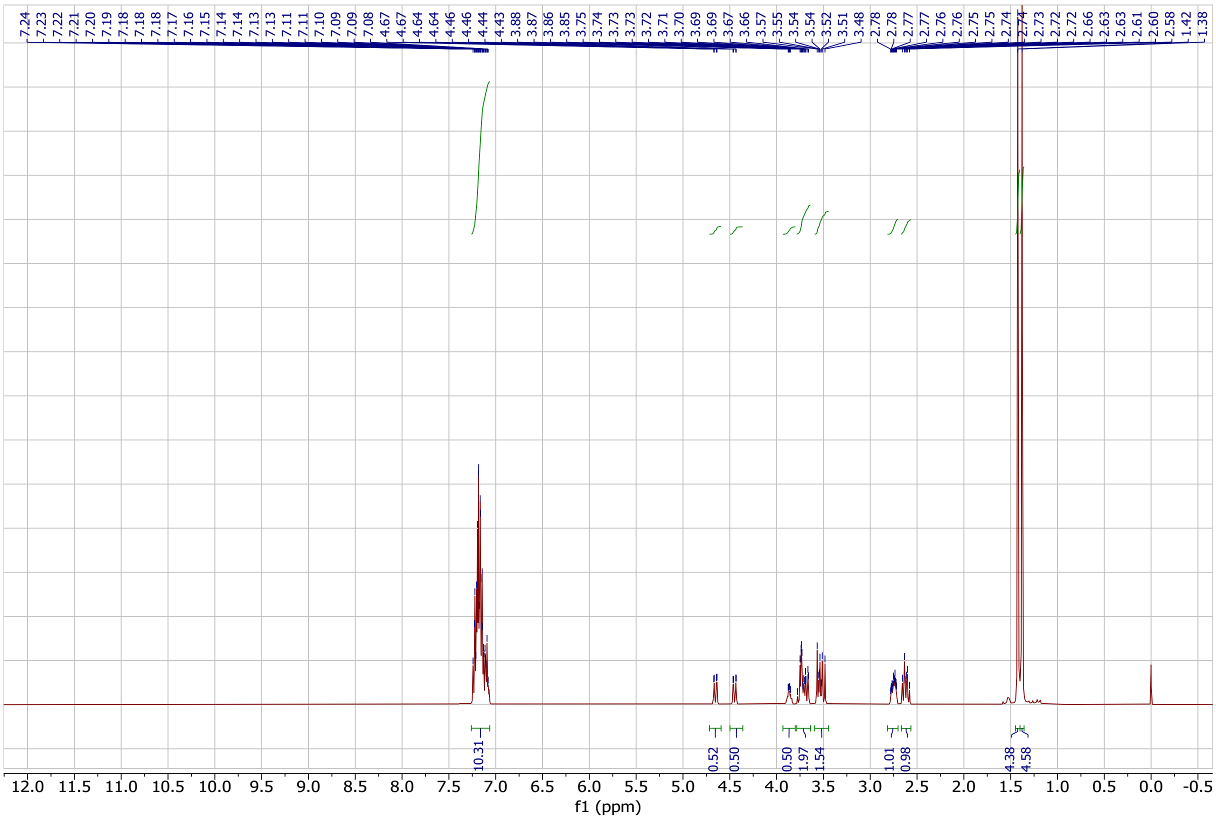

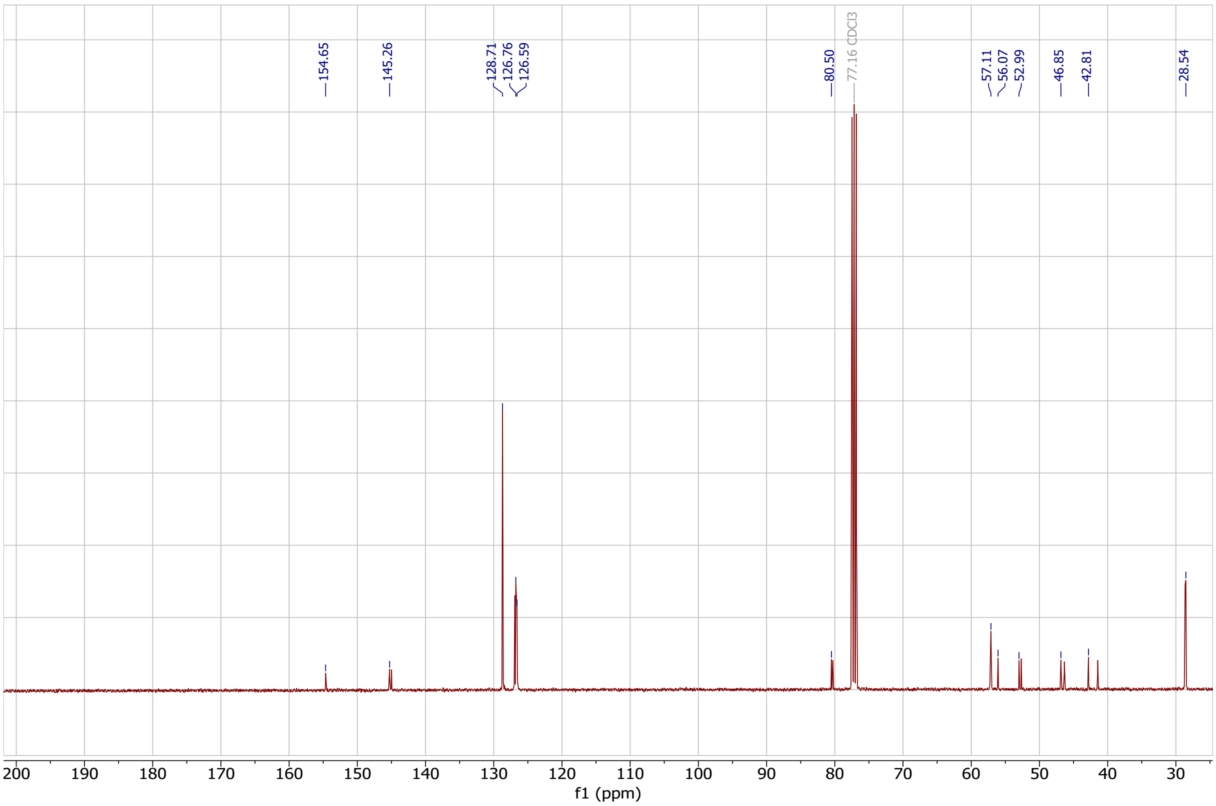

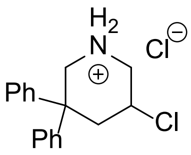

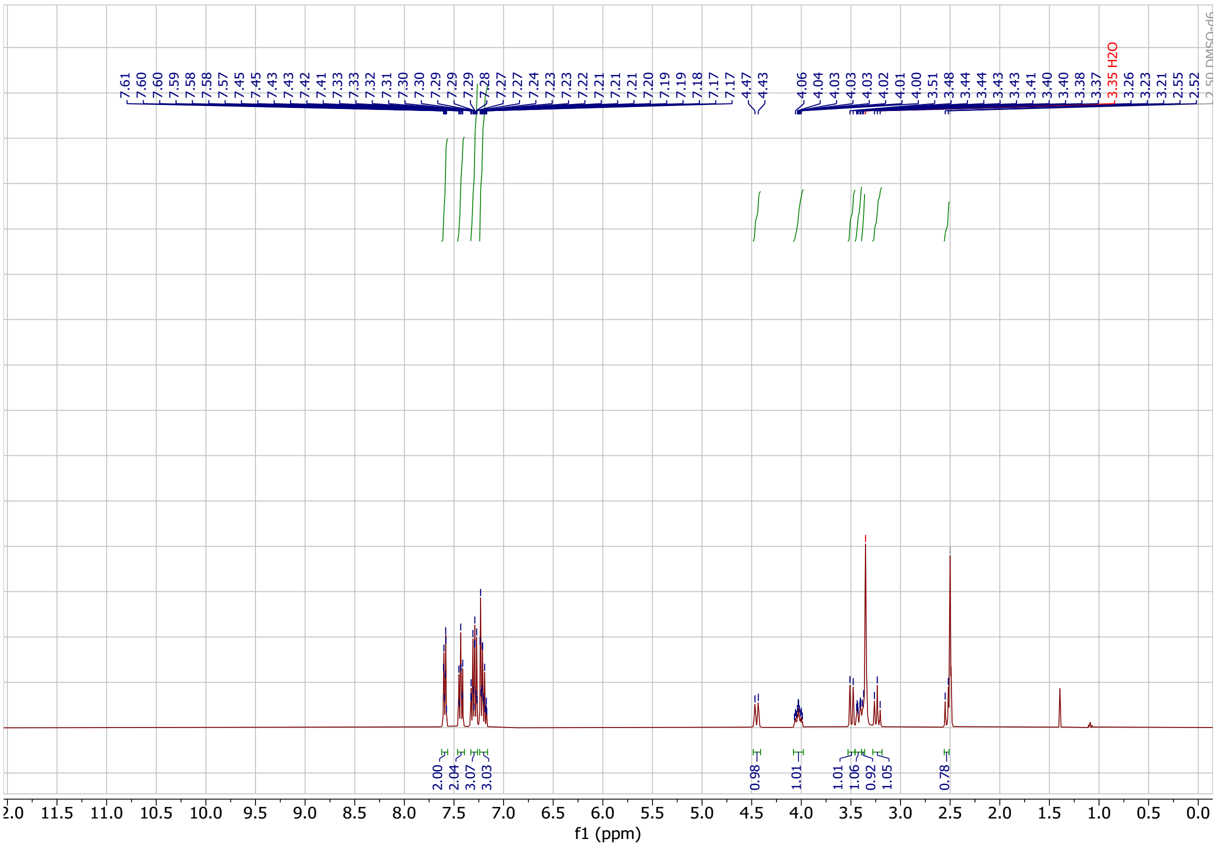

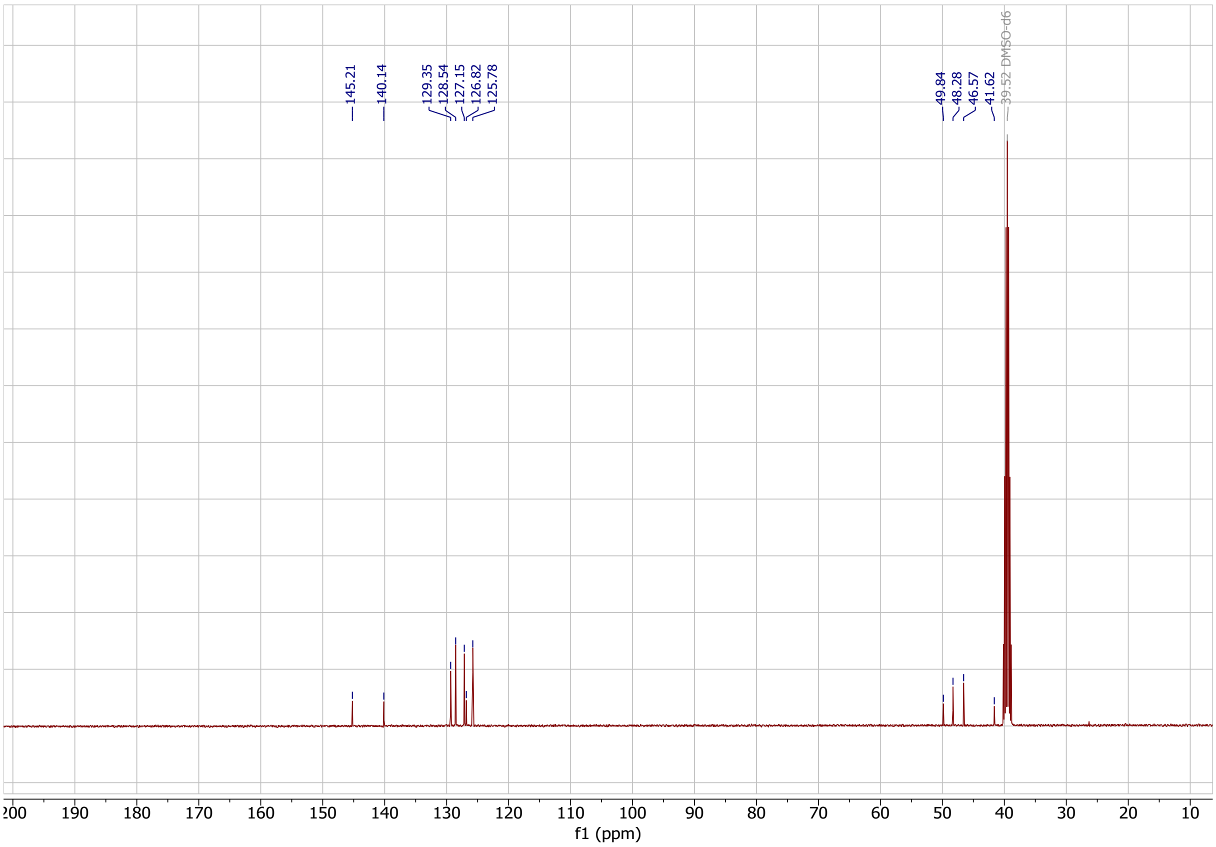

5,5-diphenyl-1-azabicycle[3.1.0]hexane (11c)

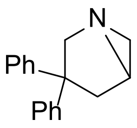

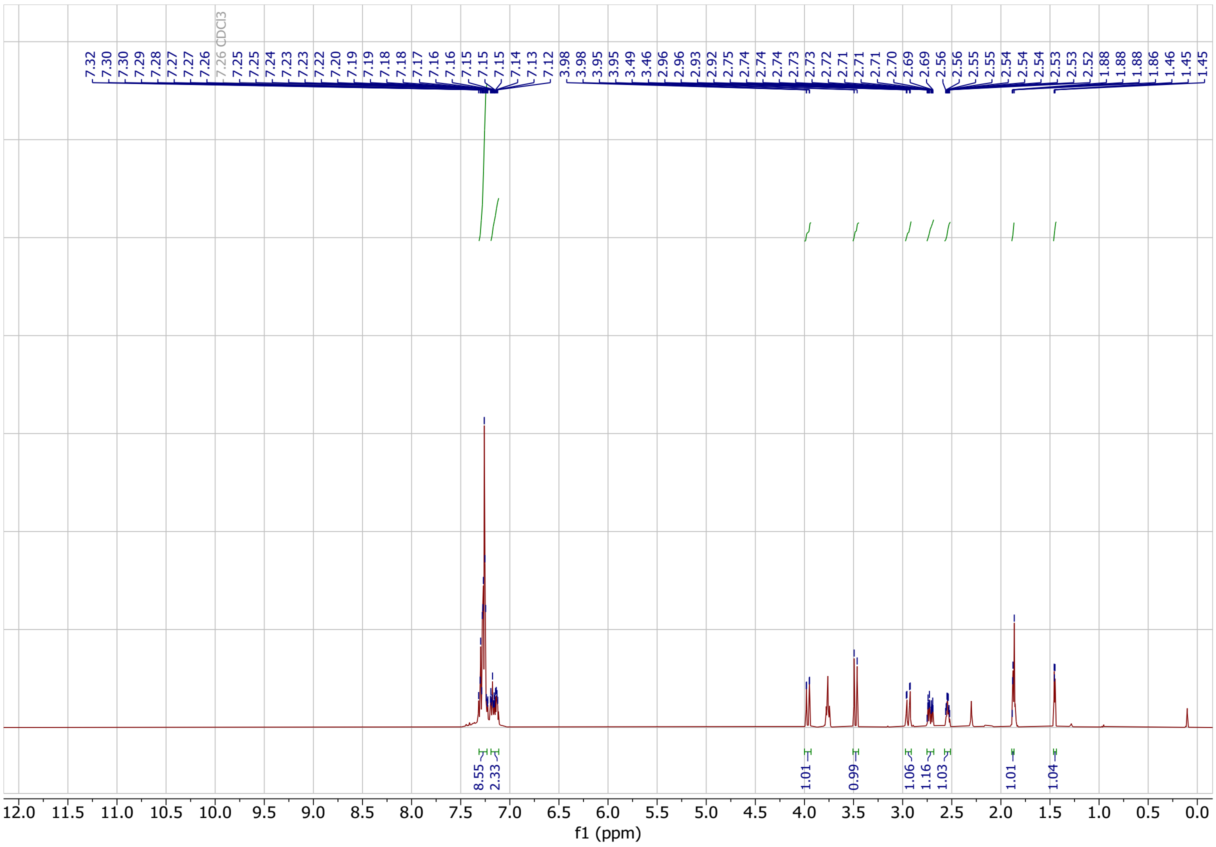

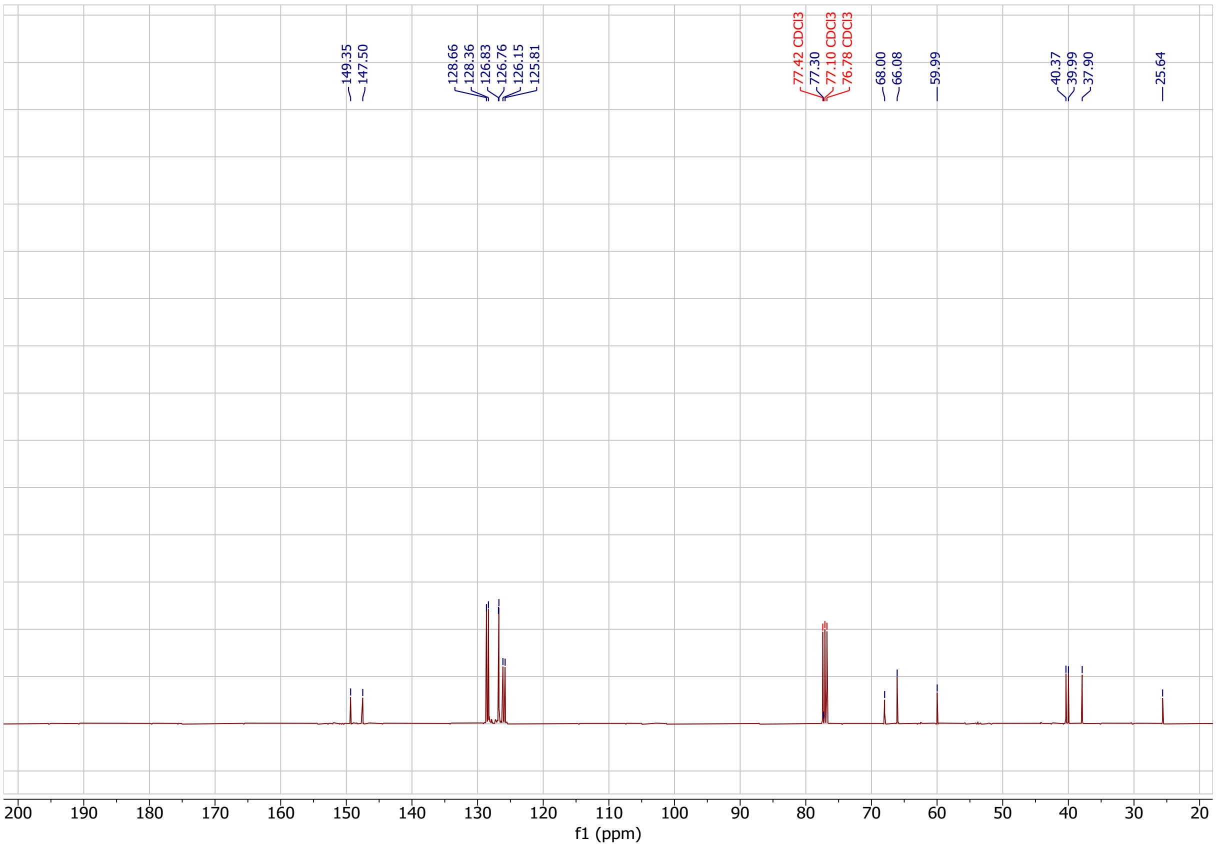

2,2-diethylpen-4-enal

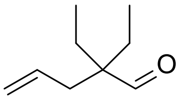

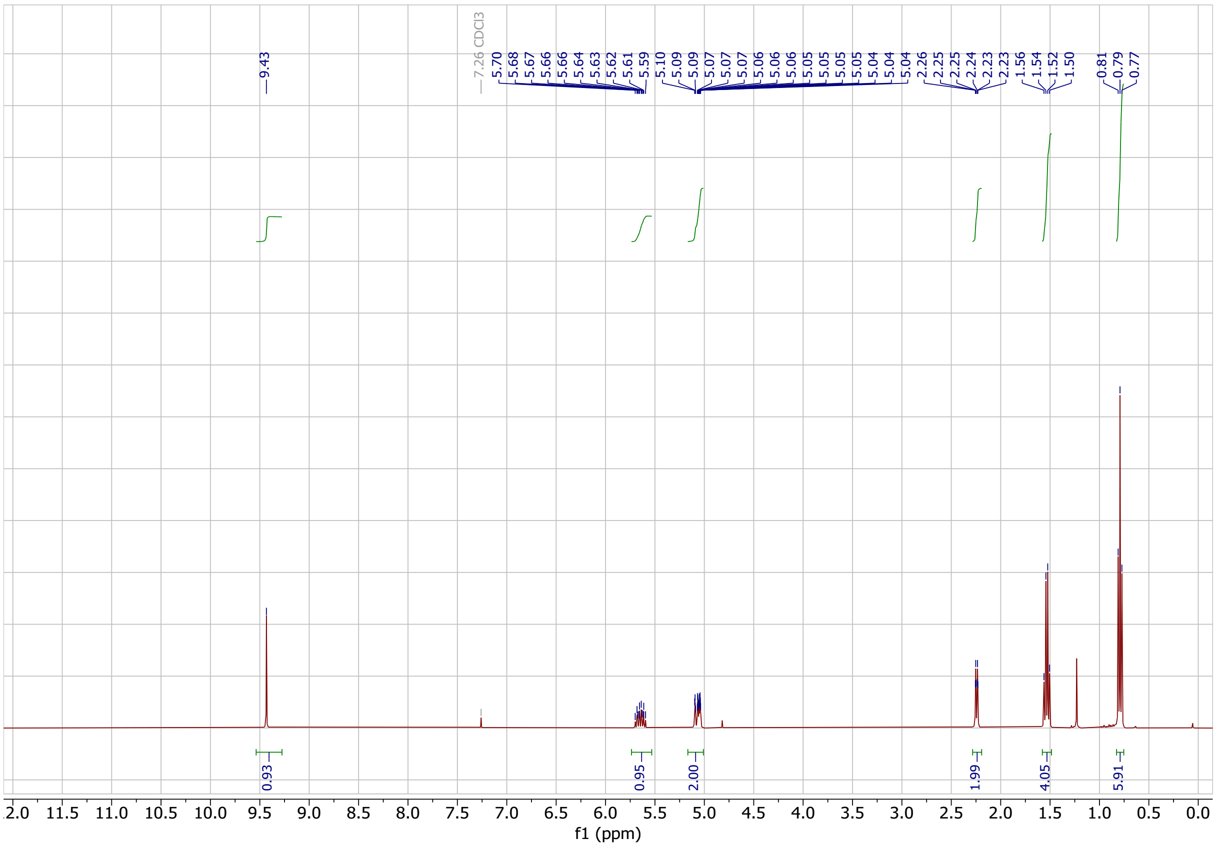

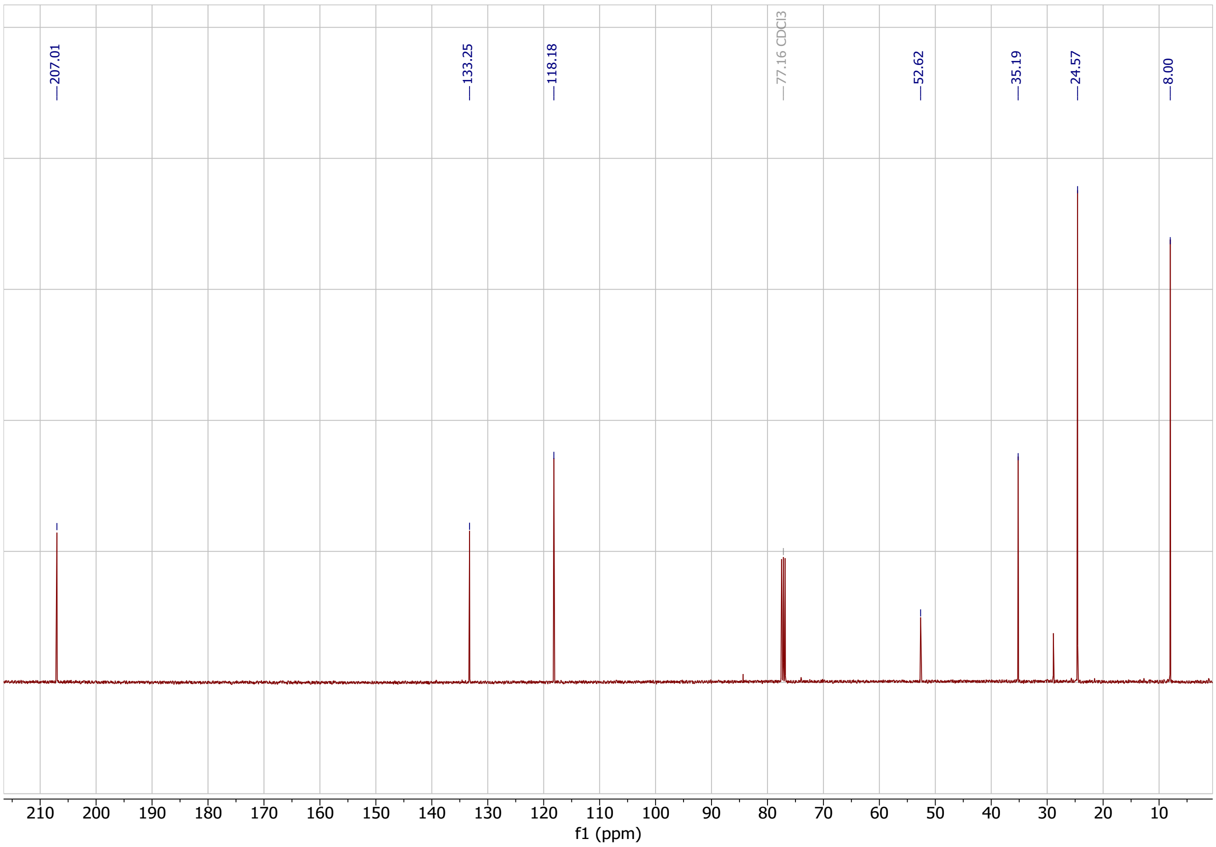

2,2-diethylpent-4-enal oxime

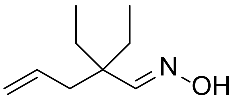

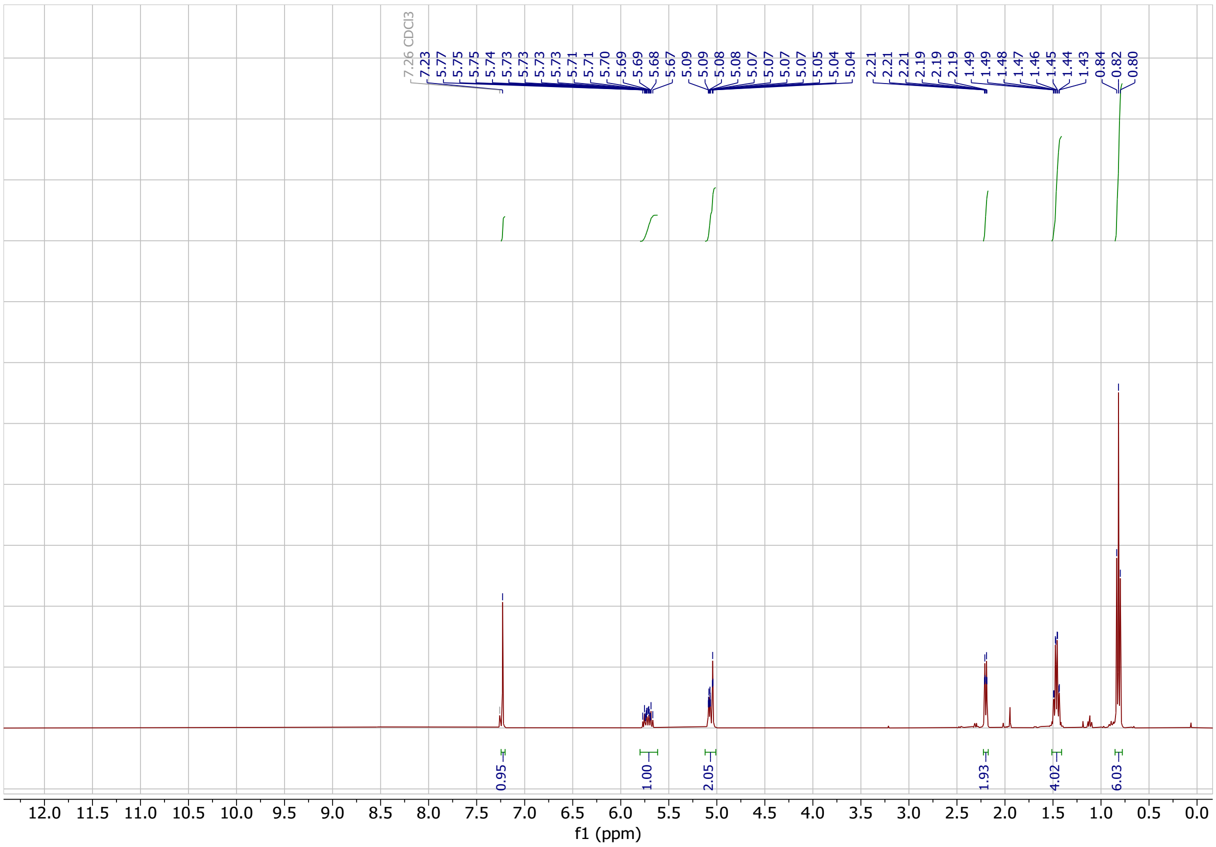

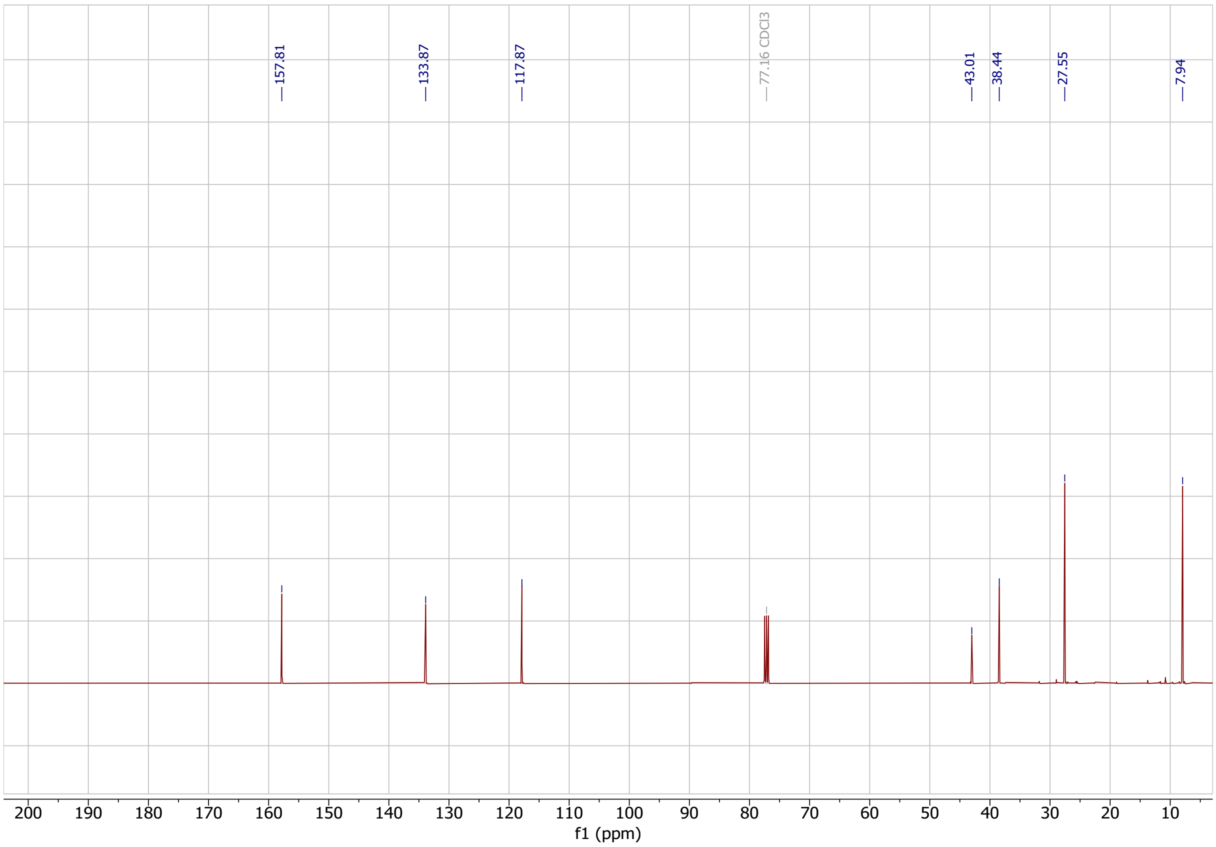

2,2-diethylpent-4-en-1-amine (9d)

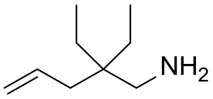

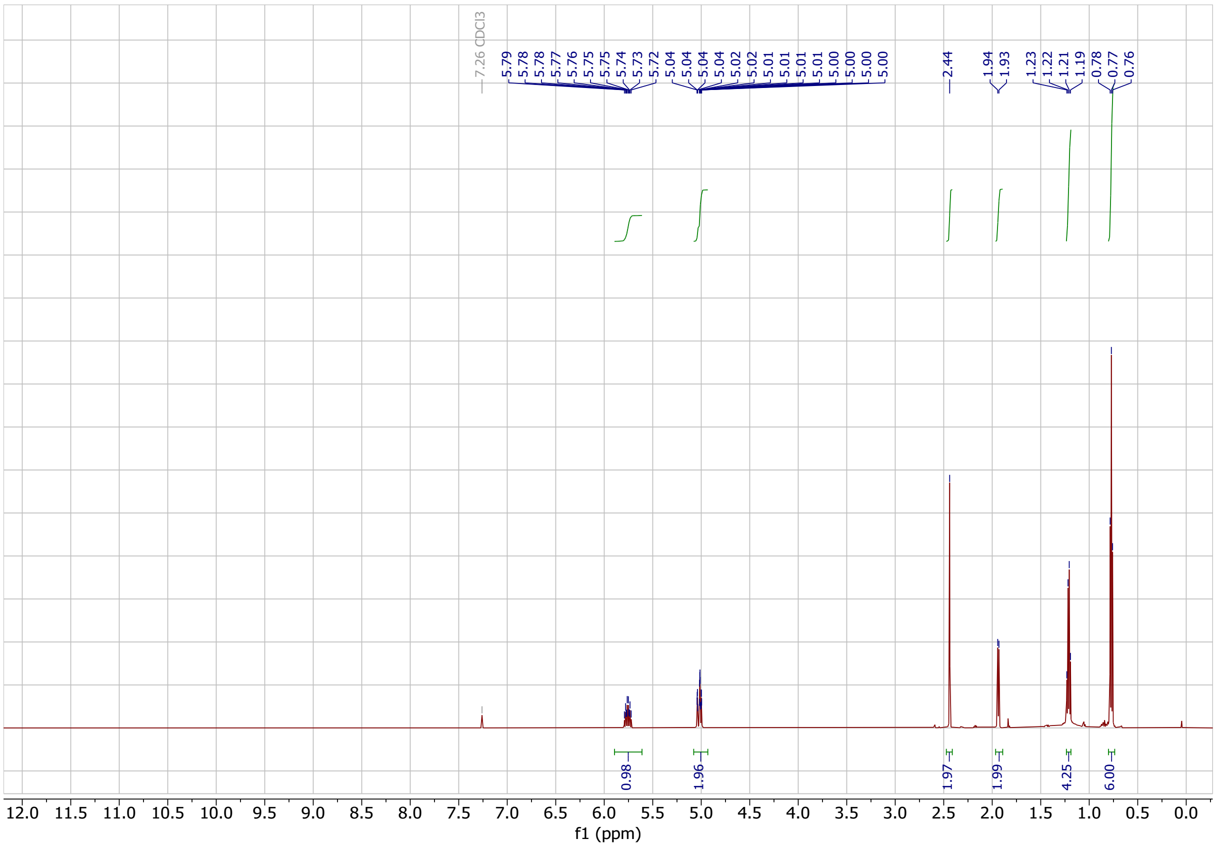

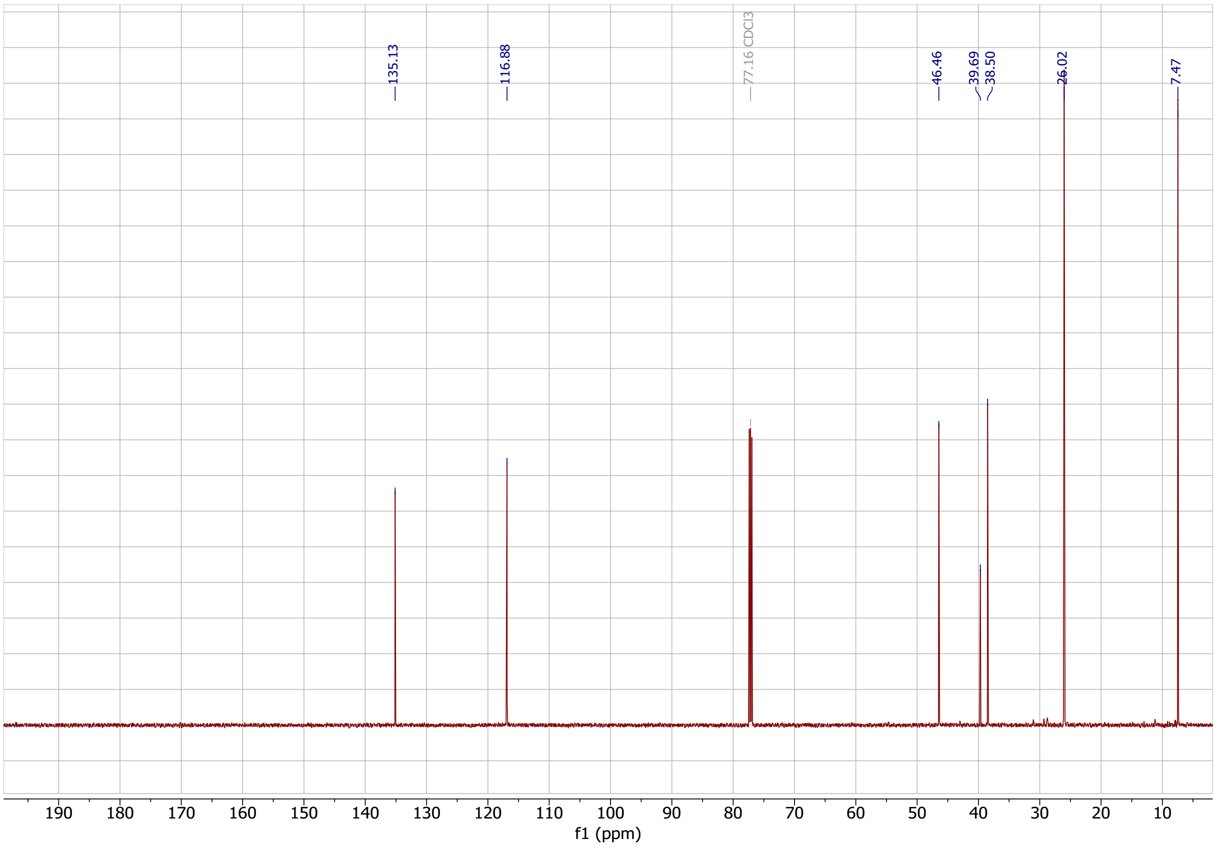

***N*-Boc-3-chloro-5,5-diethylpiperidine (12d)**

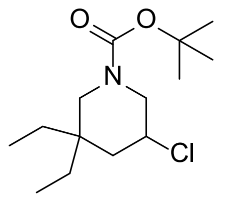

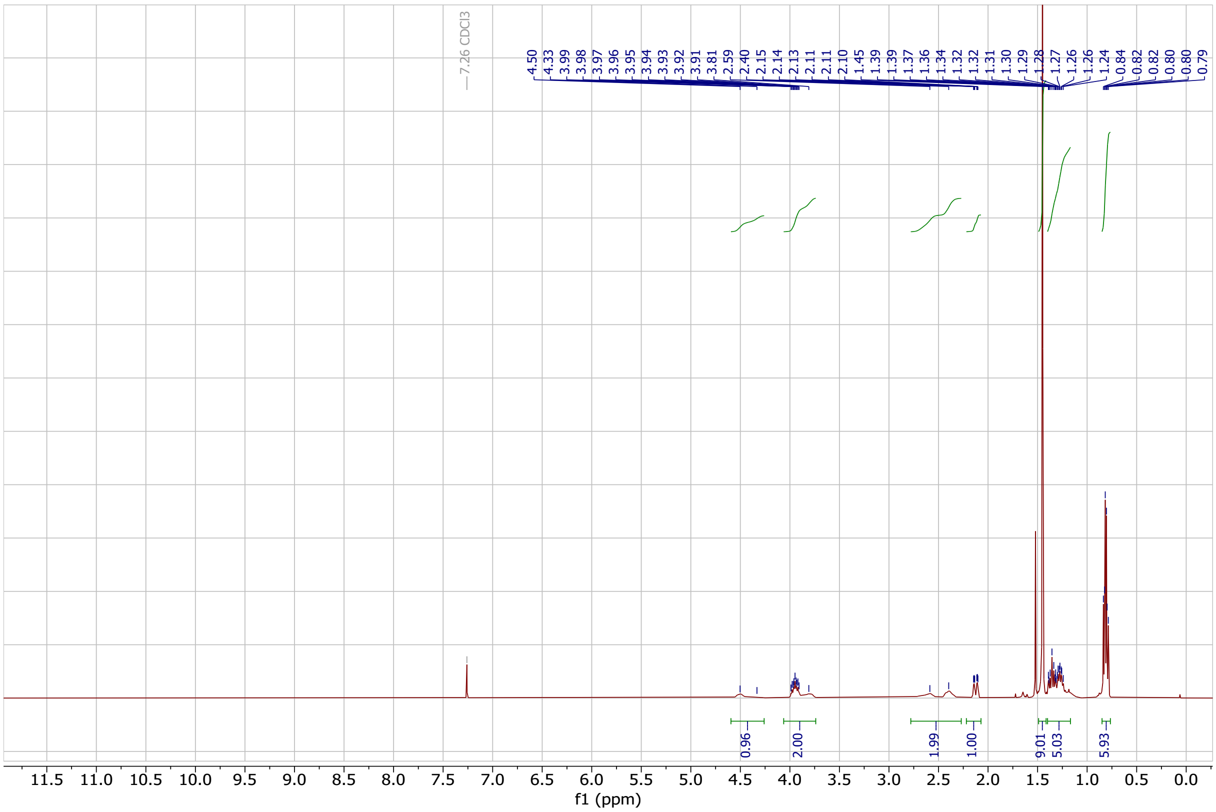

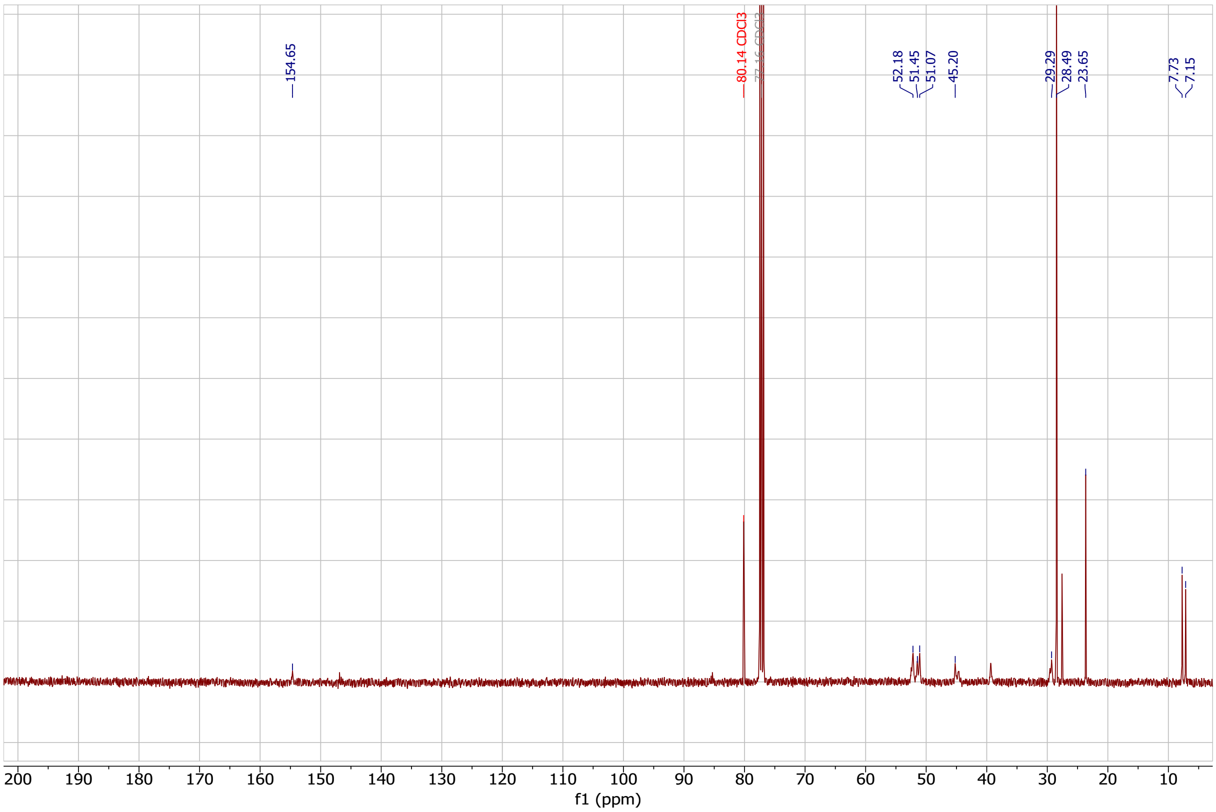

3-chloro-5,5-diethylpiperidine hydrochloride (13d)

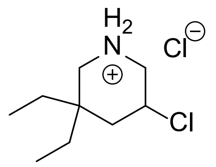

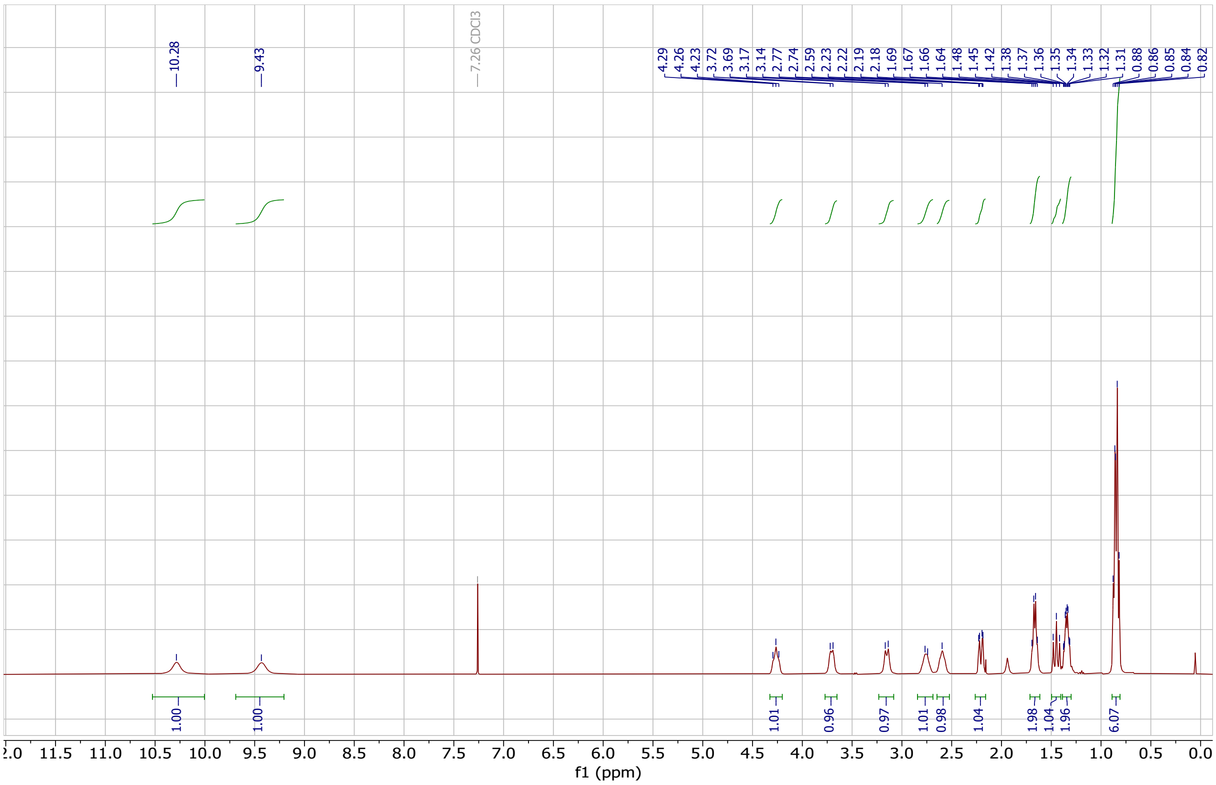

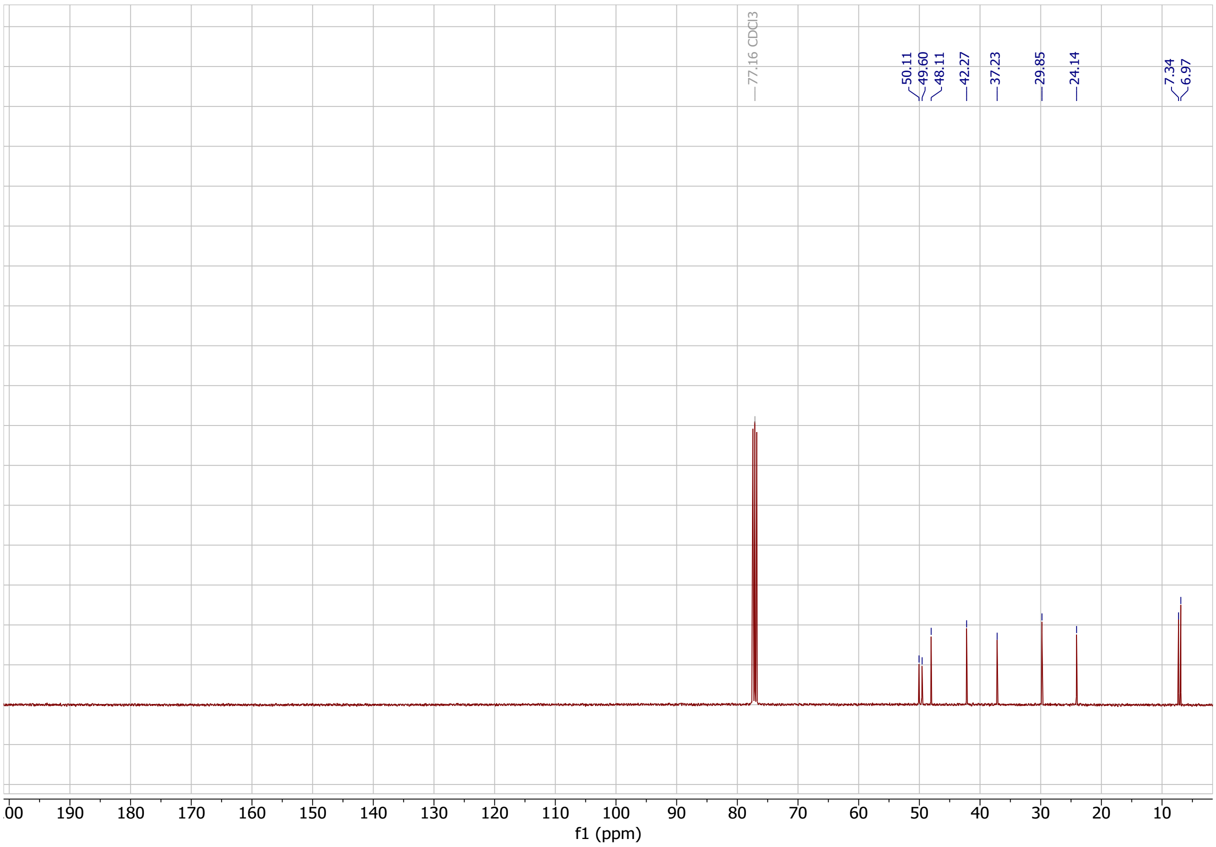

Supplement: Supplementary file 1 — Supporting Information [file OPEN-13-e202300181-s001.pdf]
